# In-silico clearing approach for deep refractive index tomography by partial reconstruction and wave-backpropagation

**DOI:** 10.1038/s41377-023-01144-z

**Published:** 2023-04-27

**Authors:** Osamu Yasuhiko, Kozo Takeuchi

**Affiliations:** Central Research Laboratory, Hamamatsu Photonics K.K, 5000 Hirakuchi, Hamakita-ku, Hamamatsu, 434-8601 Shizuoka Japan

**Keywords:** Interference microscopy, Imaging and sensing, Transmission light microscopy, Adaptive optics, Phase-contrast microscopy

## Abstract

Refractive index (RI) is considered to be a fundamental physical and biophysical parameter in biological imaging, as it governs light-matter interactions and light propagation while reflecting cellular properties. RI tomography enables volumetric visualization of RI distribution, allowing biologically relevant analysis of a sample. However, multiple scattering (MS) and sample-induced aberration (SIA) caused by the inhomogeneity in RI distribution of a thick sample make its visualization challenging. This paper proposes a deep RI tomographic approach to overcome MS and SIA and allow the enhanced reconstruction of thick samples compared to that enabled by conventional linear-model-based RI tomography. The proposed approach consists of partial RI reconstruction using multiple holograms acquired with angular diversity and their backpropagation using the reconstructed partial RI map, which unambiguously reconstructs the next partial volume. Repeating this operation efficiently reconstructs the entire RI tomogram while suppressing MS and SIA. We visualized a multicellular spheroid of diameter 140 µm within minutes of reconstruction, thereby demonstrating the enhanced deep visualization capability and computational efficiency of the proposed method compared to those of conventional RI tomography. Furthermore, we quantified the high-RI structures and morphological changes inside multicellular spheroids, indicating that the proposed method can retrieve biologically relevant information from the RI distribution. Benefitting from the excellent biological interpretability of RI distributions, the label-free deep visualization capability of the proposed method facilitates a noninvasive understanding of the architecture and time-course morphological changes of thick multicellular specimens.

## Abstract (web summary)

In-silico clearing refractive index tomography.

## Graphical abstract

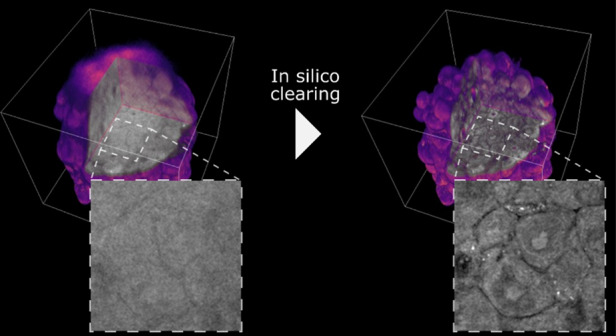

## Introduction

**Fig. 1:**
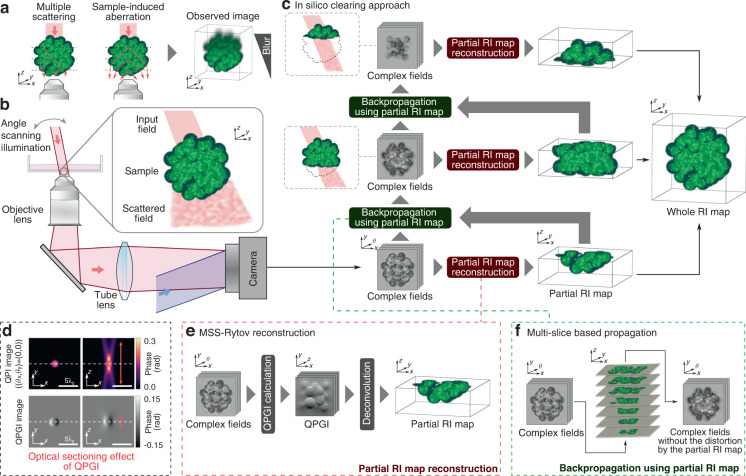
Overall working principle of in-silico clearing RI tomography. **a** Two detrimental effects, i.e., MS and SIA, degrade the resolving power of microscopes as the depth increases. **b** Schematic of the imaging system. Plane waves are irradiated onto a sample at various angles, and the diffracted light is interferometrically captured by a camera. **c** Pipeline of the in-silico clearing approach. In-silico clearing RI tomography reconstructs a partial RI map near the detection lens from the captured complex fields and computationally backpropagates the complex fields through the partial RI map. This operation eliminates wavefront distortion caused by the inhomogeneity of the partial RI map, suppresses MS and SIA, and enables unambiguous visualization of the next partial RI map. Repeating this operation till the top of the sample allows entire high-resolution reconstruction. **d** Quantitative phase imaging (QPI) and quantitative phase gradient imaging (QPGI) of a homogeneous sphere. Images obtained by QPI have a long tail along the optical axis, whereas those obtained by QPGI have a short tail, enabling selective visualization of the sample at a certain depth. **e** Partial RI map reconstruction using MSS-Rytov, which was proposed previously. MSS-Rytov calculates through-focus QPGI images from multi-angle complex fields. This reconstruction scheme takes advantage of the optical sectioning effect of QPGI. **f** Backpropagation using the partial RI map. In the proposed approach, captured complex fields are backpropagated through the partial RI map by multi-slice-based propagation

Optical imaging has been used to elucidate various biological and physiological phenomena in living specimens with minimal invasiveness and a resolving power sufficiently high to visualize subcellular structures. However, optical imaging only achieves low imaging depth owing to multiple scattering (MS) and sample-induced aberration (SIA), caused by the inhomogeneity of the refractive index (RI) of thick samples^1^ (Fig. 1a). MS attenuates single-scattering (SS) light by inducing severe wavefront distortion, thereby concealing the SS light in the MS background. In contrast, SIA broadens the width of the point spread function by causing relatively slow varying wavefront distortion, degrading the imaging resolution and sensitivity. Therefore, overcoming MS and SIA is key to realizing high-resolution deep optical imaging^1^.

A representative approach for suppressing MS is to selectively detect SS waves using temporal or spatial gating operations, as implemented in optical coherence tomography^2^ (OCT) or confocal microscopy, respectively. Additionally, full-field OCT^3,4^ realized high-spatial-resolution imaging via spatial and temporal gating leveraging low-coherent spatial and temporal interference. A computational spatial gating method has also been proposed^5^. The performance of the gating operation depends on the efficiency of extracting constructively interfering SS photons from MS photons. Unfortunately, SIA significantly deteriorates the spatial gating performance due to the loss of spatial coherency. SIA is generally addressed using adaptive optics (AO)^6,7^. Most AO methods employ dynamic correction elements, such as deformable mirrors or spatial light modulators, to introduce compensatory distortion of SIA. SIA is usually estimated by direct sensing, a wavefront sensor or guide star, or by the indirect optimization of the sharpness metric calculated from multiple images. AO has become a valuable tool in various imaging techniques, such as confocal microscopy and multi-photon microscopy^8^. However, when imaging deep within samples or for samples with a large surface curvature, a single aberration correction works well only in the vicinity of the aberration measurement location, called the isoplanatic patch, owing to the spatially varying SIA^7^. This makes it difficult to efficiently and accurately correct the SIA throughout the volume of interest. Furthermore, most AO methods fail to accurately compensate for SIA in the presence of a strong MS background^1^. Therefore, there is generally trade between MS and SIA tolerance.

Recently, several label-free coherent imaging techniques have successfully addressed the detrimental effects by exploiting the optical and computational suppression of MS and SIA. The time-gated reflection matrix approach^9–11^, which contains all input-output responses at a certain depth, accurately corrected up to high-order aberrations without guide stars by insightfully identifying the aberrations hidden in the reflection matrix by post-processing. Another approach based on the incoherent synthesis of multi-angle OCT images with data-driven ray-trajectory correction^12,13^ successfully achieved high-resolution, speckle-free, and isotropic visualization. Benefitting from epi-detection configurations, these methods, including OCT, enable in vivo label-free observation at an unprecedented depth. However, epi-detection configurations may face difficulty in visualizing detailed biological structures, such as the morphology of individual cells and weak-backscattering subcellular structures, which reflect the architecture of the tissue and functionality of cells. Recently developed dynamic full-field OCT tackled this problem by performing temporal analysis on a series of FFOCT images, visualizing intracellular structures via intracellular motility^14,15^.

RI tomography, or holotomography, another technique based on the image contrast mechanism different from OCT, enables volumetric visualization of an RI distribution. It allows visualization of intracellular structures such as chromosomes, lipid droplets, mitochondria, and lysosomes^16–19^, benefiting from the RI being a biophysical parameter that reflects cellular properties. RI tomography retrieves RI distribution from a series of captured images under various illumination or detection conditions, such as angle-scanning illumination^16,20–23^ and through-focus imaging^23,24^, by solving the inverse scattering problem. The conventional reconstruction algorithm is based on a linear model that is derived from a weak scattering approximation (first Born or Rytov), providing excellent visualization of single cells; however, it is not valid for MS samples such as thick multicellular specimens. To overcome this limitation, recent studies have exploited more accurate forward propagation models, such as a multi-slice-based method^25–28^ and a series convolution approach of the Lippmann–Schwinger equation^29–31^. These nonlinear wave propagation models provide accurate RI distribution but have limited practical applicability due to their high computational costs. Recently, we proposed a multiple-scattering suppressive Rytov (MSS-Rytov) approach to realize efficient RI reconstruction of thick samples^32^. Unlike the conventional Rytov approximation, which applies first-order phase approximation to angle-scanned coherent fields and often suffers from severe phase distortion caused by MS samples, MSS-Rytov suppresses the MS by a numerical accumulation of the fields and applies the Rytov approximation to the MS-suppressed fields. Thus, it overcomes the limitation of the conventional Rytov approximation and a successful reconstruction of multicellular thick specimens is demonstrated. Additionally, in contrast to the non-linear approach, MSS-Rytov provides computationally efficient reconstruction due to the linearized model at the expense of reconstruction accuracy. Although the MSS-Rytov approach succeeded in visualizing subcellular structures inside multicellular spheroids, its imaging depth is still limited due to the tradeoff between MS and SIA suppression, restricting its application to more complicated biological specimens.

In this study, we propose a novel linear-model-based approach, called in-silico clearing, to significantly improve the imaging depth of RI tomography in comparison with conventional linear-model-based methods by simultaneously addressing MS and SIA. MS and SIA have the same origin, that is, the heterogeneity of the RI distribution. In-silico clearing RI tomography directly addresses this fundamental limitation. The proposed approach consists of partial reconstruction of RI distribution and wave-backpropagation through the partial RI map (Fig. 1c). First, the proposed approach partially reconstructs the RI distribution from angle-scanned complex fields within the range where the resolution is not degraded. After partial reconstruction, the complex fields are numerically backpropagated using a multi-slice-based method, which removes the wavefront distortions caused by the current partial RI map and allows unambiguous reconstruction of the next partial RI volume while suppressing MS and SIA. These operations are repeated till the top of the sample, realizing high-resolution RI reconstruction over an extended imaging depth. Each partial RI reconstruction is based on a linear model, and multi-slice-based propagation is performed only once for each partial volume, end to end, allowing computationally efficient reconstruction. Here, we first provide a detailed explanation of in-silico clearing RI tomography. Then, we computationally demonstrate its deep imaging capability and computational efficiency using a simulated multicellular spheroid. Subsequently, we experimentally demonstrate its superior deep imaging performance compared to that of conventional RI tomography and rapid, computationally efficient reconstruction using a liver spheroid of diameter 140 µm. Additionally, we demonstrate the generalization performance of the proposed method independent of sample morphology by visualizing a variety of cell-type spheroids. Subsequently, we estimate the lipid-droplet volume inside hepatocyte spheroids, demonstrating that the proposed RI tomography can at least semi-quantitatively assess RI-specific structures deep inside the specimens. Finally, we assess the morphological changes caused inside the spheroid by treatment with staurosporine (an apoptosis inducer) to verify whether the proposed RI tomography has sufficient resolving power to analyze subcellular morphological changes inside thick specimens and estimate biologically relevant information.

## Results

### Principle of partial reconstruction of refractive index distribution

Figure 1c shows a pipeline of the in-silico clearing approach. Generally, the resolution of optical microscopy decreases as the depth increases (Fig. 1a). The proposed approach first partially reconstructs the RI map within the range where the resolution is maintained and then removes the MS and SIA effects caused by the partial RI map using computational wave-backpropagation. The entire sample volume is split into several blocks in the z-direction and the aforementioned operations are repeated up to the top of the sample.

The experimental setup for in-silico clearing RI tomography involves a standard holographic tomography system, where the sample is illuminated with multiple incident angles and the diffracted light is recorded by an off-axis interferometer (Fig. 1b). The scattering potential *V*(***r***) of the sample is related to its RI distribution *n*(***r***) via $V\left( {{{\boldsymbol{r}}}} \right) = k_{{{\mathrm{b}}}}^2\left[ {\left( {n({{{\boldsymbol{r}}}})/n_{{{\mathrm{b}}}}} \right)^2 - 1} \right]$, where $k_{{{\mathrm{b}}}} = 2\pi n_{{{\mathrm{b}}}}/\lambda _0$ is the wavenumber in the medium, *λ*_0_ is the free-space wavelength, and *n*_b_ is the refractive index of the medium. The incident plane wave with incident wavevector ${{{\boldsymbol{k}}}}_{{{{\mathrm{in}}}}}^j$ ($j = 1, \ldots ,N_{{{{\mathrm{in}}}}}$, where *N*_in_ is the total number of incident angles) is denoted as $u_{{{{\mathrm{in}}}}}( {{{{\boldsymbol{r}}}},{{{\boldsymbol{k}}}}_{{{{\mathrm{in}}}}}^j}) = \exp( {{{{\mathrm{i}}}}{{{\boldsymbol{k}}}}_{{{{\mathrm{in}}}}}^j \cdot {{{\boldsymbol{r}}}}})$. When the wave illuminates the sample, the diffracted field $u(r,{{{\boldsymbol{k}}}}_{{{{\mathrm{in}}}}}^j)$ is given by the Lippmann–Schwinger equation as follows:

1 $$u( {{{{\boldsymbol{r}}}},{{{\boldsymbol{k}}}}_{{{{\mathrm{in}}}}}^j}) = u_{{{{\mathrm{in}}}}}( {{{{\boldsymbol{r}}}},{{{\boldsymbol{k}}}}_{{{{\mathrm{in}}}}}^j}) + {\int} {{{{\mathrm{d}}}}{{{\boldsymbol{r}}}}^\prime G\left( {{{{\boldsymbol{r}}}} - {{{\boldsymbol{r}}}}^\prime } \right)V\left( {{{{\boldsymbol{r}}}}^\prime } \right)u( {{{{\boldsymbol{r}}}}^\prime ,{{{\boldsymbol{k}}}}_{{{{\mathrm{in}}}}}^j})}$$

where *G*(***r***) is Green’s function of the Helmholtz equation.

First, we describe the reconstruction of the partial RI map from angle-scanned complex fields. For the partial reconstruction of an RI distribution, it is important to eliminate the crosstalk from outside the current block to the extent possible to avoid reconstruction errors. To circumvent crosstalk, the fields before reconstruction should be locally and quantitatively related to the RI map. In the conventional Rytov approximation, the RI map is reconstructed from the phase maps of the angle-scanned complex fields, where the phase map is called the quantitative phase imaging (QPI). However, using QPI images as pre-reconstructed fields hinders partial reconstruction owing to the undesired crosstalk caused by their heavy-tailed distribution in the depth direction. This heavy-tailed distribution is caused by the low illumination numerical aperture (NA) of each complex field. Traditionally, the optical sectioning in linear microscopes, such as DIC and confocal microscope, has been achieved by simultaneously increasing the illumination and detection NA. By contrast, each angle-scanned illumination comprises a single spatial frequency, resulting in the heavy-tailed distribution owing to the absence of the optical sectioning effect. A possible solution is to computationally synthesize the angle-scanned fields to increase the effective illumination NA. Fields can be synthesized in several ways, such as coherent^5,32^ or incoherent^32^ accumulation of fields, showing the optical sectioning effect (for a detailed explanation, see Supplementary Note 1). However, the coherent synthesis of fields is vulnerable to SIA^32^. Thus, we utilize the incoherent synthesis and employ quantitative phase gradient imaging (QPGI) images as a pre-reconstruction field. QPGI is known as a quantitative version of DIC^33,34^, showing the optical sectioning effect. QPGI images can be computationally simulated from angle-scanned complex fields using the following incoherent accumulation^32^:

2 $$\bar W\left( {{{\boldsymbol{r}}}} \right) = \frac{1}{{N_{{{{\mathrm{in}}}}}}}\mathop {\sum }\limits_j \bar u( {{{{\boldsymbol{r}}}},{{{\boldsymbol{k}}}}_{{{{\mathrm{in}}}}}^j} )\bar u^ \ast ( {{{{\boldsymbol{r}}}} + \delta {{{\boldsymbol{r}}}},{{{\boldsymbol{k}}}}_{{{{\mathrm{in}}}}}^j})$$

where $\bar u( {{{{\boldsymbol{r}}}},{{{\boldsymbol{k}}}}_{{{{\mathrm{in}}}}}^j}): = u( {{{{\boldsymbol{r}}}},{{{\boldsymbol{k}}}}_{{{{\mathrm{in}}}}}^j})\exp ( { - {{{\mathrm{i}}}}{{{\boldsymbol{k}}}}_{{{{\mathrm{in}}}}}^j \cdot {{{\boldsymbol{r}}}}})$, $\left\{ \cdot \right\}^ \ast$ is the complex conjugate operation and *δ****r*** is the shear vector of the QPGI. The QPGI is given by the phase of $\bar W\left( {{{\boldsymbol{r}}}} \right)$. The z-stacked QPGI images can be calculated using digital wave propagation for angle-scanned complex fields, followed by Eq. (2). Figure 1d shows QPI with normal incidence $\left( {\left( {\theta _x,\theta _y} \right) = \left( {0^ \circ ,0^ \circ } \right)} \right)$ and QPGI images calculated from diffracted fields measured for a homogeneous sphere. Here, we set the NA of the detection and illumination lenses to 1.0 and the diameter of the sphere to $2\lambda _0$. The QPI image exhibits a long-tailed distribution in the z-direction, which potentially causes undesired crosstalk. On the other hand, the QPGI image shows a short-tailed distribution owing to its optical sectioning capability. Therefore, QPGI images can potentially avoid crosstalk and realize partial RI reconstruction. To reconstruct an RI map from QPGI images, we need a quantitative relationship between the RI map and QPGI images. This relationship has been described as incoherent MSS-Rytov (iMSS-Rytov) in our previous study^32^, providing a theoretical relationship between an RI map and a QPGI image by applying Rytov approximation to incoherently accumulated fields (Eq. (2)) using perturbation theory. Using the convolution kernel derived from this theoretical relationship, the RI distribution was calculated by deconvolution of the z-stacked QPGI images. Therefore, applying iMSS-Rytov to a partial QPGI image allows partial reconstruction of the RI distribution (Fig. 1e). In this study, deconvolution is performed by iteratively solving a regularized optimization problem that uses a non-negative constraint and 3D total variation regularization. The detailed procedure and the pseudo-code for in-silico clearing RI tomography are provided in the Materials and methods section and Supplementary Notes 2 and 3.

### Wave-backpropagation using a partial RI map

Once the partial RI map is obtained, in-silico clearing RI tomography backpropagates the captured complex fields through the partial RI map to remove phase distortions caused by the RI map using the multi-slice-based method (Fig. 1f). To date, various slice-by-slice propagation methods have been proposed^25,27,28^. Among them, the beam propagation method (BPM) was used in this study owing to its computational efficiency and practically sufficient accuracy. The BPM comprises two operations: non-paraxial diffraction and phase distortion by an object. The operations can be represented as follows:

3 $$u( {{{{\boldsymbol{r}}}}_{{{\mathrm{t}}}},z + {{\Delta }}z;{{{\boldsymbol{k}}}}_{{{{\mathrm{in}}}}}^j} ) = O ( {{{{\boldsymbol{r}}}}_{{{\mathrm{t}}}},z + {{\Delta }}z;{{{\boldsymbol{k}}}}_{{{{\mathrm{in}}}}}^j} ){{{\mathcal{P}}}}_{{{\Delta }}z}\{ {u( {{{{\boldsymbol{r}}}}_{{{\mathrm{t}}}},z;{{{\boldsymbol{k}}}}_{{{{\mathrm{in}}}}}^j} )}\}$$

where ${{{\boldsymbol{r}}}}_{{{\mathrm{t}}}} = (x,y)$ is the transverse spatial coordinate, Δ*z* is the propagation distance, ${{{\mathcal{P}}}}_{{{\Delta }}z}\left\{ \cdot \right\} = {{{\mathcal{F}}}}^{ - 1}\{ {\exp ( {{{{\mathrm{i}}}}\sqrt {k_{{{\mathrm{b}}}}^2 - \left| {{{{\boldsymbol{k}}}}_{{{\mathrm{t}}}}} \right|^2} {{\Delta }}z} ){{{\mathcal{F}}}}\left( \cdot \right)} \}$ is the non-paraxial diffraction with a step size Δ*z*, ${{{\mathcal{F}}}}\left( \cdot \right)$ and ${{{\mathcal{F}}}}^{ - 1}\left( \cdot \right)$ are the 2D Fourier and inverse Fourier transforms, respectively, ${{{\boldsymbol{k}}}}_{{{\mathrm{t}}}} = (k_x,k_y)$ is the transverse wavenumber, $O( {{{{\boldsymbol{r}}}}_{{{\mathrm{t}}}},z;{{{\boldsymbol{k}}}}_{{{{\mathrm{in}}}}}^j} ) = {{{\mathrm{exp}}}}[ {{{{\mathrm{i}}}}k_0\delta n ( {{{{\boldsymbol{r}}}}_{{{\mathrm{t}}}},z} )\alpha ( {{{{\boldsymbol{k}}}}_{{{{\mathrm{in}}}}}^j}){{\Delta }}z}]$ is the phase distortion,　*k*_0_ and *k*_b_ are the free-space wavenumber and the wavenumber in the medium, $\delta n\left( {{{{\boldsymbol{r}}}}_{{{\mathrm{t}}}},z} \right) = n\left( {{{{\boldsymbol{r}}}}_{{{\mathrm{t}}}},z} \right) - n_{{{\mathrm{b}}}}$, and $\alpha ( {{{{\boldsymbol{k}}}}_{{{{\mathrm{in}}}}}^j} ) = 1/\cos \theta _{{{{\mathrm{in}}}}}^j = k_{{{\mathrm{b}}}}/\sqrt {k_{{{\mathrm{b}}}}^2 - | {{{{\boldsymbol{k}}}}_{{{{\mathrm{in}}}},\,{{{\mathrm{t}}}}}^j} |^2}$ is an obliquity factor. The obliquity factor is required to modify the amount of phase distortion for an oblique incidence^35^. In in-silico clearing RI tomography, the partially reconstructed RI map from the QPGI images is used in $\delta n\left( {{{{\boldsymbol{r}}}}_{{{\mathrm{t}}}},z} \right)$, and the BPM is executed in the backward direction using the captured complex fields as the initial fields:

4 $$u ( {{{{\boldsymbol{r}}}}_{{{\mathrm{t}}}},z - {{\Delta }}z;{{{\boldsymbol{k}}}}_{{{{\mathrm{in}}}}}^j} ) = O^ \ast ( {{{{\boldsymbol{r}}}}_{{{\mathrm{t}}}},z - {{\Delta }}z;{{{\boldsymbol{k}}}}_{{{{\mathrm{in}}}}}^j}){{{\mathcal{P}}}}_{ - {{\Delta }}z}\{ {u( {{{{\boldsymbol{r}}}}_{{{\mathrm{t}}}},z;{{{\boldsymbol{k}}}}_{{{{\mathrm{in}}}}}^j} )} \}$$

As both MS and SIA originate from the same physical phenomena, namely spatial variations in RI distribution, this backpropagation operation simultaneously mitigates them.

### Imaging a simulated multicellular spheroid

We applied in-silico clearing RI tomography to a simulated multicellular spheroid exhibiting MS and SIA and compared it to the case without in-silico clearing (equivalent to MSS-Rytov) to numerically demonstrate the deep imaging performance and computational efficiency of the proposed method. To analyze the performance of the reconstruction methods, we calculated the simulated measurements using the BPM as a forward model. To ensure the reliability of the simulation results, it was necessary to confirm the effects of the slice-by-slice approximation of the BPM on the accuracy of the simulated measurements. Therefore, we compared the accuracy of the two methods, BPM and the Lippmann–Schwinger equation, and confirmed that BPM has sufficient accuracy for simulating the measurements, as described in Supplementary Notes 4.I, 4.II. Moreover, to confirm the performance of BPM when used for backpropagation in in-silico clearing RI tomography, we confirmed the effect of the obliquity factor on the accuracy of wave-backpropagation in Supplementary Note 4.III. All processing in this study was performed using an NVIDIA RTX A6000 GPU and AMD Ryzen Threadripper 3990X CPU installed on a desktop computer.

The simulated spheroid has a diameter of 122*λ*_0_, and is within a volume of 672 × 672 × 336 voxels with a resolution of $\lambda _0/5 \times \lambda _0/5 \times 2\lambda _0/5$, comprising 331 cells with a diameter of 14*λ*_0_ arranged in a body-centered cubic lattice with a lattice constant of 18*λ*_0_. Each cell in the spheroid comprised cytoplasm (*n* = 1.370), a nucleus (*n* = 1.355), a nucleolus (*n* = 1.370), and several vesicles (*n* = 1.410), and the cells were randomly rotated. The sample was illuminated by 341 incident waves whose incident wavevectors were distributed in a regular grid pattern in k-space with a maximum illumination NA of 1.0. We set a detection NA to 1.0. We set the block length to *l*_b_ = 20*λ*_0_ in in-silico clearing RI tomography, splitting the entire volume into seven blocks.

**Fig. 2:**
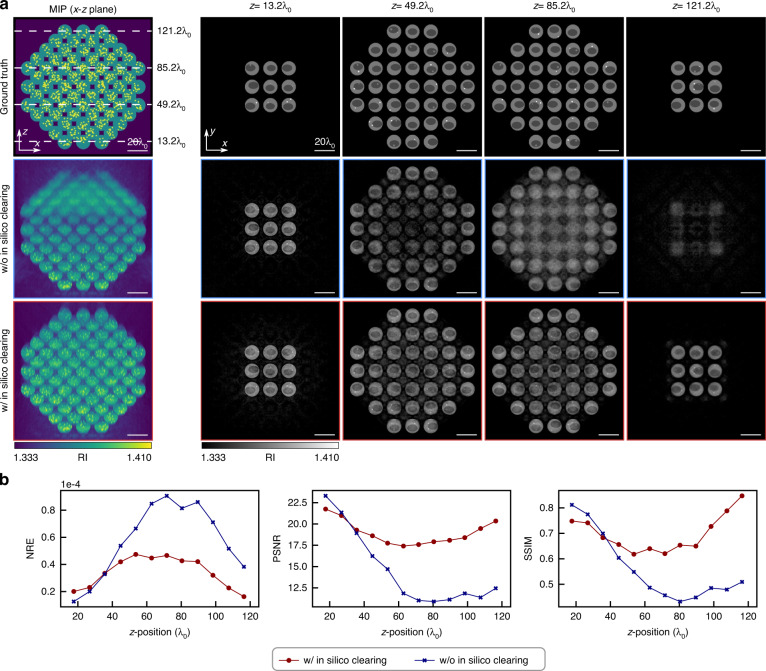
Numerical evaluation of the performance of in-silico clearing RI tomography using a simulated multicellular spheroid. **a** Maximum intensity projection (MIP) and cross-sections of RI distributions of ground truth, reconstructed without and with in-silico clearing, respectively. The cross-sections are identified with a white dashed line in MIP. **b** NRE, PSNR, and SSIM for without and with in-silico clearing as a function of depth. These metrics are computed in a layer-wise manner, where the layer thickness is set as lattice constant 18*λ*_0_

Figure 2a shows the maximum intensity projection (MIP) and *x*–*y* cross-section at $z = 13.2\lambda _0,\,49.2\lambda _0,\,85.2\lambda _0,\,121.2\lambda _0$ of the RI distributions of the ground truth, reconstructed RI distribution with and without in-silico clearing, respectively. The QPGI images calculated during the process with and without in-silico clearing are provided in Supplementary Note 5. Figure 2 shows that RI tomography without in-silico clearing failed to resolve fine structures at a depth of 49.2*λ*_0_ and was unable to produce images of sufficient resolution beyond this depth. In contrast, in-silico clearing RI tomography successfully resolved intracellular structures such as nuclei and nucleoli, even at a depth of 121.2*λ*_0_. To assess the performance of each method quantitatively, we calculated the normalized reconstruction error (NRE), peak signal-to-noise ratio (PSNR), and structural similarity index measure (SSIM) for the RI maps (see Materials and methods section for detailed definitions of the metrics). To quantify the reconstruction quality as a function of depth, we split the RI volumes into 18*λ*_0_ thick layers (which equals the lattice constant) and calculated the metrics for each layer. The metrics were then plotted as a function of the depth of the center of the layer (Fig. 2b). All three metrics showed that the case with in-silico clearing exhibited superior performance compared to the case without in-silico clearing at greater imaging depths. The total reconstruction times were 55 s and 124 s for cases without and with in-silico clearing, respectively. Although in-silico clearing RI tomography required 2.2 times longer processing times owing to the BPM operation, it demonstrated efficient RI reconstruction within a few minutes of processing. Additionally, we presented the in-process complex fields after propagating through each RI block in Supplementary Note 6, which shows that the phase of the complex fields gradually flattened and the single-to-multiple scattering ratio^36^ improved, thereby indicating that the proposed method correctly compensates for the wavefront distortion caused by the sample. Furthermore, we showed the RI distribution reconstructed by the conventional Rytov model in Supplementary Note 7; the reconstruction completely failed owing to severe phase distortion caused by MS, because the conventional Rytov approximation is only valid against a spatially slowly varying phase of the output field. Numerical simulations performed for the low NA and an absorbing sample, discussed in Supplementary Note 8 and 9, respectively, confirmed imaging depth improvement of in-silico clearing RI tomography compared to that of RI tomography without in-silico clearing. These results numerically confirm that the proposed approach enables deeper RI tomography compared to conventional linear-model-based methods in a computationally efficient manner even in the presence of MS and SIA.

### Imaging a live multicellular spheroid

To experimentally demonstrate the performance of in-silico clearing RI tomography, we observed a 3D-cultured human hepatoma HepG2 multicellular spheroid. 3D culture models are advantageous over traditional two-dimensional (2D) culture models as they can simulate the architecture and functionality of native tissues. However, the subcellular-resolution visualization of the entire sample by optical imaging is difficult due to MS and SIA. As a reference for visualization using other imaging modalities, we show the confocal and two-photon images of a HepG2 spheroid in Supplementary Note 13; notably, confocal microscopy lost resolution with an increase in depth and could not visualize the entire spheroid. In general, for high-quality imaging of a multicellular spheroid, optical clearing^37–40^ and an RI matching medium^41^ are used to mitigate the effects of MS and SIA, respectively. However, these methods are inseparable from invasive manipulations, chemical exposure, and labeling procedures. Therefore, it will be beneficial to visualize a 3D culture system in a noninvasive and label-free manner. It is known that 3D cultured hepatocyte more accurately represents their in vivo counterpart^42^ compared to 2D cultured hepatocyte. The liver plays a central role in metabolism with over 500 vital functionalities in vivo. Therefore, it is valuable for versatile applications related to liver function to visualize whole hepatocyte spheroids by overcoming MS and SIA.

We built an off-axis holographic RI tomography system equipped with a 2D scanning mirror and an HeNe laser (*λ*_0_ = 632.8 nm) as the light source. The sample was illuminated by plane waves with 333 different incident angles in a regular grid pattern in k-space with an illumination NA of 0.95. Each diffracted wave passed through a detection objective lens (×60/1.0 NA, water immersion) and was combined with a reference beam to generate an off-axis hologram on a CMOS camera. A series of images were recorded in 4.5 s. The captured raw images were converted to complex fields using digital holographic processing^43^. The detailed experimental setup, which was used for all experiments in this study, is described in Supplementary Note 12.

**Fig. 3:**
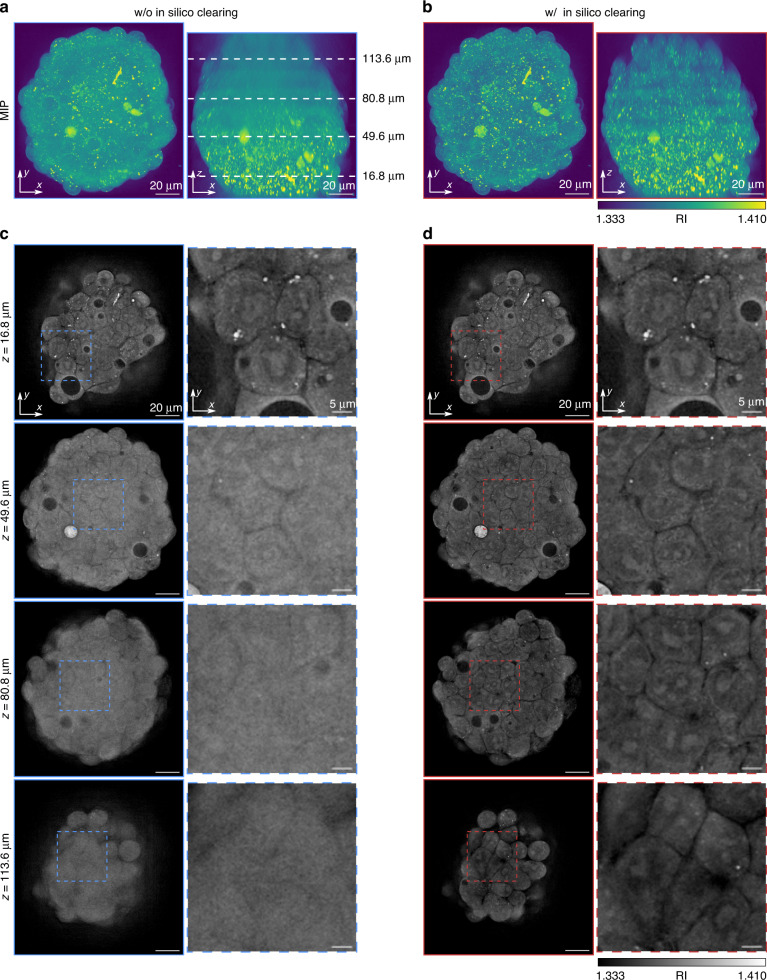
Experimental validation of in-silico clearing RI tomography using a human hepatocyte spheroid. Maximum intensity projection of the RI map reconstructed by (**a**) the case without in-silico clearing and (**b**) with in-silico clearing. **c, d** Cross-sections of the RI maps at different z locations (identified by white dashed lines in **a**). The movie showing all cross-sections is provided in Supplementary Video 1

Figure 3a and b shows the MIPs of the reconstructed RI distribution with and without in-silico clearing, respectively. The reconstruction volume contained 1038 × 1002 × 338 voxels with a voxel size of 0.138 × 0.138 × 0.400 μm^3^. In the process of in-silico clearing, we set the block length *l*_b_ = 15 μm and the thickness of the slice for wave-backpropagation as 0.4 µm, thereby dividing the entire volume into nine blocks. Here, the block length was empirically determined based on the observation of several multicellular spheroids. The same block length and voxel size were used for all experiments in this study. In the RI reconstruction without in-silico clearing, as the whole volume data were too large to reconstruct on GPU, we axially split the volume into nine blocks and stitched the RI maps together after reconstructing them individually. As seen in the MIPs of the *x*-*z* plane, in-silico clearing RI tomography succeeded in visualizing detailed structures that could not be resolved without in-silico clearing at large imaging depth (see Supplementary Video 1 showing all cross-sections). Figure 3c and d shows the four lateral slices for each reconstructed volume at depths of *z* = 16.8 µm, 49.6 µm, 80.8 µm, and 113.6 µm. Although the case without in-silico clearing produced a sharp resolution up to *z* = 16.8 µm, it began to lose its resolution at *z* = 49.6 µm and failed to resolve intercellular detailed structures and discriminate individual cells beyond *z* = 80.8 µm. However, the case with in-silico clearing produced a sharp resolution to discriminate individual cells and resolve intracellular structures, such as the nuclei and nucleoli. The total reconstruction times using our algorithm were 187 s and 386 s for the cases without and with in-silico clearing, respectively. Furthermore, we showed the RI distribution based on a reconstruction using the conventional Rytov model in Supplementary Note 14. However, the reconstruction failed owing to strong MS. Collectively, we have demonstrated the computational efficiency and improved deep imaging capability of the proposed method for a biological multicellular specimen compared to conventional linear-model-based techniques.

### Visualization of various cell-type spheroids

Subsequently, we demonstrate the generalization imaging performance of in-silico clearing RI tomography. Over the last few decades, 3D cultured cells have shown drastic phenotypic improvements across several cell types. As spheroids can mimic the architecture and functionality of native tissues, different cell-type spheroids represent their unique morphological complexity. To handle this diversity, the 3D imaging technique should provide consistent performance independent of sample morphology. To demonstrate the generalization performance of in-silico clearing RI tomography across different sample morphologies, we observed spheroids comprised of four different cell types: HepG2, A549 (human lung carcinoma), A172 (human glioblastoma), and F9 (mouse embryonal carcinoma).

**Fig. 4:**
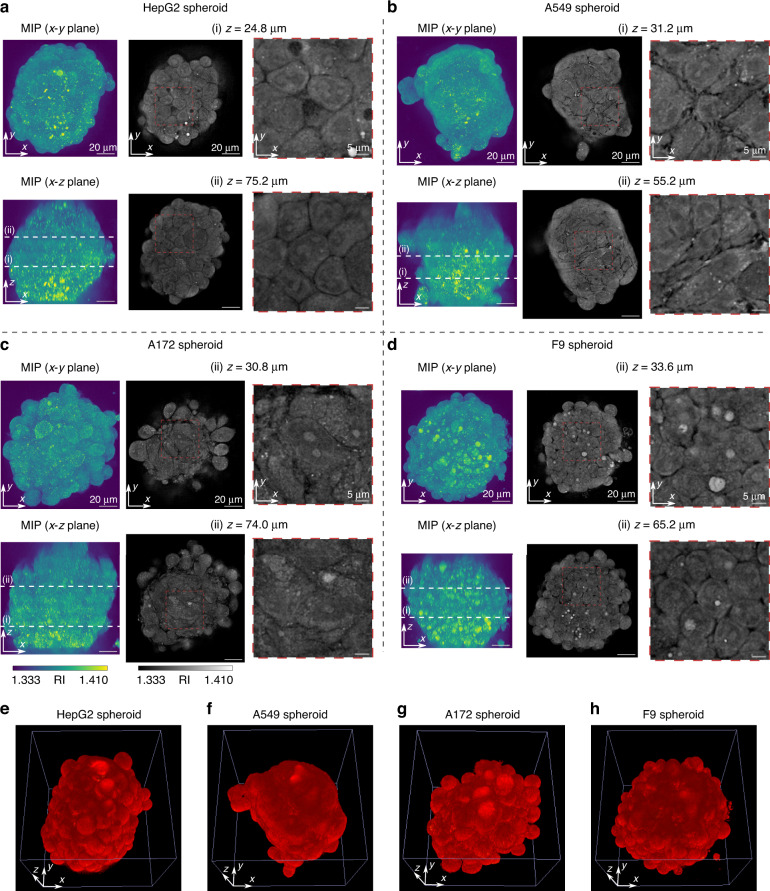
RI tomography of various cell-type spheroids by in-silico clearing RI tomography. Maximum intensity projection (MIP) and cross-sections of RI maps for (**a**) HepG2, (**b**) A549, (**c**) A172, and (**d**) F9 spheroids. The depth of the cross-sections is denoted by white dashed lines in the MIP images; 3D rendering by alpha blending of RI distributions for (**e**) HepG2, (**f**) A549, (**g**) A172, and (**h**) F9 spheroids. The movies showing all cross-sections are provided in Supplementary Videos 2 and 3

Figure 4a–d shows the MIPs and lateral cross-sections of RI distributions of HepG2, A549, A172, and F9 spheroids reconstructed by in-silico clearing RI tomography. Furthermore, Fig. 4e–h shows the 3D visualization rendered through alpha blending to illustrate their surface profiles (see Materials and methods for a detailed description of alpha blending). HepG2 (Fig. 4a) and A549 (Fig. 4b) spheroids comprised relatively round and anisotropically elongated cells, respectively. The A172 (Fig. 4c) spheroid showed cells having an anisotropically stretched shape; however, the surface of the spheroid had bumps and dips (Fig. 4g), which is different from the relatively smooth surface of the A549 spheroid (Fig. 4f). Although the F9 (Fig. 4d) spheroid had an individual cell morphology similar to that of the HepG2 spheroid, each cell was relatively small, thereby indicating that F9 spheroid has high cell density. For comparison, we also reconstructed the RI distribution without in-silico clearing and demonstrated that the entire spheroid could not be imaged with sufficient resolution, as shown in Supplementary Note 15. In conclusion, in-silico clearing RI tomography achieved consistent imaging performance that was independent of the spheroid morphology at least for the specimens observed in this experiment.

### Semi-quantification of high-RI structures inside hepatocyte spheroids

Further, to assess whether in-silico clearing RI tomography can quantify RI-specific structures, we performed lipid volume estimation inside HepG2 spheroids using the reconstructed RI distributions. Lipids are the fundamental components of all cells and play a key role in various processes and diseases^44^. Therefore, the visualization and quantification of lipids are valuable in the field of biomedicine. Owing to its high RI contrast structure, the visualization of lipid droplets is a promising application of RI tomography, as reported in previous studies^17,32^. Unfortunately, the localized high RI structure induces severe MS, hindering the visualization and quantification of detailed structures deep inside the samples. Accordingly, we assessed whether in-silico clearing RI tomography can estimate lipid volume even in the presence of severe MS.

**Fig. 5:**
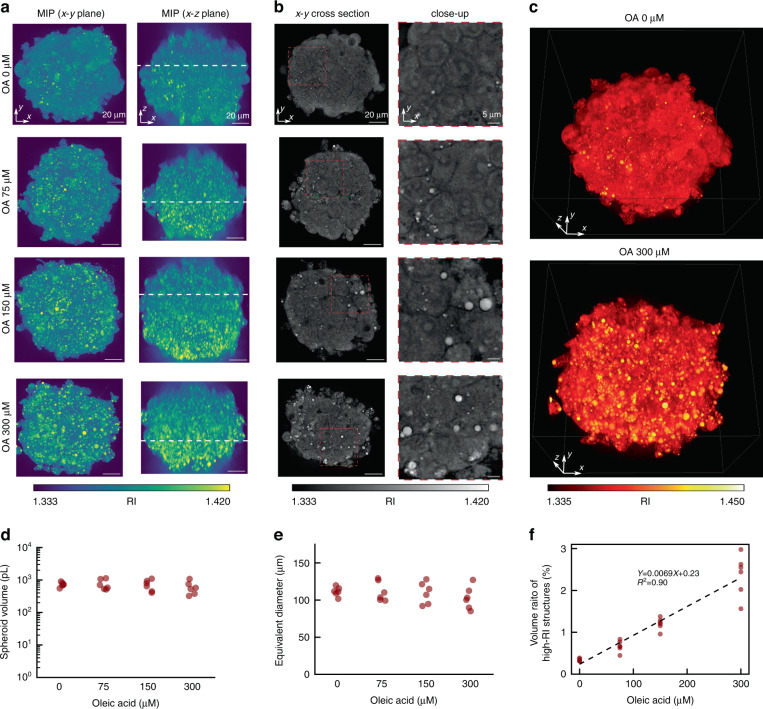
Semi-quantitative assessment of high-RI structures using in-silico clearing RI tomography. **a** Maximum intensity projection (MIP) and **b** cross-sections of the RI maps of HepG2 spheroids at oleic acid (OA) concentrations of 0, 75, 150, and 300 µM. The depth of cross-sections is identified with white dashed lines in MIP images. **c** 3D rendering of RI distribution at OA concentrations of 0 µM and 300 µM, respectively. Plots of (**d**) spheroid volume, (**e**) equivalent diameter, and (**f**) volume ratio of high-RI structures as a function of OA concentration. Six spheroids are measured for each OA concentration (a total of 24 spheroids). The fitted line in (**f**) has a slope of 0.0069%/µM and an intercept of 0.23% with a coefficient of determination *R*^2^ = 0.90

To induce lipid droplet production, HepG2 spheroids were exposed to different concentrations of oleic acid (OA): 0, 75, 150, and 300 µM. Existing studies demonstrated that lipid droplet formation by OA treatment is linearly correlated to OA concentration in 2D-cultured HepG2 cells^45^. Herein, we confirmed this relation in 3D-cultured hepatocytes using in-silico clearing RI tomography. We observed six spheroids at each OA concentration, resulting in a total of 24 spheroids. We showed the MIPs (Fig. 5a) and lateral cross-sections (Fig. 5b) of the RI distributions at each OA concentration. Furthermore, we rendered spheroids with concentrations of 0 and 300 µM for visualization in a 3D volume (Fig. 5c). The MIPs, lateral cross-sections, and 3D renderings of the HepG2 spheroids under various OA concentrations illustrated that the number of high-RI structures increased as OA concentration increased. To estimate the lipid volume inside the spheroids, we semi-quantitatively measured the morphological parameters and volume of the high-RI structures from the reconstructed RI distributions. We calculated the volume, equivalent diameter, and volume ratio of high-RI structures for each spheroid. The volume was calculated as the sum of regions where *n*(***r***) > 1.34, the equivalent diameter was calculated from the volume by assuming that the sample was an ideal sphere, and the volume ratio of high RI structures was calculated as the ratio of volumes where *n*(***r***) > 1.375 and *n*(***r***) > 1.34, as shown in Fig. 5d–f (see Materials and methods for detailed definition of parameters). The mean equivalent diameters (standard deviation) were 111.5 µm (6.1 µm), 111.5 µm (13.3 µm), 109.6 µm (14.4 µm), and 103.0 µm (15.3 µm) for the OA concentration of 0, 75, 150, and 300 µM, respectively; therefore, the sizes of the spheroids were almost equivalent. Figure 5f shows a high correlation coefficient between the volume ratio of high-RI structures and OA concentration, where the fitted line had a slope of 0.0069%/μM and an intercept of 0.23% with a coefficient of determination *R*^2^ = 0.90. This indicated that the lipid concentration estimated from RI distributions was highly correlated to the OA concentration. Taken together, these results suggested that in-silico clearing RI tomography had a semi-quantification capability to estimate biologically relevant events associated with RI-specific structures even in the presence of severe MS.

### Estimation of cell death inside spheroids

Finally, we demonstrate the ability of the proposed method to resolve subcellular morphological changes due to drug exposure inside the spheroids. As a representative drug, we chose staurosporine, a well-known apoptosis inducer. Apoptosis is genetically controlled cell death with characteristic morphological changes, such as cell shrinkage, nuclear segmentation, and fragmentation into membrane-bound apoptotic bodies. We treated HepG2 spheroids with different concentrations of staurosporine, observed subcellular morphological changes due to the drug exposure by in-silico clearing RI tomography, and semi-quantified the effect of the drug by analyzing reconstructed RI distributions.

**Fig. 6:**
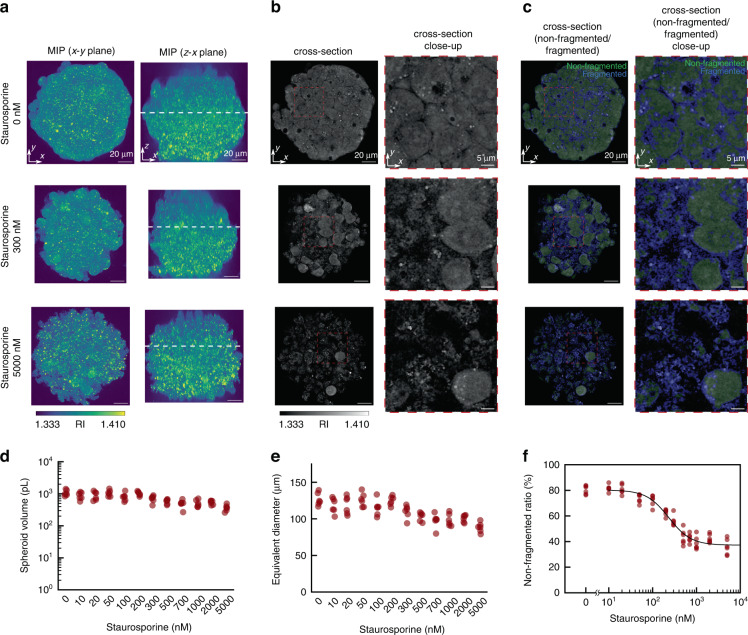
Semi-quantitative assessment of staurosporine-induced morphological changes in HepG2 spheroids using in-silico clearing RI tomography. **a** Maximum intensity projection (MIP), **b** cross-sections, and **c** cross-sections merged with non-fragmented/fragmented areas of RI maps of HepG2 spheroids at staurosporine concentrations of 0, 300, and 5000 nM, respectively. The cross-section is identified with white dashed lines in the MIP image. The movies showing all cross-sections are provided in Supplementary Movies 4–6. Plots of (**d**) spheroid volume, (**e**) equivalent diameter, and (**f**) non-fragmented ratio as a function of staurosporine concentration. Six spheroids were measured for each staurosporine concentration (a total of 72 spheroids). The black solid line in (**f**) denotes the fitted based on the Hill model. The relative 50% effective concentration *EC*_50_ = 233 nM and the Hill exponent *H* = 1.71

The HepG2 spheroids were treated with 12 different concentrations of staurosporine in the range of 0–5000 nM for 24 h. We confirmed that staurosporine treatment induced apoptotic cell death in HepG2 spheroids using fluorescent reagents Annexin V-FITC and propidium iodide (Supplementary Note 19). We observed and reconstructed six spheroids at each concentration. Therefore, a total of 72 spheroids were evaluated in this assay. Figure 6a, b shows the RI distributions of the HepG2 spheroids at staurosporine concentrations of 0, 300, and 5000 nM (refer to also Supplementary Video 4–6 for the entire visualization). In the control spheroids, live cells showed relatively smooth RI values and a clearly defined nuclear structure. In the staurosporine-treated spheroids with the highest concentration, the cells showed nucleus loss and subsequent fragmentation into small bodies, consistent with the morphological features of apoptosis. We plotted the spheroid volume and equivalent diameter, calculated similarly to Fig. 5, in Fig. 6d and e, respectively. As the first step toward the application of this method to drug efficacy estimation, semi-quantifying the morphological changes in the RI distributions due to staurosporine treatment is important. As shown in the cross-sections of the RI map, the cells in the control spheroids exhibited a relatively smooth distribution, whereas the cells in staurosporine-treated spheroids showed a distribution with a high spatial variation, suggesting cell body fragmentation. To exploit this morphological difference, we performed spatial filtering of the RI distribution using the fourth derivative of the Gaussian function to extract the fragmented regions. We defined the foreground of the cells as *n*(***r***) > 1.34. Further, we defined the fragmented region as the intersection of the foreground and the thresholded regions after applying a convolution of the fourth derivative of the Gaussian function with a standard deviation $\sigma _x,\sigma _y,\sigma _z = 138\,{{{\mathrm{nm}}}},\,138\,{{{\mathrm{nm}}}},\,{{{\mathrm{and}}}}\,400\,{{{\mathrm{nm}}}}$ to the RI distribution (see Supplementary Note 17 for a detailed explanation). The non-fragmented region was defined as the foreground minus the fragmented region. The cross-sections of the RI maps merged with the non-fragmented/fragmented regions are shown in Fig. 6c. The extracted fragmented regions overlapped well with the RI distribution showing characteristic morphological changes. Using these extracted morphological features, we semi-quantified the efficacy of staurosporine and plotted it as a function of staurosporine concentration, as shown in Fig. 6f. We defined the non-fragmented ratio of each spheroid as the ratio of the volume of the non-fragmented region to that of the foreground region. The plot was fitted using the Hill model^46^ (see Materials and methods section). The estimated relative 50% effective concentration *EC*_50_ and the Hill exponent *H* were *EC*_50_ = 233 nM and *H* = 1.71, respectively. This 50% effective concentration was consistent with a previously reported experimental validation, where *EC*_50_ was 410 nM for HepG2 spheroids treated with staurosporine for 48 h^47^, considering that our treatment was conducted for 24 h. Furthermore, we conducted the same assay for A549 spheroids to demonstrate the general applicability of the proposed method to spheroids of different cell-type (see Supplementary Note 18) and successfully semi-quantified drug efficacy for different cell types. Collectively, these experiments confirmed the capability of in-silico clearing RI tomography for the semi-quantitative analysis of sub-cellular morphological changes after staurosporine treatment.

## Discussion

In this study, we proposed the in-silico clearing approach to realize deep RI tomography overcoming MS and SIA and conducted a label-free evaluation of multicellular systems. We provided numerical and experimental demonstrations using a simulated spheroid (Fig. 2) and various multicellular spheroids (Figs. 3, 4), showing the improved deep imaging capability of the proposed method compared to other linear-model-based methods. To clearly demonstrate that our method suppresses MS and SIA, we independently assessed the MS and SIA tolerance using MS-dominant and SIA-dominant simulated samples and confirmed the superior performance compared to the case without in-silico clearing, as shown in Supplementary Notes 10, 11. These proof-of-principle demonstrations clearly support the generality of the proposed method to improve reconstruction performance for thick specimens compared to the conventional linear-model-based RI tomography techniques. In-silico clearing RI tomography provides deep imaging capability to the standard tomographic holography system without any hardware upgrades. Additionally, the linear MSS-Rytov model and the computationally efficient BPM make the proposed approach computationally efficient. Further, experiments estimating lipid droplet production (Fig. 5) and apoptotic morphological changes (Fig. 6) inside spheroids demonstrated the capability of the proposed method to conduct biological analyses of multicellular systems. Image processing used during these estimations was performed using primitive operations (thresholding and fourth derivative Gaussian convolution) on the RI distribution. This reflects the excellent biological interpretability of the RI distribution even for multicellular specimens. Furthermore, compared to fluorescence microscopy, the label-free visualization and non-invasiveness of the RI tomography circumvent the unexpected perturbation of critical biology owing to fluorescent staining or phototoxicity.

The in-silico clearing approach opens a new route towards deep optical imaging of thick specimens as it addresses the most fundamental cause of MS and SIA, namely inhomogeneity of an RI distribution, by employing partial RI reconstruction and wave-backpropagation. Unlike conventional methods that reject MS light to selectively detect single-scattering light, the proposed method exploits MS light to form an optical image. As for spatially varying SIA, in contrast to the in-silico clearing approach, most conventional approaches need to split the field of view into isoplanatic patches and compensate for SIA on a patch-by-patch basis. Furthermore, the in-silico clearing approach does not require invasive fixation or time-consuming incubation, as conducted in the chemical approach using the optical clearing method^37^.

To quantify the deep-imaging performance of the proposed method, we evaluated the imaging-depth limit in terms of the scattering mean free path *l*_S_, as shown in Supplementary Note 21. First, we developed a method to estimate *l*_S_ based on the angle-scanned complex fields as described in Supplementary Note 21.I and calculated *l*_S_ for various cell-type spheroids. It was determined that this parameter was 17.2 µm and 33.2 µm for A172 and HepG2 spheroids, respectively. This result indicates that A172 is more difficult to image than HepG2. We then observed large-sized spheroids and investigated the maximum imaging depth based on whether the nucleolus of these cells was resolved. This experiment quantified the maximum imaging depth with and without in-silico clearing as 6.78*l*_*S*_ and 1.94*l*_S_, respectively (see Supplementary Note 21 for a detailed description). This quantification of deep-imaging performance is not only crucial for evaluating the effectiveness of the proposed method, but is also helpful in providing a basis for comparison with future deep RI tomography methods in a unified framework using the scattering mean free path.

It is necessary to consider whether the imaging depth of in-silico clearing RI tomography is sufficient for various biological applications. The required imaging depth is highly dependent on its application. For example, the HepG2 spheroid showed liver-specific gene expression compared to the 2D culture, even at a diameter of ~100 µm^48^, similar size used in this study. In contrast, tumor spheroid models generally require a large diameter (>300 µm) to simulate hypoxic conditions. Therefore, exploring improvements to assess a wide range of complex biological systems is necessary.

There are several ways to improve the imaging-depth limit in the process of in-silico clearing RI tomography, which are associated with the underlying limitations of the technique. As the RI governs light-matter interactions, full characterization of the RI distribution leads to a complete description of wave propagation. In principle, there is no limit to the imaging depth that can be achieved using in-silico clearing, except when there are significant losses owing to absorption and undetected scattering, such as backscattering, provided that the partial RI reconstruction and wave-backpropagation are accurate. However, as confirmed by the experimental results shown in Fig. 5, the maximum value in the reconstructed RI tomogram of the samples decreases as the depth increases, thereby indicating a gradual decrease in resolution and thus insufficient accuracy of those operations (see Supplementary Note 6 for residual wavefront distortion due to insufficient accuracy). To increase the imaging-depth limit, the accuracy of the partial RI reconstruction and wave-backpropagation must be increased.

The lack of accuracy in the RI reconstruction can be attributed to the fact that the mathematical model derived in MSS-Rytov includes an approximation (modified Rytov approximation) to prioritize computational efficiency over reconstruction accuracy. Therefore, a more complex forward model, as used in nonlinear RI reconstruction methods^25,28–31^, can improve the accuracy if it can successfully incorporate the partial reconstruction approach. MSS-Rytov is also limited by the missing cone problem owing to its finite NA. The axial resolution can be improved by increasing the angular diversity, such as cell rotation^49,50^ and illumination angle scanning^13^. An alternative software solution is the regularization technique, such as an unsupervised machine learning approach that leverages the inherent regularization effect of convolutional neural networks^51^. Additionally, the partial reconstruction approach occasionally causes artifacts in the block boundary owing to the limit of the optical sectioning effect (see Supplementary Notes 8 and 11), which deteriorates the resulting accuracy. As the performance of optical sectioning also depends on the NA, the aforementioned solutions could suppress the artifact. In addition, block thickness is an important parameter for successfully imaging thick samples. If the block is too thick, reconstruction will be inaccurate in deep regions of the block. Conversely, if it is too thin, the computational complexity will increase. In this study, we empirically determined the block length, but it should be determined depending on the sample’s scattering strength. One possible solution is to determine the block length based on the scattering mean free path of the sample. In this case, the method for obtaining the scattering mean free path based on the angle-scanned fields as described in Supplementary Note 21 would be useful.

The lack of accuracy in wave-backpropagation is due to the simplicity of the BPM, which was adopted to prioritize computational efficiency over accuracy. In recent years, more accurate wavefront propagation methods have been proposed in the field of RI tomography^27,28^, which can be readily applied to the in-silico clearing approach.

We confirmed that in-silico clearing RI tomography has the potential to semi-quantitatively assess high-RI structure even in the presence of severe MS (Fig. 5f). This might be a key first step towards addressing the quantification of biologically relevant structures in spheroids. To this end, comparisons with conventional experiments (including fluorescence staining) are important in establishing biological assays based on the results in Fig. 5. In this study, we confirmed that the increment of high-RI structures with respect to OA concentration (Fig. 5f) is comparable with that of the lipid droplet volume ratio calculated from fluorescence observation (Fig. S18). In addition, the estimated volumes of high-RI structures (Fig. 5f) are in approximately the same order as in the lipid droplet volume ratio (Fig. S18). Although these results imply that our proposed method can estimate lipid droplet formation, they were semi-quantitative analyses. To establish quantitative assays, further improvements are essential in the future including accurate segmentation of subcellular structures by comparison with fluorescence images.

In-silico clearing RI tomography successfully characterized subcellular morphological changes induced by staurosporine treatment as shown in Fig. 6, suggesting the possibility of the proposed method to estimate apoptotic cell death. However, it has limitations when directly assessing apoptosis, as suggested by the fluorescence-based analyses (Fig. S21). Although few apoptotic cells remain at 0 nM of staurosporine (Fig. S21a, c), our method only detected ~80% of the spheroid volume as the non-fragmented region (estimated as a live cell region). Similarly, at 5000 nM of staurosporine, although most of the cells were apoptotic (Fig. S21b, d), only 60% of the spheroid volume was detected as a fragmented region (estimated as an apoptotic region). These data suggest that our method overestimates and underestimates cell death at 0 and 5000 nM, respectively. We also observed necrosis-like morphological change inside the HepG2 and A549 spheroids, as discussed in Supplementary Note 18, indicating a difference in the visualization of necrotic and apoptotic morphological changes. These results indicate that the proposed approach has potential applicability for viability assay in a 3D culture system. The next step will be accurate cell viability quantification inside the spheroids. To achieve this, more advanced techniques such as deep learning will be ideal, as seen in an existing study that performed a label-free real-time viability assessment in a 2D culture system with high accuracy using fluorescent images as the ground truth^52^. To take advantage of a data-driven approach using fluorescent images as the ground truth, combining RI tomography with 3D fluorescence microscopy, such as confocal or multi-photon microscopy, will be necessary. In this case, using wavefront compensation based on the RI distribution obtained by in-silico RI tomography, improving the imaging depth of 3D fluorescence microscopy without guide stars would be possible.

Another important step would be RI tomography operating in reflection mode. The proposed method requires a transmission setup, thereby compromising the opportunity for in vivo imaging. Therefore, a quest for deep RI tomography using an epi-detection configuration would be rewarding. Recently, scientists have already begun addressing this issue^53,54^.

Traditionally, optical imaging has elucidated various biological phenomena by observing two imaging modalities: holistic imaging by label-free microscopies, such as phase contrast and DIC microscopy, and molecular-specific imaging, predominantly by fluorescence microscopy. We believe that RI tomography can potentially play the former role in 3D imaging. Furthermore, in contrast to conventional label-free microscopy, RI tomograms have an outstanding advantage in their quantitativeness and biological relevance for the identification of some intracellular structures. Recently, Jo et al. ^55^ demonstrated that exploiting the quantitativeness and spatial distribution of RI maps enables label-free prediction of major endogenous intracellular structures using a deep learning-based model. In synergy with such an advanced technique, in-silico RI tomography could significantly facilitate system-level understanding of biological phenomena in 3D multicellular systems. In conclusion, we envisage that our method will provide unprecedented information on complex biological specimens with minimal invasiveness and find wide application in the evaluation of multicellular specimens.

## Materials and methods

### Reconstruction of a refractive index distribution

In the in-silico clearing approach, RI maps are reconstructed using x- and y-sheared QPGI images as pre-reconstruction fields. This method was formulated in our previous study^32^ as iMSS-Rytov. However, in contrast to our previous study, we additionally imposed a total variation regularization^56^ in this study. Let ${{{\boldsymbol{\psi }}}}_x,\,{{{\boldsymbol{\psi }}}}_y$ be the vector representations of the x-, y-sheared QPGI images. To reconstruct the RI distribution ***x****,* we solve the following inverse problem:

5 $$\mathop {{\min }}\limits_{{{{\boldsymbol{x}}}} \ge 0} \frac{1}{2}\left\| {{{{\mathrm{CG}}}}_{{{{\mathrm{d}}}},x}{{{\boldsymbol{x}}}} - {{{\boldsymbol{\psi }}}}_x} \right\|_2^2 + \frac{1}{2}\left\| {{{{\mathrm{CG}}}}_{{{{\mathrm{d}}}},y}{{{\boldsymbol{x}}}} - {{{\boldsymbol{\psi }}}}_y} \right\|_2^2 + \tau \left\| {{{\boldsymbol{x}}}} \right\|_1 + \xi \left\| {{\Psi }{{{\boldsymbol{x}}}}} \right\|_1$$

where ${{{\mathrm{G}}}}_{{{{\mathrm{d}}}},x}$ and ${{{\mathrm{G}}}}_{{{{\mathrm{d}}}},y}$ are the discretized derivatives of 3D Green’s function with respect to the x- and y-directions, respectively. *C* is the cropping operator, Ψ is the finite difference operator, and *τ*, *ξ* are the tuning parameters. Unless otherwise specifically noted, we used $\tau = 7.5 \times 10^{ - 2}$, *ξ* = 0.1. We solved Eq. (5) using the alternating direction method of multipliers (ADMM) and executed 50 iterations of ADMM. Both RI tomography with/without in-silico clearing used Eq. (5) for the RI reconstruction. The update rule for the ADMM is described in detail in Supplementary Note 2.

### Image quality metrics

Let $n_{{{{\mathrm{exact}}}}}\left( {{{\boldsymbol{r}}}} \right)$ be the ground truth of a RI distribution and $n_{{{{\mathrm{recon}}}}}\left( {{{\boldsymbol{r}}}} \right)$ be the reconstructed RI distribution. We define NRE as

6 $$NRE = \frac{{\Vert n_{{{{\mathrm{recon}}}}}\left( {{{\boldsymbol{r}}}} \right) - n_{{{{\mathrm{exact}}}}}\left( {{{\boldsymbol{r}}}} \right)\Vert_2^2}}{{\Vert n_{{{{\mathrm{exact}}}}}\left( {{{\boldsymbol{r}}}} \right)\Vert_2^2}}$$

We also define PSNR as

7 $$PSNR = 10\log _{10}\frac{{L^2}}{{{{{\mathrm{MSE}}}}(n_{{{{\mathrm{recon}}}}}\left( {{{\boldsymbol{r}}}} \right),n_{{{{\mathrm{exact}}}}}\left( {{{\boldsymbol{r}}}} \right))}}$$

where $L = \max \left( {n_{{{{\mathrm{exact}}}}}\left( {{{\boldsymbol{r}}}} \right)} \right) - \min \left( {n_{{{{\mathrm{exact}}}}}\left( {{{\boldsymbol{r}}}} \right)} \right),{{{\mathrm{and}}}}\,{{{\mathrm{max}}}}\left( \ast \right)$ and $\min \left( \ast \right)$ return the maximum and minimum values of the argument, respectively, and ${{{\mathrm{MSE}}}}( \ast , \ast )$ returns the mean squared error of the arguments. The SSIM between an image *f,g* is defined as

8 $$SSIM = \frac{{2\mu _f\mu _g + C_1}}{{\mu _f^2 + \mu _g^2 + C_1}}\frac{{2\sigma _{fg} + C_2}}{{\sigma _f^2 + \sigma _g^2 + C_2}}$$

where $\mu _f\,{{{\mathrm{and}}}}\,\mu _g$ are the averages of $f,\,g,\,{{{\mathrm{respectively}}}},\,{{{\mathrm{and}}}}\,\sigma _f\,{{{\mathrm{and}}}}\,\sigma _g$ are the standard deviations of *f* and *g*, respectively, and $\sigma _{fg}$ is the covariance between *f* and *g*, where these statistical parameters are calculated within a small window. We set $C_1 = (0.01L)^2,\,C_2 = \left( {0.03L} \right)^2$ and the window size to 7 × 7 × 7.

### Volume rendering

In alpha blended rendering, volume data is sampled in the direction toward the viewpoint along a ray trajectory, and the value of the volume data is superimposed in sequence by using opacity for weighting. Consequently, objects close to the viewpoint are highlighted. We performed all 3D visualizations in this study using Vaa3D^57^.

### Morphological and biochemical parameters

The volume of a spheroid *V*_sph_ was calculated by integrating all voxels of the RI map with *n*(***r***) > 1.340. The equivalent diameter *d*_eq_ was calculated from the volume *V*_sph_ assuming that the shape of the object was spherical: $d_{{{{\mathrm{eq}}}}} = 2\left( {3V_{{{{\mathrm{sph}}}}}/4\pi } \right)^{\frac{1}{3}}$. The volume ratio of high-structure $C_{{{{\mathrm{high}}}}}$ is defined as the ratio of the lipid volume $V_{{{{\mathrm{high}}}}}$ to the spheroid volume $V_{{{{\mathrm{sph}}}}}$, where $V_{{{{\mathrm{high}}}}}$ is calculated by integrating all voxels in the region *n(****r****)* > 1.375.

### Sample preparation

Multicellular spheroids were formed using a 3D cell culture container, EZSPHERE 6-well plate (AGC Techno Glass) in 2 mL of the standard culture medium as described in Supplementary Note 22.

For the semi-quantitative analysis of high-RI structures, HepG2 spheroids were further cultured for 2 days in phenol-red-free Dulbecco’s modified Eagle’s medium (DMEM, Gibco) containing 1% (w/v) fatty-acid-free bovine serum albumin (BSA, FUJIFILM Wako Pure Chemical) and different concentrations of sodium oleate (Nacalai Tesque) between 0 and 300 µM. The spheroids were then washed in Hanks’ balanced salt solution (Gibco) and collected via centrifugation at 190 g for 3 min.

The HepG2 and A549 spheroids for apoptotic-like cell death analysis were exposed to various concentrations of staurosporine (FUJIFILM Wako Pure Chemical) for one day after spheroid formation.

### Fitting dose–response curve

We fitted the dose-response curve for staurosporine treatment based on the Hill model^46^, which describes the effect obtained at a given concentration *C* using the following equation:

9 $$E_{{{{\mathrm{Hill}}}}}\left( {C,\,E_\infty ,\,E_0,EC_{50},\,H} \right) = E_0 + \frac{{E_\infty - E_0}}{{1 + \left( {EC_{50}/C} \right)^H}}$$

where *EC*_50_ is the relative 50% effective concentration, *H* is the Hill exponent, *E*_∞_ is the maximum effect, and *E*_0_ is the effect without a drug.

## Supplementary information

Supplementary Note

Supplementary Video 1

Supplementary Video 2

Supplementary Video 3

Supplementary Video 4

Supplementary Video 5

Supplementary Video 6

## Data Availability

The study data are available from the corresponding author upon request.
