# Supplementary material for: In-silico clearing approach for deep refractive index tomography by partial reconstruction and wave-backpropagation
Source: Light Sci Appl. 2023 Apr 27;12:101. doi: 10.1038/s41377-023-01144-z (PMC10140380; doi:10.1038/s41377-023-01144-z)
Supplement: Supplementary file 1 — Supplementary Note [file 41377_2023_1144_MOESM1_ESM.pdf]

SUPPLEMENTARY NOTE

In-silico clearing approach for deep refractive index tomography by partial reconstruction and wave-backpropagation

Osamu Yasuhiko[1*], Kozo Takeuchi[1*]

[1]Central Research Laboratory, Hamamatsu Photonics K.K., 5000 Hirakuchi, Hamakita-ku, Hamamatsu, Shizuoka 434-8601, Japan

[*]osamu.yasuhiko@crl.hpk.co.jp, kozo.takeuchi@crl.hpk.co.jp

Content

1. Optical sectioning capability 2
2. Update rule of ADMM 2
3. Pseudo code of in-silico clearing RI tomography 4
4. Accuracy of BPM 6
   I. Effect of z step size and obliquity factor of beam propagation method on forward model accuracy 6
   II. Comparison of 3D RI reconstruction accuracy 8
   III. Wave-backpropagation accuracy of BPM 8
5. QPGI images of a simulated multicellular spheroid with and without in-silico clearing 10
6. Performance of the removal of the wavefront distortion 10
7. Conventional Rytov reconstruction of a simulated multicellular spheroid 11
8. Low numerical aperture simulation using a simulated spheroid 12
9. RI reconstruction of an absorbing simulated spheroid 16
10. Imaging multiple-scattering simulated sample 18
11. Imaging sample-induced-aberration simulated sample 19
12. Experimental setup 20
13. Confocal and two-photon imaging of a HepG2 spheroid 21
14. Conventional Rytov reconstruction of a HepG2 spheroid 22
15. RI tomography of different cell-type spheroids without in-silico clearing 23
16. Confocal imaging of lipid droplet 24
17. Discrimination of non-fragmented/fragmented regions in the spheroid 25
18. Observation of staurosporine-induced morphological changes inside A549 spheroids 28
19. Confocal imaging of staurosporine-treated spheroids 29
20. Observation of necrosis-like morphology inside HepG2 and A549 spheroids 31
21. Imaging-depth limit of the proposed method 32
   I. Estimation of scattering mean free path 32
   II. Characterization of the imaging-depth limit of the proposed method 33
22. Cell culture 37

1. Optical sectioning capability

In this section, we explain the optical sectioning capability of the coherent and incoherent accumulation of fields. The computational coherent accumulation of fields can be realized by the following equation: $\sum_j \bar{u}(\boldsymbol{r}; \boldsymbol{k}_{\text{in}}^j)$. This operation was introduced to selectively extract single-scattered light[1]. Therefore, the phase of the coherently accumulated field can be considered a confocal QPI image, showing optical sectioning capability (in the middle column of Fig. S1). The drawback of this approach is the vulnerability to sample-induced aberration as described in our previous paper[2]. Therefore, this approach is not suitable for visualizing thick samples. The computational incoherent accumulation of fields can be achieved by summing up fields irrespective of their absolute phases, as observed in QPGI (Eq. (2). The QPGI image in Fig. S1 shows optical sectioning capability owing to an increase of illumination numerical aperture by the incoherent synthesis of fields.

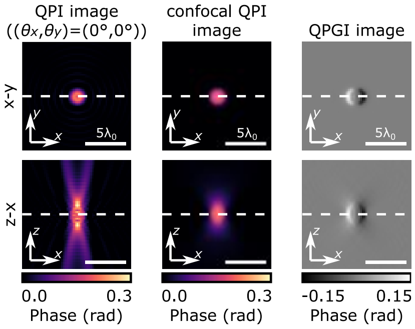

Fig. S1 Optical sectioning effect of the various fields. The diffracted fields are calculated for a homogeneous sphere. NAs of the detection and illumination lenses are set to 1.0, and the diameter of the sphere is set to $2\lambda_0$.

2. Update rule of ADMM

Herein, we describe the ADMM update rule in Eqs. (5) by transforming Eq. (5) into the constrained problem using variable splitting:

$$\min_{\boldsymbol{y},\boldsymbol{z},\boldsymbol{u},\boldsymbol{v},\boldsymbol{w}} \frac{1}{2}\|\mathrm{C}\boldsymbol{y}-\boldsymbol{\psi}_x\|_2^2 + \frac{1}{2}\left\|\mathrm{C}\boldsymbol{z}-\boldsymbol{\psi}_y\right\|_2^2 + \tau\|\boldsymbol{u}\|_1 + 1_+(\boldsymbol{v}) + \xi\|\boldsymbol{w}\|_1$$

$$\text{s.t.}\ \boldsymbol{y} = \mathrm{G}_{\mathrm{d},x}\boldsymbol{x}$$
$$\boldsymbol{z} = \mathrm{G}_{\mathrm{d},y}\boldsymbol{x}$$
$$\boldsymbol{u} = \boldsymbol{x}$$
$$\boldsymbol{v} = \boldsymbol{x}$$
$$\boldsymbol{w} = \Psi\boldsymbol{x}, \tag{S1}$$

where $\boldsymbol{y},\boldsymbol{z},\boldsymbol{u},\boldsymbol{v},$ and $\boldsymbol{w}$ are the auxiliary variables, and 1_+ is a barrier function, defined as $1_+(x) \coloneqq 0$ if $x \geq 0,$ and $+\infty$ otherwise. Next, we transform Eq. (S1) into an unconstrained augmented Lagrangian form, given as:

$$\min_{\boldsymbol{p},\boldsymbol{q},\boldsymbol{r},\boldsymbol{s},\boldsymbol{t}} \Bigg[\min_{\boldsymbol{x},\boldsymbol{y},\boldsymbol{z},\boldsymbol{u},\boldsymbol{v},\boldsymbol{w}} \frac{1}{2}\|\mathrm{C}\boldsymbol{y}-\boldsymbol{\psi}_x\|_2^2 + \frac{\mu_1}{2}\left\|\boldsymbol{y}-\mathrm{G}_{\mathrm{d},x}\boldsymbol{x}+\boldsymbol{p}\right\|_2^2$$

$$+\frac{1}{2}\left\|\mathrm{C}\boldsymbol{z}-\boldsymbol{\psi}_y\right\|_2^2 + \frac{\mu_1}{2}\left\|\boldsymbol{z}-\mathrm{G}_{\mathrm{d},y}\boldsymbol{x}+\boldsymbol{q}\right\|_2^2$$

$$+\tau\|\boldsymbol{u}\|_1 + \frac{\mu_2}{2}\|\boldsymbol{u}-\boldsymbol{x}+\boldsymbol{r}\|_2^2$$

$$+1_+(\boldsymbol{v}) + \frac{\mu_3}{2}\|\boldsymbol{v}-\boldsymbol{x}+\boldsymbol{s}\|_2^2$$

$$+\xi\|\mathbf{w}\|_1+\frac{\mu_4}{2}\|\boldsymbol{w}-\Psi\boldsymbol{x}+\boldsymbol{t}\|_2^2\Big],\tag{S2}$$

where $\boldsymbol{p},\boldsymbol{q},\boldsymbol{r},\boldsymbol{s},$ and $\boldsymbol{t}$ are dual variables associated with $\boldsymbol{y},\boldsymbol{z},\boldsymbol{u},\boldsymbol{v},$ and $\boldsymbol{w}$, respectively. $\mu_1,\mu_2,\mu_3,$ and μ_4 are the augmented Lagrangian parameters, where we use the same parameters for the augmented Lagrangian parameters associated with $\boldsymbol{y}$ and $\boldsymbol{z}$ as μ_1 because these terms are only different in the shear direction. Then, we updated the primal variables independently using the most recent value of the other primal variable and the dual variable:

$$\boldsymbol{y}^{k+1}\leftarrow \mathrm{argmin}_{\boldsymbol{y}}\ \frac{1}{2}\|\mathrm{C}\boldsymbol{y}-\boldsymbol{\psi}_x\|_2^2+\frac{\mu_1}{2}\left\|\boldsymbol{y}-\mathrm{G}_{\mathrm{d},x}\boldsymbol{x}^k+\boldsymbol{p}^k\right\|_2^2$$

$$\boldsymbol{z}^{k+1}\leftarrow \mathrm{argmin}_{\boldsymbol{z}}\ \frac{1}{2}\left\|\mathrm{C}\boldsymbol{z}-\boldsymbol{\psi}_y\right\|_2^2+\frac{\mu_1}{2}\left\|\boldsymbol{z}-\mathrm{G}_{\mathrm{d},y}\boldsymbol{x}^k+\boldsymbol{q}^k\right\|_2^2$$

$$\boldsymbol{u}^{k+1}\leftarrow \mathrm{argmin}_{\boldsymbol{u}}\ \tau\|\boldsymbol{u}\|_1+\frac{\mu_2}{2}\|\boldsymbol{u}-\boldsymbol{x}^k+\boldsymbol{r}^k\|_2^2$$

$$\boldsymbol{v}^{k+1}\leftarrow \mathrm{argmin}_{\boldsymbol{v}}\ 1_+(\boldsymbol{v})+\frac{\mu_3}{2}\|\boldsymbol{v}-\boldsymbol{x}^k+\boldsymbol{s}^k\|_2^2$$

$$\boldsymbol{w}^{k+1}\leftarrow \mathrm{argmin}_{\boldsymbol{w}}\ \xi\|\mathbf{w}\|_1+\frac{\mu_4}{2}\|\boldsymbol{w}-\Psi\boldsymbol{x}^k+\boldsymbol{t}^k\|_2^2$$

$$\boldsymbol{x}^{k+1}\leftarrow \mathrm{argmin}_{\boldsymbol{x}}\ \frac{\mu_1}{2}\left\|\boldsymbol{y}^{k+1}-\mathrm{G}_{\mathrm{d},x}\boldsymbol{x}+\boldsymbol{p}^k\right\|_2^2+\frac{\mu_1}{2}\left\|\boldsymbol{z}^{k+1}-\mathrm{G}_{\mathrm{d},y}\boldsymbol{x}+\boldsymbol{q}^k\right\|_2^2$$

$$+\frac{\mu_2}{2}\|\boldsymbol{u}^{k+1}-\boldsymbol{x}+\boldsymbol{r}^k\|_2^2+\frac{\mu_3}{2}\|\boldsymbol{v}^{k+1}-\boldsymbol{x}+\boldsymbol{s}^k\|_2^2+\frac{\mu_4}{2}\|\boldsymbol{w}^{k+1}-\Psi\boldsymbol{x}+\boldsymbol{t}^k\|_2^2.\tag{S3}$$

The solutions of Eq. (S3) are

$$\boldsymbol{y}^{k+1}\leftarrow\frac{1}{\mathrm{C}^{\mathrm{T}}\mathrm{C}+\mu_1}\left[\mathrm{C}^{\mathrm{T}}\boldsymbol{\psi}_x+\mu_1\left(\mathrm{G}_{\mathrm{d},x}\boldsymbol{x}^k-\boldsymbol{p}^k\right)\right]$$

$$\boldsymbol{z}^{k+1}\leftarrow\frac{1}{\mathrm{C}^{\mathrm{T}}\mathrm{C}+\mu_1}\left[\mathrm{C}^{\mathrm{T}}\boldsymbol{\psi}_y+\mu_1\left(\mathrm{G}_{\mathrm{d},y}\boldsymbol{x}^k-\boldsymbol{q}^k\right)\right]$$

$$\boldsymbol{u}^{k+1}\leftarrow S_{\frac{\tau}{\mu_2}}(\boldsymbol{x}^k-\boldsymbol{r}^k)$$

$$\boldsymbol{v}^{k+1}\leftarrow \max\left(\boldsymbol{x}^k-\boldsymbol{s}^k,0\right)$$

$$\boldsymbol{w}^{k+1}\leftarrow S_{\frac{\xi}{\mu_4}}(\Psi\boldsymbol{x}^k-\boldsymbol{t}^k)$$

$$\boldsymbol{x}^{k+1}\leftarrow\frac{1}{\mu_1\mathrm{G}_{\mathrm{d},x}^{\mathrm{T}}\mathrm{G}_{\mathrm{d},x}+\mu_1\mathrm{G}_{\mathrm{d},y}^{\mathrm{T}}\mathrm{G}_{\mathrm{d},y}+\mu_2+\mu_3+\mu_4\Psi^{\mathrm{T}}\Psi}\left[\mu_1\mathrm{G}_{\mathrm{d},x}^{\mathrm{T}}(\boldsymbol{y}^{k+1}+\boldsymbol{p}^k)+\mu_1\mathrm{G}_{\mathrm{d},y}^{\mathrm{T}}(\boldsymbol{z}^{k+1}+\boldsymbol{q}^k)\right.$$

$$\left.+\mu_2(\boldsymbol{u}^{k+1}+\boldsymbol{r}^k)+\mu_3(\boldsymbol{v}^{k+1}+\boldsymbol{s}^k)+\mu_4\Psi^{\mathrm{T}}(\boldsymbol{w}^{k+1}+\boldsymbol{t}^k)\right]\tag{S4}$$

where the soft thresholding operator S is defined as

$$S_\eta(x)=\begin{cases}x-\eta, & x\geq\eta\\ 0, & -\eta\leq x\leq\eta\\ x+\eta, & x\leq-\eta.\end{cases}$$

The dual variables are updated as follows:

$$\boldsymbol{p}^{k+1}\leftarrow\boldsymbol{p}^k+\boldsymbol{y}^{k+1}-\mathrm{G}_{\mathrm{d},x}\boldsymbol{x}^{k+1}$$
$$\boldsymbol{q}^{k+1}\leftarrow\boldsymbol{q}^k+\boldsymbol{z}^{k+1}-\mathrm{G}_{\mathrm{d},y}\boldsymbol{x}^{k+1}$$
$$\boldsymbol{r}^{k+1}\leftarrow\boldsymbol{r}^k+\boldsymbol{u}^{k+1}-\boldsymbol{x}^{k+1}$$

$$\boldsymbol{s}^{k+1} \leftarrow \boldsymbol{s}^{k} + \boldsymbol{v}^{k+1} - \boldsymbol{x}^{k+1}$$
$$\boldsymbol{t}^{k+1} \leftarrow \boldsymbol{t}^{k} + \boldsymbol{w}^{k+1} - \Psi\boldsymbol{x}^{k+1}. \quad \text{(S5)}$$

Repeating the update given by Eqs. (S4), (S5) yields the reconstruction of the RI distribution. Unless otherwise specifically noted, we used $\mu_1 = 1, \mu_2 = 10, \mu_3 = 7,$ and $\mu_4 = 0.1$ in this study.

3. Pseudo code of in-silico clearing RI tomography

We explain the implementation of in-silico clearing RI tomography. We show the pseudocodes for BPM and in-silico clearing RI tomography in Pseudocodes S1 and S2, respectively. As it is important to suppress crosstalk from behind the current block, we leveraged the optical sectioning capability of the QPGI to reduce crosstalk. However, imperfections in the optical sectioning effect prevent crosstalk suppression. To address this problem, when reconstructing a partial RI map in in-silico clearing RI tomography, we additionally reconstructed N_{pad} slices and overlapped the neighboring blocks to circumvent undesired crosstalk. When patching the adjacent blocks, we applied a Tukey window (with a shape parameter of 0.75) to each block to smoothly connect the partial RI blocks. We used $N_{\mathrm{pad}} = 20$ voxels $(= 8\lambda_0)$ and 20 voxels $(= 8\ \mu\mathrm{m})$ for the simulations and experiments, respectively.

Pseudocode S1. Beam propagation method

<u>Input</u>

$\{u^j(\boldsymbol{\rho})\}_{j=1}^{N_{\text{in}}}$: Complex fields at position z=z_{ini}, where $\boldsymbol{\rho}=(x,y)$ and N_{in} is the total number of incident angles

$\{\boldsymbol{k}_{\text{in}}^j\}_{j=1}^{N_{\text{in}}}$: 3D incident wavevectors of $\{u^j(\boldsymbol{\rho})\}_{j=1}^{N_{\text{in}}}$

$\{\delta n(\boldsymbol{\rho}, z_{\text{ini}}+m\Delta z)\}_{m=1}^{N_{\text{step}}}$: refractive index contrast, where Δz is the step size in the z-direction

Δz: step size in the z-direction

k_{b}: wavenumber in the medium

k_0: wavenumber in the vacuum

<u>Initialization</u>

$\{u_{\text{prop}}^j(\boldsymbol{\rho})\}_{j=1}^{N_{\text{in}}} = \{u^j(\boldsymbol{\rho})\}_{j=1}^{N_{\text{in}}}$

1. **for** $j \leftarrow 1\ to\ N_{\text{in}}$ **do**
2. $\alpha(\boldsymbol{k}_{\text{in}}^j) \leftarrow k_{\text{b}}/\sqrt{k_{\text{b}}^2 - k_{\text{in},x}^{j\,2} - k_{\text{in},y}^{j\,2}}$ $\quad((k_{\text{in},x}^j, k_{\text{in},y}^j, k_{\text{in},z}^j) = \boldsymbol{k}_{\text{in}}^j)$
3. **for** $m \leftarrow 1\ to\ N_{\text{step}}$ **do**
4. $O \leftarrow \exp\left[\mathrm{i}k_0\delta n(\boldsymbol{\rho}, z_{\text{ini}}+m\Delta z)\alpha(\boldsymbol{k}_{\text{in}}^j)\Delta z\right]$
5. $u_{\text{prop}}^j(\boldsymbol{\rho}) \leftarrow O\,\mathcal{F}^{-1}\left\{\mathcal{F}\{u_{\text{prop}}^j(\boldsymbol{\rho})\}\exp\left(\mathrm{i}\sqrt{k_{\text{b}}^2 - \|\boldsymbol{k}_{\text{T}}\|_2^2}\,\Delta z\right)\right\}$

($\boldsymbol{k}_{\text{T}}$ is the 2D wavevector coordinates associated with $\boldsymbol{\rho}$, and $\mathcal{F}$ is 2D Fourier transform operator)

<u>Return</u>

$\{u_{\text{prop}}^j(\boldsymbol{\rho})\}_{j=1}^{N_{\text{in}}}$

Pseudo code S2. in-silico clearing RI tomography

<u>Input</u>

$\{u^j(\boldsymbol{\rho})\}_{j=1}^{N_{\text{in}}}$: Complex fields at position z=0, where $\boldsymbol{\rho}=(x,y)$ and N_{in} is the total number of incident angles

$\{\boldsymbol{k}_{\text{in}}^j\}_{j=1}^{N_{\text{in}}}$: 3D incident wavevectors of $\{u^j(\boldsymbol{\rho})\}_{j=1}^{N_{\text{in}}}$

$\{w(j\Delta z)\}_{j=1}^{N_{\text{step}}+N_{pad}}$: The windows function (Tukey windows was used in this study)

Δz: step size in the z-direction

k_{b}: wavenumber in the medium

k_0: wavenumber in the vacuum

N_{block} : number of blocks

N_{step} : steps of each block

N_{pad} : steps for padding

<u>Initialization</u>

$\{u_{\text{prop}}^j(\boldsymbol{\rho})\}_{j=1}^{N_{\text{in}}} = \{u^j(\boldsymbol{\rho})\}_{j=1}^{N_{\text{in}}}$

$\{\delta n_{\text{acc}}(\boldsymbol{\rho}, j\Delta z)\}_{j=1}^{N_{\text{block}}N_{\text{step}}} = 0$

$\{w_{\text{acc}}(j\Delta z)\}_{j=1}^{N_{\text{block}}N_{\text{step}}} = 0$

for $n \leftarrow 0\ to\ N_{\text{block}} - 1$ **do**

$\{\Psi_x(\boldsymbol{\rho}, (nN_{\text{step}} + j)\Delta z)\}_{j=1}^{N_{\text{step}}+N_{\text{pad}}}, \{\Psi_y(\boldsymbol{\rho}, (nN_{\text{step}} + j)\Delta z)\}_{j=1}^{N_{\text{step}}+N_{\text{pad}}}$

$\leftarrow$ run the pseudocode for $\angle \bar{W}^{\text{iMSS}}(\boldsymbol{r})$ calculation in our previous study[2]

with $\{u_{\text{prop}}^{j}(\boldsymbol{\rho})\}_{j=1}^{N_{\text{in}}}$, $\{\boldsymbol{k}_{\text{in}}^{j}\}_{j=1}^{N_{\text{in}}}$, $\delta\boldsymbol{\rho}$, Δz, N_{layer}, k_{b} for $\delta\boldsymbol{\rho}$ of x, y shear direction

$\{\delta n(\boldsymbol{\rho}, (nN_{\text{step}} + j)\Delta z)\}_{j=1}^{N_{\text{step}}+N_{\text{pad}}} \leftarrow$ RI reconstruction by ADMM (Eqs. (S4), (S5))

with $\{\Psi_x(\boldsymbol{\rho}, (nN_{\text{step}} + j)\Delta z)\}_{j=1}^{N_{\text{step}}+N_{\text{pad}}}$, $\{\Psi_y(\boldsymbol{\rho}, (nN_{\text{step}} + j)\Delta z)\}_{j=1}^{N_{\text{step}}+N_{\text{pad}}}$

for $j \leftarrow 1\ to\ N_{\text{step}} + N_{\text{pad}}$ **do**

$\delta n_{\text{acc}}(\boldsymbol{\rho}, (nN_{\text{step}} + j)\Delta z) \leftarrow \delta n_{\text{acc}}(\boldsymbol{\rho}, (nN_{\text{step}} + j)\Delta z) + w(j\Delta z)\delta n(\boldsymbol{\rho}, (nN_{\text{step}} + j)\Delta z)$

$w_{\text{acc}}\left((nN_{\text{step}} + j)\Delta z\right) \leftarrow w_{\text{acc}}\left((nN_{\text{step}} + j)\Delta z\right) + w(j\Delta z)$

if $l < N_{\text{block}}$ do

$\{u_{\text{prop}}^{j}(\boldsymbol{\rho})\}_{j=1}^{N_{\text{in}}} \leftarrow$ run the Pseudocode S1 for BPM

with $\{u_{\text{prop}}^{j}(\boldsymbol{\rho})\}_{j=1}^{N_{\text{in}}}$, $\{\boldsymbol{k}_{\text{in}}^{j}\}_{j=1}^{N_{\text{in}}}$, $\{\delta n(\boldsymbol{\rho}, (nN_{\text{step}} + j)\Delta z)\}_{m=1}^{N_{\text{step}}}$, Δz, k_{b}, k_0

for $j \leftarrow 1\ to\ N_{\text{block}}N_{\text{step}}$ **do**

$\delta n_{\text{acc}}(\boldsymbol{\rho}, j\Delta z) \leftarrow \delta n_{\text{acc}}(\boldsymbol{\rho}, j\Delta z)/w_{\text{acc}}(j\Delta z)$

Return

$\{\delta n_{\text{acc}}(\boldsymbol{\rho}, j\Delta z)\}_{j=1}^{N_{\text{block}}N_{\text{step}}}$

4. Accuracy of BPM

I. Effect of z step size and obliquity factor of beam propagation method on forward model accuracy

We quantified the forward model accuracy of the BPM by comparing it to when the Lippmann–Schwinger equation was solved iteratively. Although iteratively solving the Lippmann–Schwinger equation provides the most accurate solution, it requires heavy memory consumption and large computation times[3], which hinders the simulated measurements in a large volume, as shown in Fig. 2. Therefore, we used the BPM as the forward model in the main text. To demonstrate the sufficient accuracy of the BPM for the forward model, we used the Lippmann–Schwinger equation for small-volume simulated measurements and compared them to the BPM. The sample was a single cell (Fig. S2a), which was used as the building block of the simulated spheroid, as shown in Fig. 2. We calculated the simulated fields using five methods: iteratively solving the Lippmann–Schwinger equation, first Born, conventional Rytov, BPM without the obliquity factor (OF), and BPM with OF. These fields are shown when the voxel size is $\lambda_0/5 \times \lambda_0/5 \times \lambda_0/5$ with incident angles $(\theta_x, \theta_y) = (0°, 0°), (36.8°, 0°)$ in Figs. S2b and c, respectively. Apparently, the first Born method produces large errors owing to its weak scattering approximation. Furthermore, the BPM without the OF shows large errors when the incident angle is significantly large. Moreover, to evaluate the performance quantitatively, we calculated the error against the Lippmann–Schwinger method and plotted it in Fig. S2d as a function of the incident angle. Here, we define the normalized error as

$$Normalized\ error = \frac{\|u_{\mathrm{exact}}(\boldsymbol{r}) - u_{\mathrm{sim}}(\boldsymbol{r})\|_2^2}{\|u_{\mathrm{exact}}(\boldsymbol{r})\|_2^2}, \quad (S6)$$

where $u_{\mathrm{exact}}(\boldsymbol{r})$ and $u_{\mathrm{sim}}(\boldsymbol{r})$ are the simulated measurements using the Lippmann–Schwinger equation and other methods, respectively. The error of the BPM without OF increases as the incident angle increases. Overall, the BPM with OF exhibited the most accurate performance among the computationally efficient models. To see the dependence of the accuracy on the z-step size of the BPM, we plotted the normalized error as a function of the z-step size in Fig. S2e. The graph shows that the z-step size does not significantly affect the forward model accuracy, which is consistent with an existing study[4].

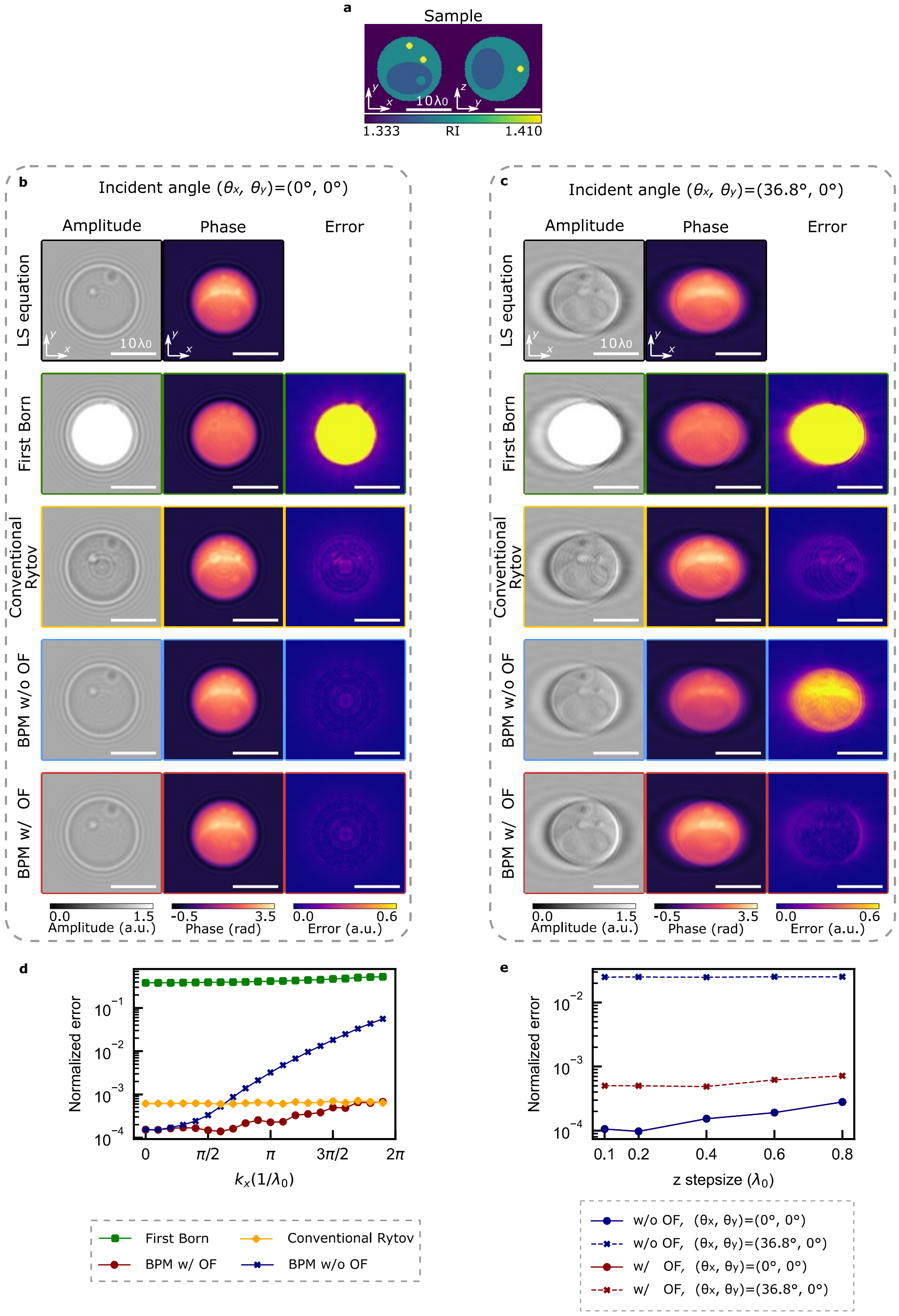

Fig. S2. Accuracy of BPM for simulated measurements of a single cell. a Ground truth of the RI map. **b-c** The simulated measurements by first Born, conventional Rytov, the Lippmann–Schwinger equation, BPM without obliquity factor (OF), and BPM with OF for incident angles of $(\theta_x, \theta_y) = (0°, 0°), (36.8°, 0°)$. The error map is calculated by the absolute value of the difference to fields calculated from the Lippmann–Schwinger equation. **d** The errors of fields by each method (First Born, conventional Rytov, BPM with and without OF) against Lippmann–Schwinger solutions as a function of the incident wavenumber. The step size

of BPM was set as $2\lambda_0/5$. The incident angles are $(\theta_x, \theta_y) = (0°, 0°), (36.8°, 0°)$. **e** The errors of BPM with and without OF against the Lippmann–Schwinger solutions as a function of the z step size.

II. Comparison of 3D RI reconstruction accuracy

To understand the effect of the accuracy of the simulated measurements on the RI reconstruction accuracy, we reconstructed RI maps using MSS-Rytov from different simulated measurements: the Lippmann–Schwinger equation, first Born, conventional Rytov, and BPM without/with OF (Fig. S3). Accuracy was quantified by the NRE defined by Eq. (6) of each reconstruction: Lippmann–Schwinger equation 1.12×10^{-5}, first Born 8.37×10^{-5}, conventional Rytov 1.16×10^{-5}, BPM without OF 1.34×10^{-5}, and BPM with OF 1.12×10^{-5}. The BPM with OF showed performance similar to that of the Lippmann–Schwinger equation, which indicates that the BPM with OF exhibited sufficient accuracy as the forward model.

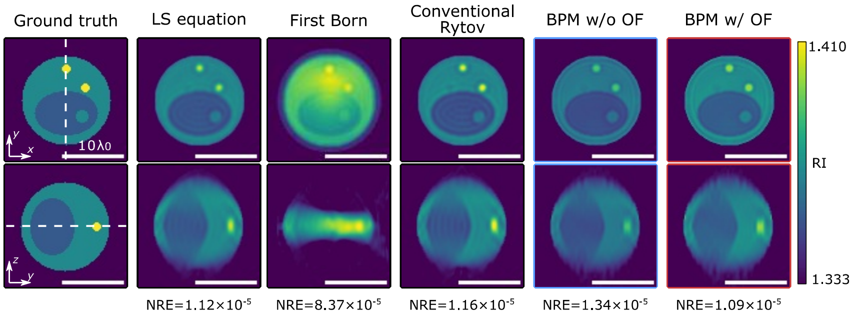

Fig. S3. Simulations comparing the RI maps of a simulated cell reconstructed using different simulated measurements. The x-y and y-z cross-sections of the RI distributions of the ground truth, reconstructed by MSS-Rytov using simulated measurements by the Lippmann–Schwinger (LS) equation, first Born, conventional Rytov, and BPM without/with obliquity factor (OF). The cross-sections are identified using white dashed lines in the ground truth. NREs were 1.12×10^{-5} (Lippmann–Schwinger equation), 8.37×10^{-5} (first Born), 1.16×10^{-5} (conventional Rytov), 1.34×10^{-5} (BPM without OF), and 1.12×10^{-5} (BPM with OF).

III. Wave-backpropagation accuracy of BPM

Next, we quantified the wave-backpropagation accuracy of the BPM to validate its performance when used in in-silico clearing RI tomography. Inaccurate backpropagation leads to errors in wavefront compensation and inadequate suppression of MS and SIA: therefore, we quantified the performance of the BPM when used for wave-backpropagation. We used the simulated measurements from the Lippmann–Schwinger equation and backpropagated them with BPM with/without OF through the ground-truth RI distribution. If the backpropagation is accurate, the backpropagated field becomes a plane wave. We showed the backpropagated fields by BPM without and with OF when the voxel size is $\lambda_0/5 \times \lambda_0/5 \times 2\lambda_0/5$ with incident angles $(\theta_x, \theta_y) = (0°, 0°), (36.8°, 0°)$ in Figs. S4a, b. The backpropagated wave without OF exhibited a relatively large phase error when the incident angle was large. In contrast, The backpropagated wave with the OF showed flat wavefronts, even for a large incident angle. Fig. S4c shows the incident-angle dependence of the residual phase error with and without OF. Here, we defined the residual phase as the mean squared error of the phase. Fig. S4d shows the z-step size dependence of the residual phase error with and without OF. The results indicate that OF is required for the BPM to accurately propagate fields.

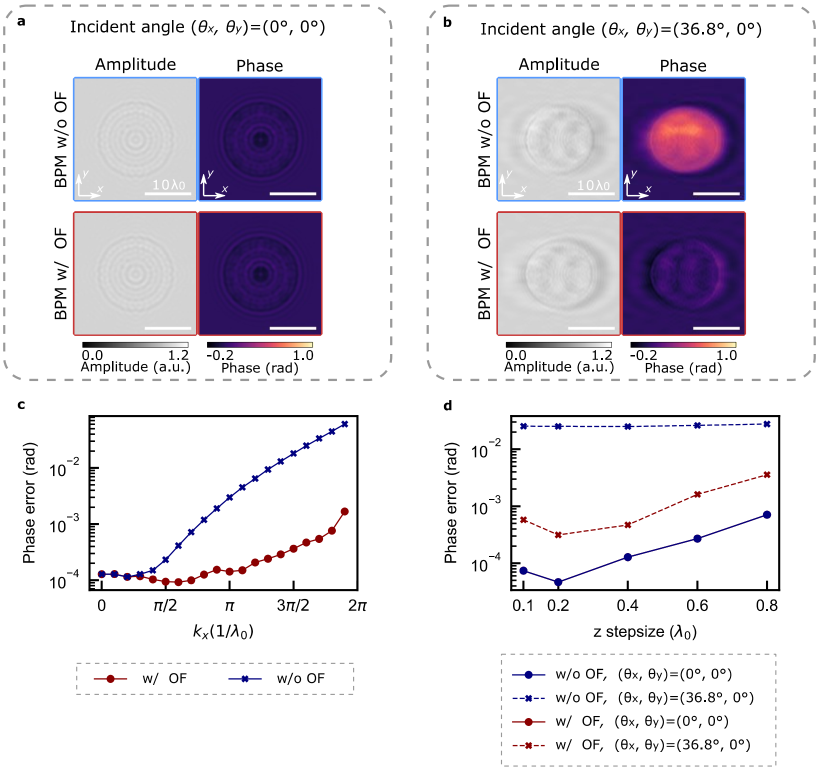

Fig. S4. Wave-backpropagation accuracy of BPM for simulated measurements of a single cell calculated by the Lippmann–Schwinger equation. a, b Backpropagated fields by beam propagation without/with obliquity factor (OF) for incident angles of $(\theta_x, \theta_y) = (0°, 0°), (36.8°, 0°)$, respectively. We backpropagated the simulated measurements calculated by iteratively solving the Lippmann–Schwinger equation. The z step size is $2\lambda_0/5$. **c** Residual phase of fields by BPM with/without OF as a function of the incident wavenumber for the z step size of $2\lambda_0/5$. **d** Residual phase of fields by BPM with/without OF as a function of z step size. The incident angles are $(\theta_x, \theta_y) = (0°, 0°), (36.8°, 0°)$.

5. QPGI images of a simulated multicellular spheroid with and without in-silico clearing

Figure S5 shows the QPGI images of the simulated multicellular spheroid with and without in-silico clearing. The QPGI image without in-silico clearing failed to visualize detailed structures at a depth of $49.2\lambda_0$. Thus, obtaining an image of sufficient resolution beyond this depth was impossible. This leads to the blurred reconstruction of RI distribution without in-silico clearing (middle row in Fig. 2a). In contrast, the QPGI images with in-silico clearing successfully visualized fine structures such as the nuclei and nucleoli. This enabled the unambiguous reconstruction of the RI distribution with in-silico clearing (bottom row in Fig. 2a).

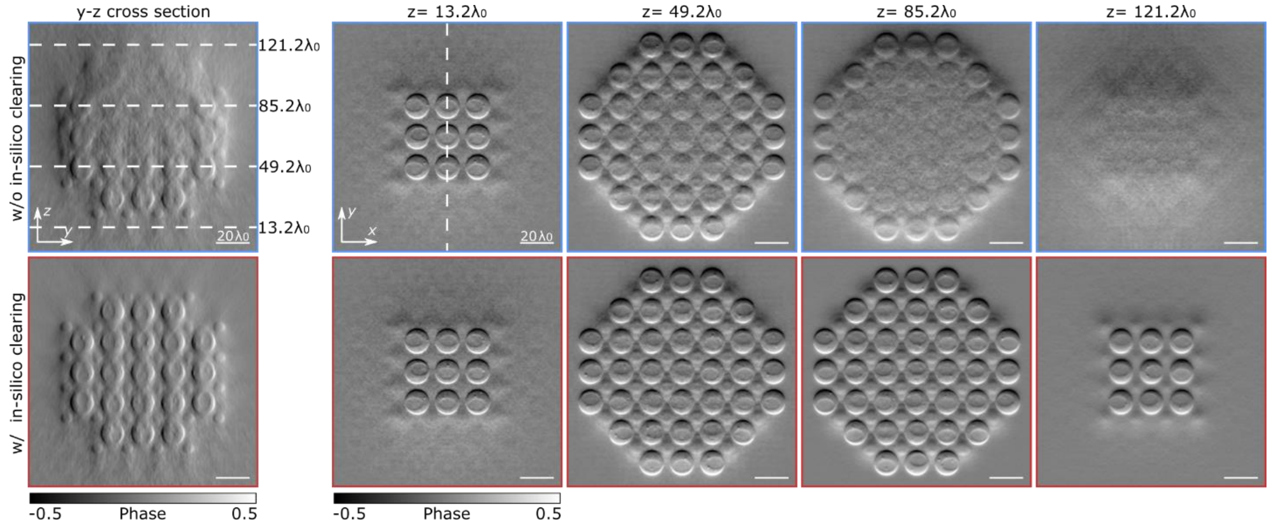

Fig. S5 QPGI images of simulated multicellular spheroid reconstructed with and without in-silico clearing. The cross-sections are identified with white dashed lines each other.

6. Performance of the removal of the wavefront distortion

Herein, we confirm that the wave-backpropagation operation in in-silico clearing RI tomography successfully reduces the wavefront distortion caused by a sample. Fig. S6a shows the fields after propagating each block with incident angles of $(\theta_x, \theta_y) = (0°, 0°), (0°, 49.5°)$. The phase images show that the phase of the fields gradually flattens as they propagate through the blocks. The fields after each block were refocused at the center of the sample after backpropagation for a fair comparison. To quantitatively assess the compensation of the wavefronts, we plotted the single-to-multiple scattering ratio (SMR) and the root mean square error of phase as a function of the block number in Fig. S6b. Here, we defined SMR as

$$\mathrm{SMR} = \frac{\sum_j \| u_\mathrm{S}(\boldsymbol{r}) \|_2^2}{\sum_j \left\| u_\mathrm{M}\left(\boldsymbol{r}; \boldsymbol{k}_\mathrm{in}^j\right) \right\|_2^2}, \tag{S7}$$

where $u_\mathrm{S}(\boldsymbol{r}) \coloneqq (1/N_\mathrm{in}) \sum_j \bar{u}\left(\boldsymbol{r}; \boldsymbol{k}_\mathrm{in}^j\right)$ is a single-scattered wave and $u_\mathrm{M}\left(\boldsymbol{r}; \boldsymbol{k}_\mathrm{in}^j\right) \coloneqq u\left(\boldsymbol{r}; \boldsymbol{k}_\mathrm{in}^j\right) - u_\mathrm{S}(\boldsymbol{r})$ is a multiple-scattered wave[1]. We also defined the root-mean-square error of the phase as $(1/\sqrt{N_\mathrm{in}}) \sum_j \left\| \angle u\left(\boldsymbol{r}; \boldsymbol{k}_\mathrm{in}^j\right) \right\|_2$. The phase error decreases as the block number increases, thereby indicating that the wavefront distortions are successfully compensated. The SMR improved as the block number increased, which indicated that multiple scattering was successfully suppressed owing to the wave-backpropagation operation. It should be noted that the fields after block #7 still show distortion owing to the insufficient accuracy of the RI reconstruction and wave-backpropagation in in-silico clearing. This is a limitation of our implementation, as discussed in the main text.

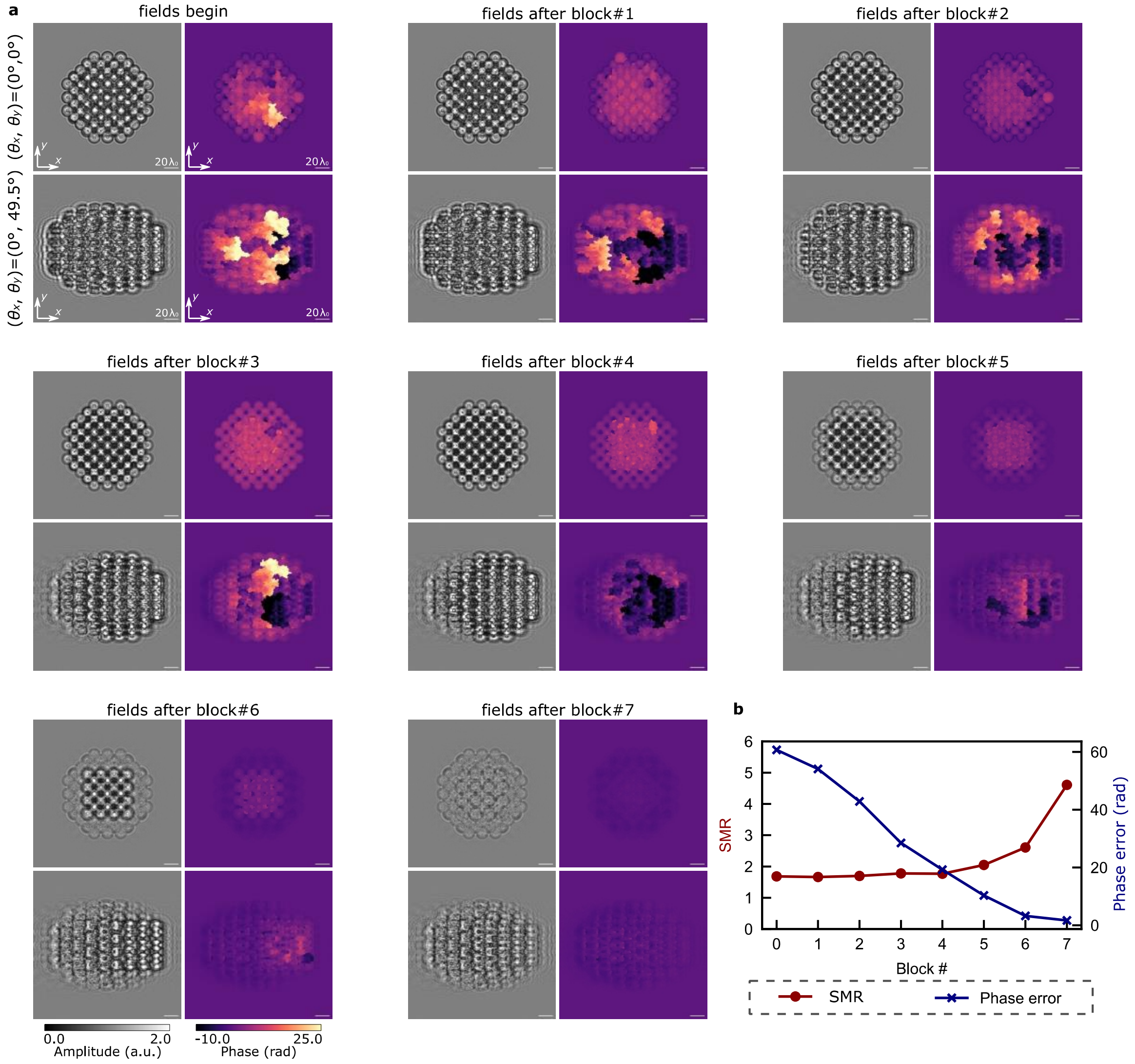

Fig. S6 Numerical evaluation of wave-backpropagation effect on the wavefront of the fields in Fig. 2. a Amplitude and phase of fields after wave-backpropagation after each block for incident angles of $(\theta_x, \theta_y) = (0°, 0°), (0°, 49.5°)$. **b** Single-to-multiple scattering ratio (SMR) and the root mean squared phase error of fields.

7. Conventional Rytov reconstruction of a simulated multicellular spheroid

We reconstructed the RI distribution of the simulated spheroids using the conventional Rytov approximation (Fig. S7). Conventional Rytov failed to completely recover the fine structure of the spheroids as the MS noise caused by the spheroid induces heavy phase distortions, resulting in numerous phase unwrapping failures, as shown in Fig S6 (upper left). The total reconstruction time was 58 s. Therefore, while the conventional Rytov is a computationally efficient method, it does not have the capability of visualizing the simulated spheroid.

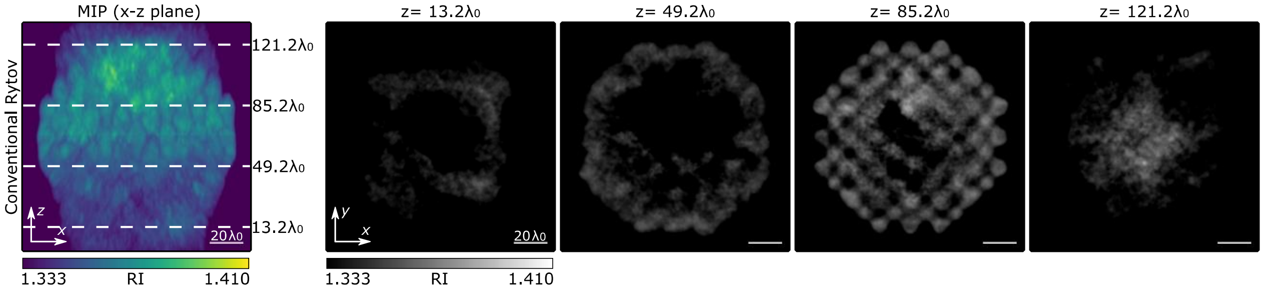

Fig. S7 RI distribution of simulated multicellular spheroid reconstructed by conventional Rytov. The cross-sections are identified with white dashed lines.

8. Low numerical aperture simulation using a simulated spheroid

To assess the influence of numerical aperture on the reconstruction result, we applied in-silico clearing RI tomography to the simulated spheroid in Fig. 2 with different numerical apertures. The numerical aperture determines the performance of optical sectioning. Hence, a low numerical aperture is expected to increase reconstruction errors. We set the numerical aperture as 0.8, 0.6, and 0.4. The illumination and detection used the same values of numerical aperture. The sampling interval for illumination in the k-space was the same as that in Fig. 2, and the number of illumination angles was 221, 121, and 57 for numerical apertures of 0.8, 0.6, and 0.4, respectively. The voxel size, reconstruction volume, and reconstruction parameters were the same as those in Fig.2.

Figures S8a, S9a, and S10a show the QPGI images of the simulated multicellular spheroid with and without in-silico clearing for numerical apertures of 0.8, 0.6, and 0.4, which shows that the z-direction elongation of the image increased as the numerical aperture decreased. Figures S8b, S9b, and S10b shows the reconstructed RI tomograms with and without in-silico clearing for each numerical aperture. In the case without in-silico clearing, cell structures inside the spheroids distorted significantly for depths over $z = 85.2\lambda_0$ for all values of the numerical aperture. On the other hand, in-silico RI tomography caused greater artifacts around the sample (see the cross-section of $z = 13.2\lambda_0$) for any values of numerical aperture. This artifact is caused by the partial reconstruction and insufficient performance of optical sectioning. We calculated the NRE, PSNR, and SSIM to illustrate the performance quantitatively (Figs. S8c, S9c, and S10c). All three metrics showed that RI tomography with in-silico clearing exhibited superior performance than that without in-silico clearing at greater imaging depths for any numerical aperture. Thus, in-silico clearing enhanced the imaging depth for any numerical aperture in this range. However, RI tomography with in-silico clearing showed inferior performance than that without in-silico clearing at a shallow depth owing to the above-mentioned artifacts. These artifacts are a limitation of our approach.

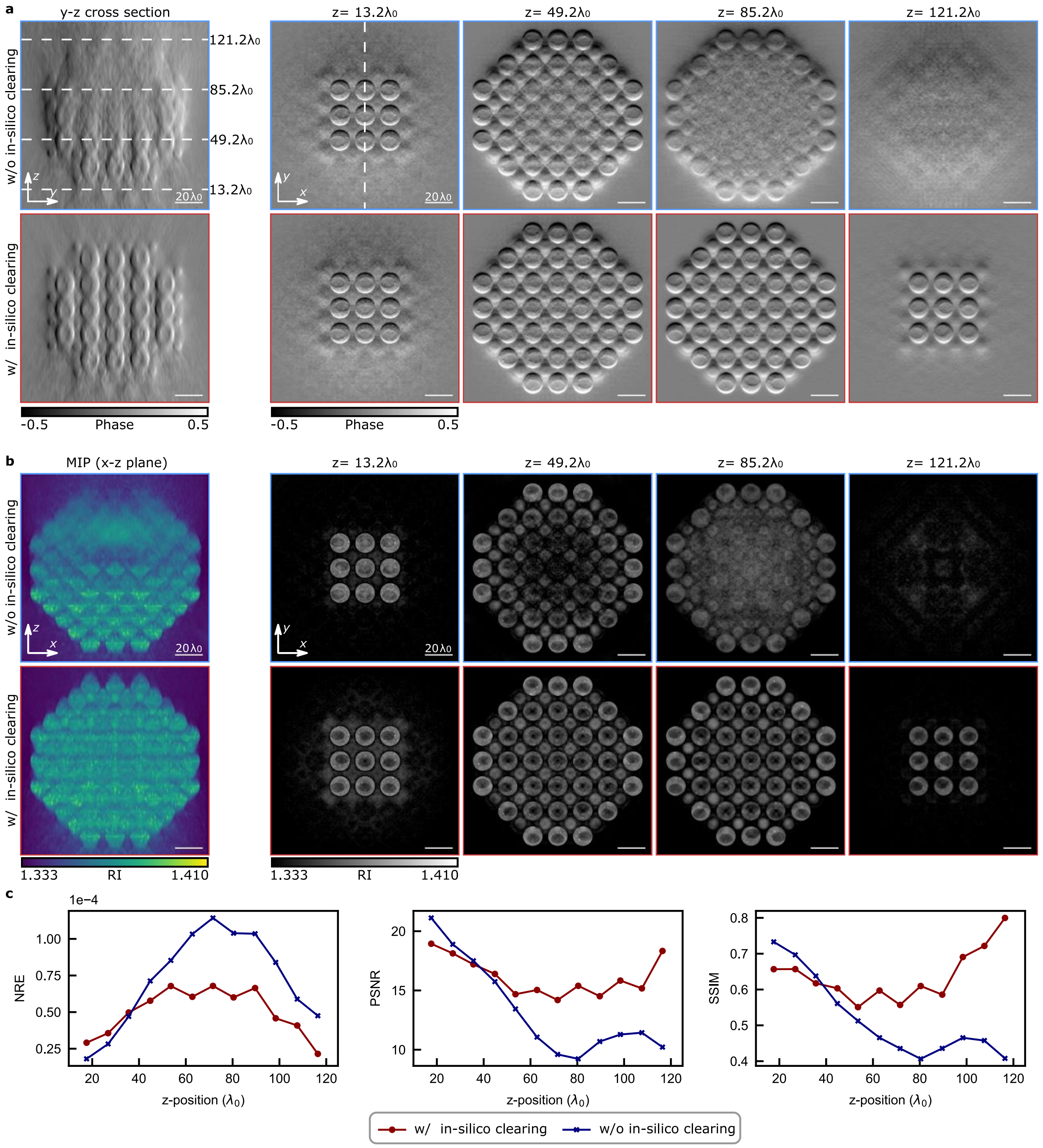

Fig. S8 Numerical evaluation of the in-silico clearing RI tomography performance for a numerical aperture of 0.8. a z-y and x-y cross-sections of QPGI images for simulated multicellular spheroids reconstructed with and without in-silico clearing. The cross-sections are indicated with white dashed lines. **b** Maximum intensity projection (MIP) and cross-sections of RI distributions reconstructed without and with in-silico clearing. The cross-sections are identified with a white dashed line in **a**. **c** NRE, PSNR, and SSIM for cases without and with in-silico clearing as a function of depth.

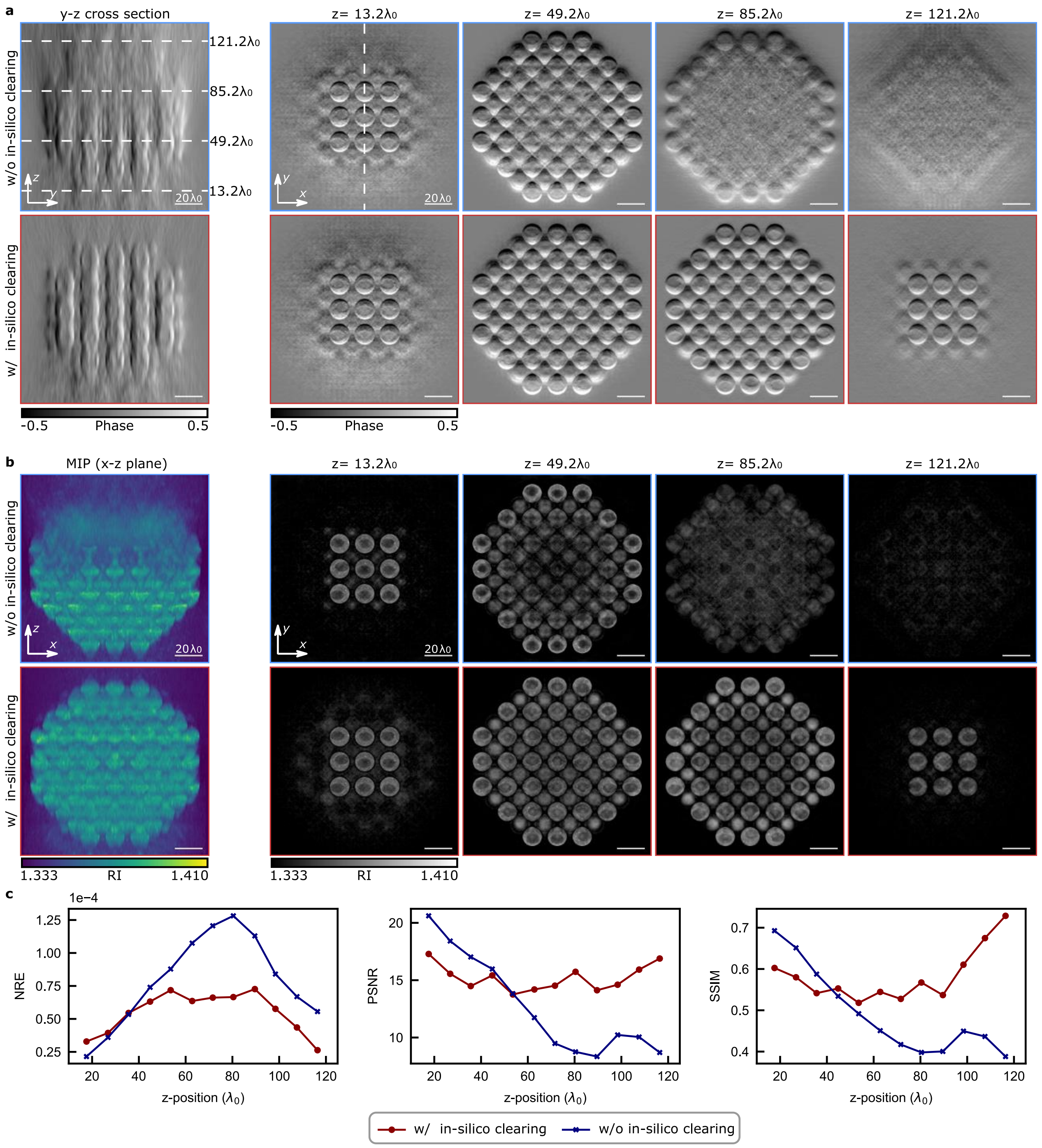

Fig. S9 Numerical evaluation of the in-silico clearing RI tomography performance for a numerical aperture of 0.6. a z-y and x-y cross-sections of the QPGI images for simulated multicellular spheroids reconstructed with and without in-silico clearing. The cross-sections are identified with white dashed lines each other. **b** MIP and cross-sections of the RI distributions reconstructed without and with in-silico clearing. The cross-sections are identified with a white dashed line in **a**. **c** NRE, PSNR, and SSIM for cases without and with in-silico clearing as a function of depth.

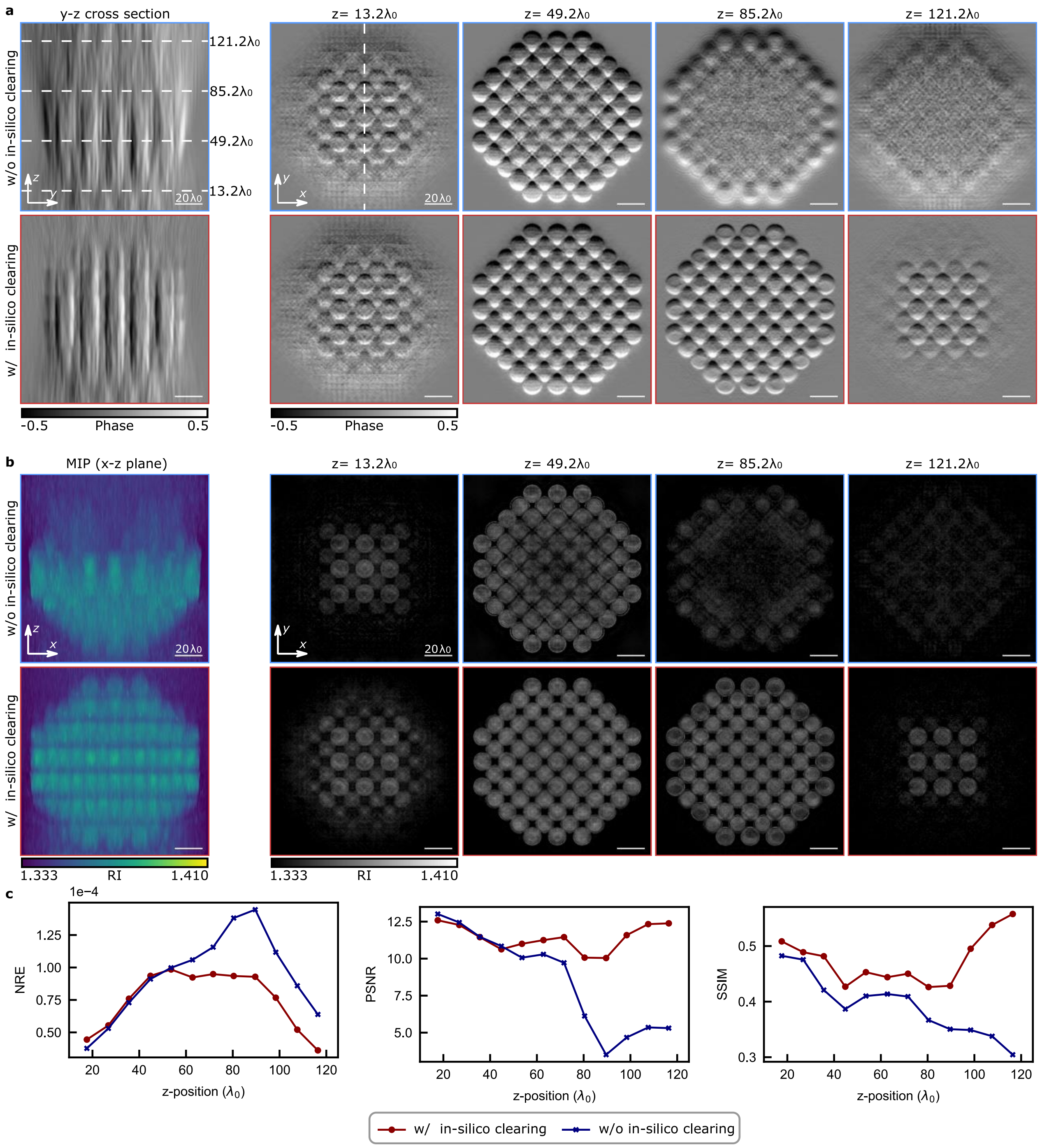

Fig. S10 Numerical evaluation of the in-silico clearing RI tomography performance for a numerical aperture of 0.4. a z-y and x-y cross-sections of the QPGI images for simulated multicellular spheroids reconstructed with and without in-silico clearing. The cross-sections are identified with white dashed lines. **b** MIP and cross-sections of the RI distributions reconstructed without and with in-silico clearing. The cross-sections are identified with a white dashed line in **a**. **c** NRE, PSNR, and SSIM for cases without and with in-silico clearing as a function of depth.

9. RI reconstruction of an absorbing simulated spheroid

To analyze the reconstruction accuracy of the proposed method for an absorptive sample, we applied the proposed approach to a simulated spheroid with a complex-valued RI distribution. In the formulation of MSS-Rytov, the sample is assumed to be transparent, resulting in the reconstruction of only the real part of the RI distribution. Thus, during wave-backpropagation in in-silico clearing RI tomography, we only used the real part of RI distribution. We prepared two absorbing simulated samples: weak absorption and strong absorption (Fig. S11a). These spheroids had the same real-part RI distribution as Fig. 2. To visualize absorption, we calculated the sum of the intensity of all the incident fields using the following equation, $\sum_j \left|u\left(\boldsymbol{r}; \boldsymbol{k}_{\mathrm{in}}^j\right)\right|^2$. Fig 11b shows the intensity for no absorption, weak absorption, and strong absorption, where the intensity for no absorption was calculated from fields used in Fig. 2. For strong absorption, the intensity at the center of the spheroid was almost zero. Figs. S11c and 11d show the reconstructed RI tomograms with and without in-silico clearing for the weak and strong absorption, respectively. Although the contrast degraded at the center of the spheroid for strong absorption, in-silico RI tomography improved imaging depth even with absorption. We plotted the NRE, PSNR, and SSIM for zero, weak, and strong absorption in Fig. S11e. No, and weak absorption showed comparable performance, but strong absorption showed degraded performance. This is because the formulation of iMSS-Rytov assumes that the sample is not absorptive.

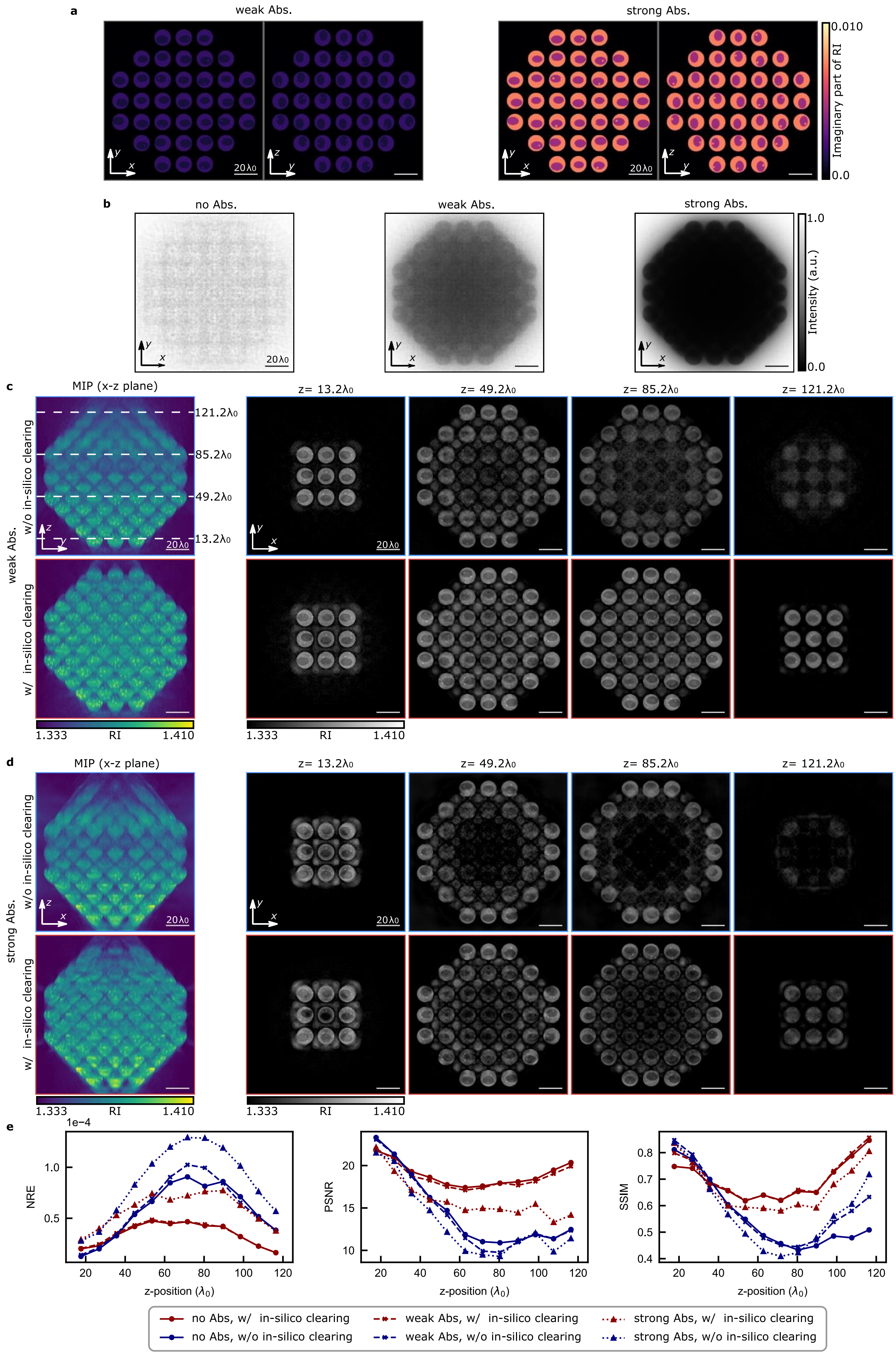

a
weak Abs.
strong Abs.
20λ0
0.010
Imaginary part of RI
0.0
b
no Abs.
weak Abs.
strong Abs.
1.0
Intensity (a.u.)
0.0
c
MIP (x-z plane)
z= 13.2λ0
z= 49.2λ0
z= 85.2λ0
z= 121.2λ0
121.2λ0
85.2λ0
49.2λ0
13.2λ0
weak Abs.
w/o in-silico clearing
w/ in-silico clearing
1.333 RI 1.410
d
strong Abs.
e
NRE
PSNR
SSIM
1e−4
z-position (λ0)
no Abs, w/ in-silico clearing
no Abs, w/o in-silico clearing
weak Abs, w/ in-silico clearing
weak Abs, w/o in-silico clearing
strong Abs, w/ in-silico clearing
strong Abs, w/o in-silico clearing

Fig. S11 Numerical evaluation of the in-silico clearing RI tomography performance for an absorbing sample. a Imaginary part of the RI map for weak and strong absorption. **b** Intensity of fields. The intensity was calculated by the sum of the intensity of all incident fields. **c, d** MIP and cross-sections of the RI distributions reconstructed without and with in-silico clearing for weak and strong absorption. **e** NRE, PSNR, and SSIM for the cases without and with in-silico clearing as a function of depth for no, weak, and strong absorption.

10. Imaging multiple-scattering simulated sample

To demonstrate that our method can suppress MS, we applied our method to an MS-simulated sample consisting of 1241 homogeneous spheres within a volume of $576 \times 576 \times 288$ voxels with $\lambda_0/5 \times \lambda_0/5 \times 2\lambda_0/5$ resolution. The spheres had a diameter of $7\lambda_0$ and were positioned slightly off the body-centered cubic lattice with a lattice constant of $12\lambda_0$. The slight displacement of each sphere was given by a uniform distribution on $[-\lambda_0,\ \lambda_0]$ in each direction to suppress the periodicity of the sample. Figure S12a shows the maximum intensity projection (MIP) and x-y cross-section at $z = 21.6\lambda_0, 45.6\lambda_0, 69.6\lambda_0, 93.6\lambda_0$ of the RI distributions of the ground truth, reconstructed without in-silico clearing, and reconstructed with in-silico clearing, respectively.

To quantitatively assess the performance, we calculated the NRE, PSNR, and SSIM of the RI maps. We split the entire RI distribution into $12\lambda_0$ thick layers, calculated these metrics for each layer, and plotted them as a function of the depth of the center of the layer, as shown in Fig. S12b. The graph shows that the case without in-silico clearing degraded its performance as the depth increased owing to MS noise. Conversely, the case with in-silico clearing significantly increased the accuracy over the depths owing to MS suppression. Therefore, in-silico clearing can successfully suppress MS.

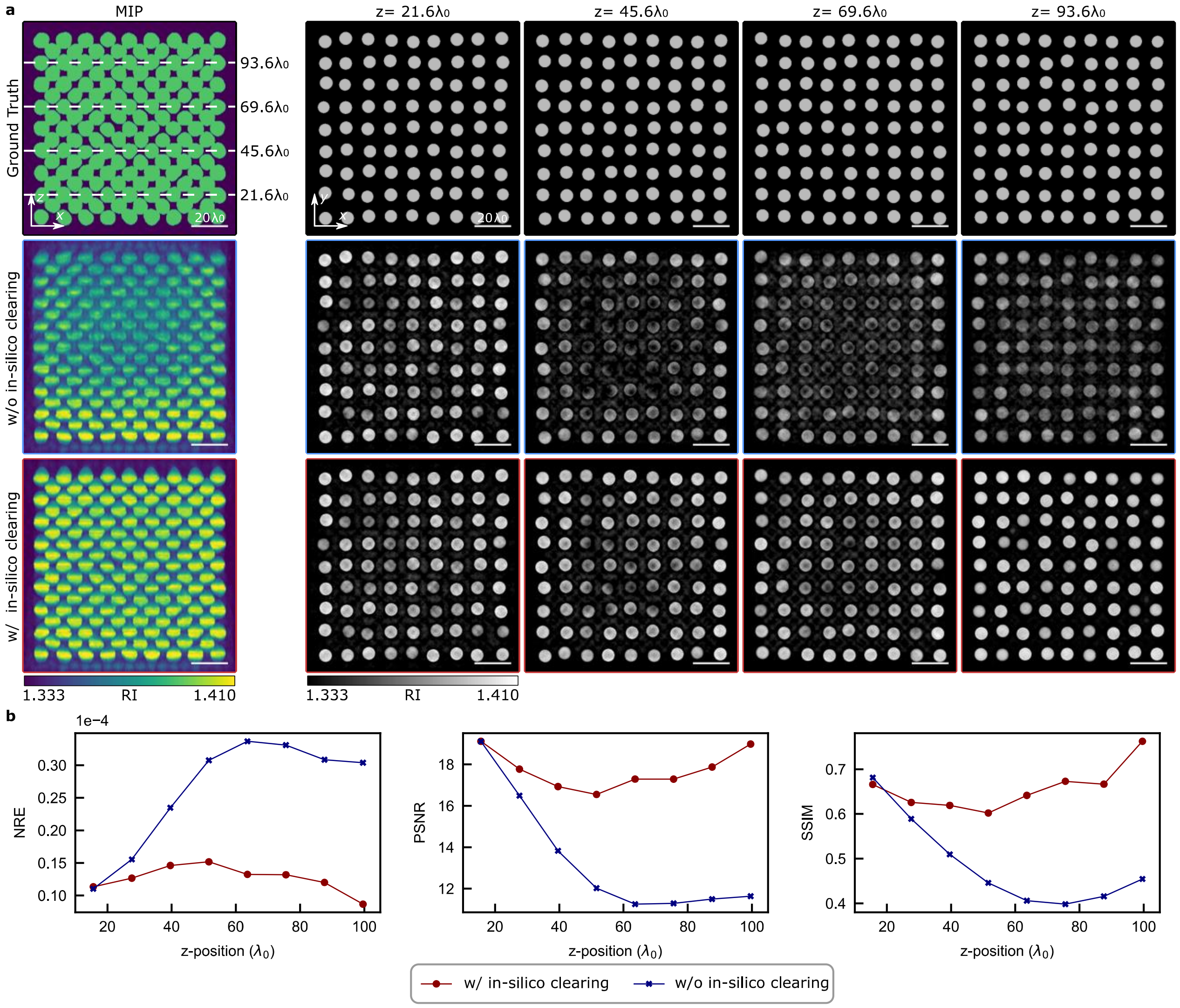

Fig. S12 Numerical evaluation of in-silico clearing performance by simulated multiple-scattering sample. a RI distributions of ground truth, reconstructed without in-silico clearing, and reconstructed with in-silico clearing. **b** NRE, PSNR, and SSIM for the case with and without in-silico clearing as a function of depth. These metrics are computed layer-wise with the thickness of lattice constant $12\lambda_0$.

11. Imaging sample-induced-aberration simulated sample

To demonstrate that the proposed method can suppress SIA, we applied it to an SIA-simulated sample consisting of a sphere with a diameter of $50\,\lambda_0$ within a volume of $288 \times 288 \times 144$ voxels with $\lambda_0/5 \times \lambda_0/5 \times 2\lambda_0/5$ resolution. The sphere has small spheres with a diameter of $2\lambda_0$ at depths of $16.4\lambda_0$, $28.8\lambda_0$, and $41.2\lambda_0$ as indicators of SIA. In this simulation, we used parameters $\tau = 3 \times 10^{-1}$, $\xi = 0.1$, $\mu_1 = 1, \mu_2 = 10, \mu_3 = 5,$ and $\mu_4 = 0.1$. The x-z and x-y cross-sections of the RI maps are shown in Fig. S13a-d. the case without in-silico clearing did not show a small sphere at $z = 41.2\lambda_0$ considering the image of the sphere was spatially shifted due to SIA, whereas the case with in-silico clearing visualized it. Furthermore, we show the cross-section of the small spheres in Fig. S13e-g. The graph shows the better performance of the case with in-silico clearing over the case without in-silico clearing, thereby demonstrating its capability to suppress SIA. Herein, in-silico clearing caused artifacts near the boundaries of the blocks, which were not confirmed in the case without in-silico clearing, owing to the block splitting in in-silico clearing. This is a limitation of our method, as discussed in the main text.

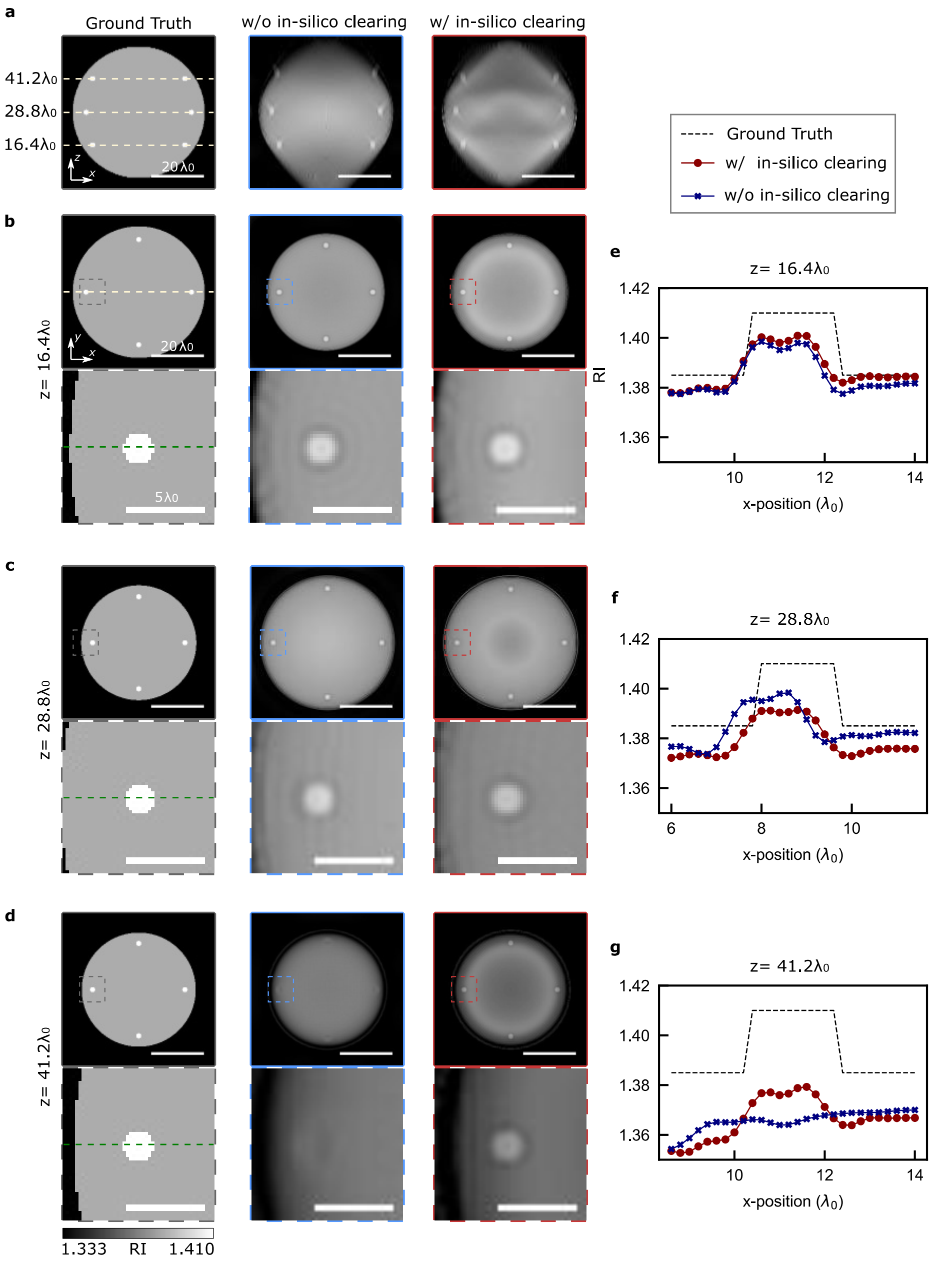

Fig. S13 Numerical evaluation of in-silico clearing performance by simulated sample with sample-induced aberration. a x-z cross-section (identified with white dashed lines in **b**.) of the RI distributions of the ground truth, reconstructed without in-silico clearing and reconstructed with in-silico clearing, **b–d** x-y cross-section (identified with white dashed lines in **a**) of the RI distributions and its close-up (identified with dashed squares) of the RI distributions, and **e–g** x cross-section identified with green dashed lines shown in **b–d**.

12. Experimental setup

Figure S14 shows a schematic of the experimental setup used in this study. A holographic microscope equipped with an off-axis interferometer was constructed. A He-Ne laser (632.8 nm, 20 mW) was used as the illumination source. The laser beam was split into two beams using a 1×2 single-mode fiber coupler (SMFC); one beam served as the reference beam for holography whereas the other beam was delivered to the sample. The sample beam was further illuminated onto the sample at various angles of incidence using a 2D scanning mirror (MR-15-30, Optotune). The scanning pattern was a grid pattern with an illumination numerical aperture of 0.9. The sample was placed between a condenser lens (OB1, Olympus, ×60/1.0 NA, water immersion) and an objective lens (OB2, Olympus, ×60/1.0 NA, water immersion). The beam passed through OB2, and was combined with the reference beam at a polarizing beam splitter (PBS). Then, a polarizer

extracted the specific polarization. Finally, a tube lens (f = 75 mm) formed a magnified image of the sample with an interference fringe on the CMOS camera (JAI, GO-5100M-USB). It took 4.5 s to record a series of images.

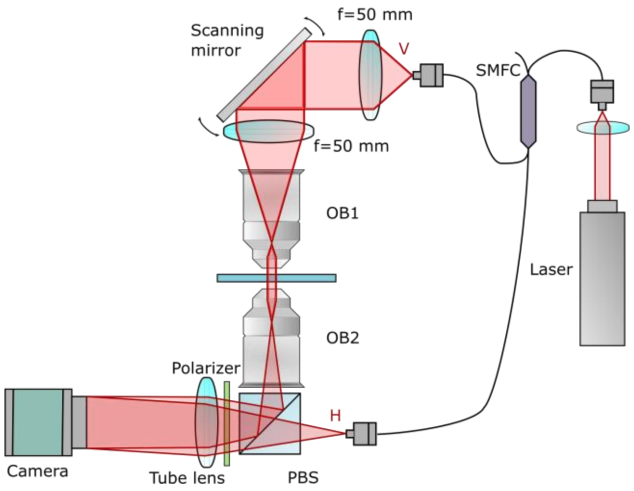

Fig. S14. Schematic of the experiment setup. SMFC: single-mode fiber coupler. PBS: polarizing beam splitter. OB1, OB2: objective lens. H, V: horizontal and vertical polarization. The magnification from the sample plane to CMOS was 25×.

13. Confocal and two-photon imaging of a HepG2 spheroid

In this section, we describe the difficulty of imaging the liver spheroid shown in Fig. 3 by presenting the confocal and two-photon images of a spheroid cultured with the same protocol as Fig. 3 but stained with Hoechst 33342. The images were acquired via Nikon A1 RMP+ multiphoton confocal inverted microscope using a ×40/1.15 NA water dipping objective (CFI Apo LWD Lambda S 40XC WI). The reconstruction volume contained $1024 \times 1024 \times 162$ voxels with a voxel size of $0.155 \times 0.155 \times 0.86$ μm^3. Figure S15 shows the confocal and two-photon images of a HepG2 spheroid. We performed volumetric confocal imaging followed by volumetric two-photon imaging for an identical spheroid. The diameter of the spheroid was approximately 140 μm, which is similar in size to that of the spheroid shown in Fig. 3. Confocal microscopy produced a sharp resolution up to z = 25.0 μm, but it began losing its resolution at z = 58.5 μm and failed to visualize fine structures beyond z = 92.0 μm. Conversely, two-photon microscopy produced an image with sufficient resolution over a wide depth range owing to the long-wavelength excitation and the absence of pin-hole detection. Therefore, visualizing the spheroid observed in Fig. 3, at least by confocal microscopy, was difficult.

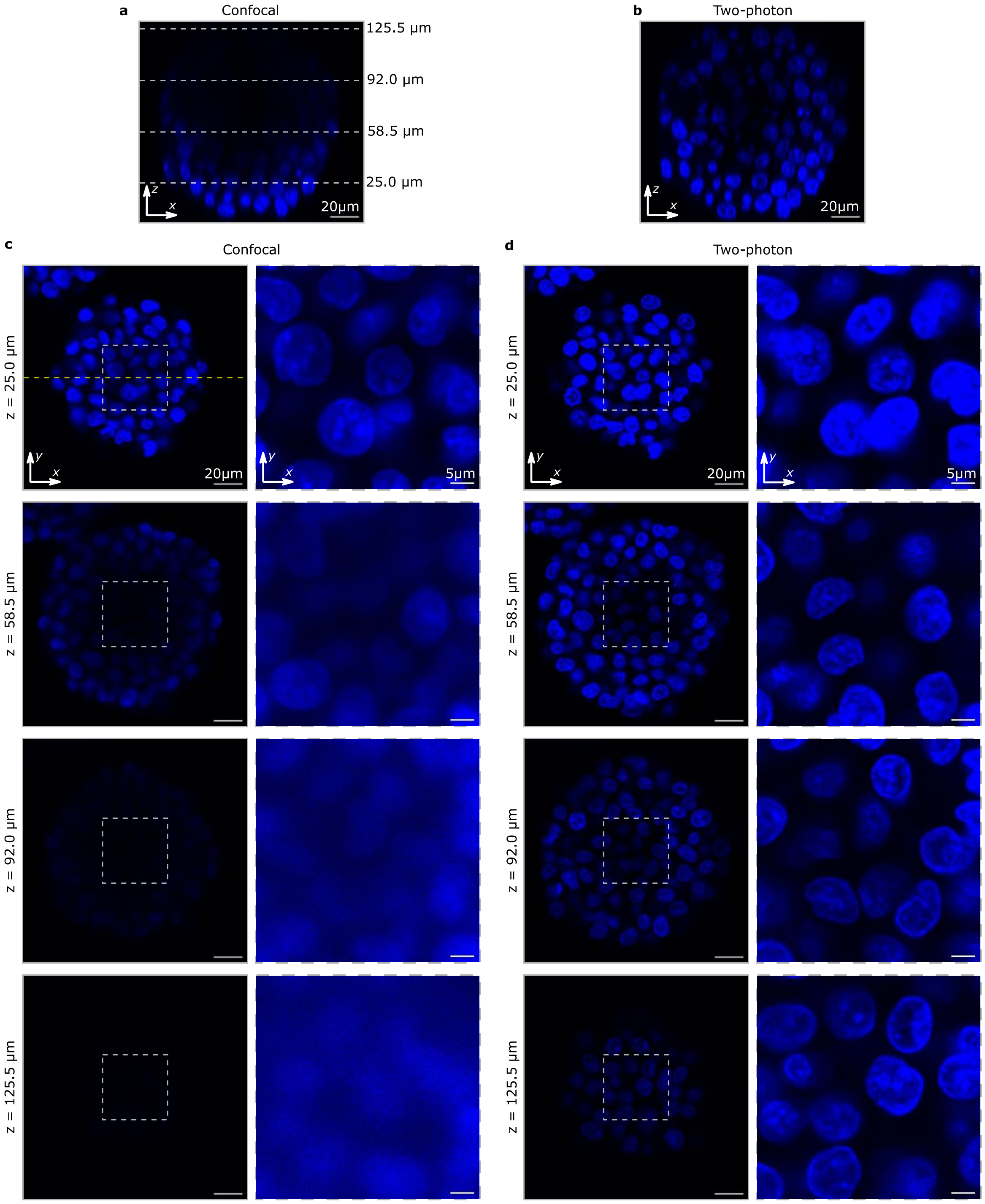

Fig. S15. Confocal and two-photon imaging of the HeqG2 spheroid. a, **b** z-x cross-sections of the Hoechst image via confocal and two-photon microscopy, respectively. The cross-section is identified with a yellow dashed line in **c**. **c**, **d** x-y cross-sections obtained via confocal and two-photon microscopy, respectively. The cross-sections are identified with a white dashed line in **a**. The contrast of the magnified images is adjusted.

14. Conventional Rytov reconstruction of a HepG2 spheroid

We reconstructed the RI distribution of the HepG2 spheroid used in Fig.3 using the conventional Rytov approximation (Fig. S16). Using this approach, it was not possible to determine the individual cell morphology. Therefore, the conventional Rytov was not suitable for the thick samples.

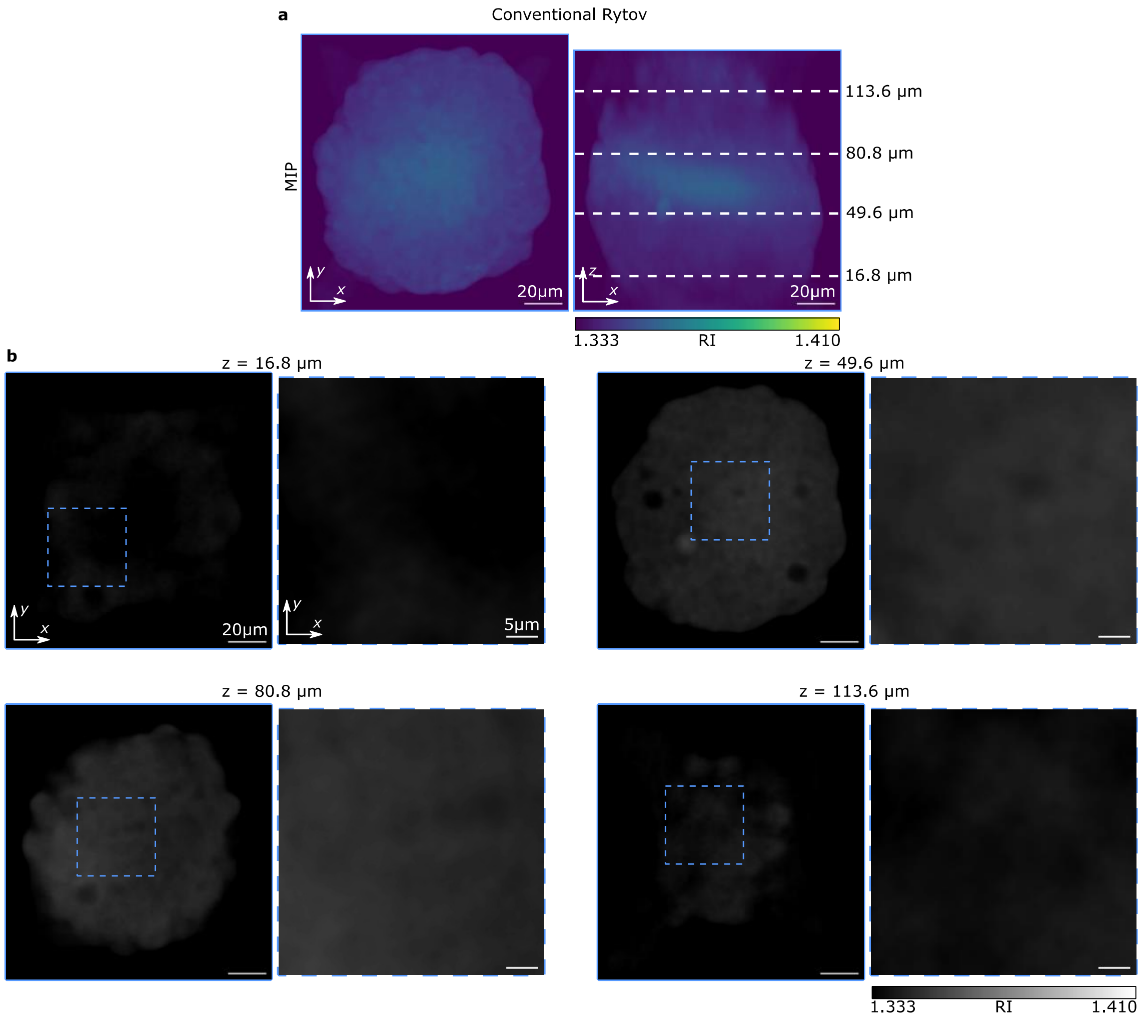

Fig. S16. RI distribution of a HepG2 spheroid reconstructed using conventional Rytov. a Maximum intensity projection of the RI map reconstructed using conventional Rytov. **b** Cross-sections of the RI maps at different z locations (identified by white dashed lines in **a**).

15. RI tomography of different cell-type spheroids without in-silico clearing

Figures S17a–d show the MIPs and lateral cross-sections of the RI distributions of HepG2, A549, A172, and F9 spheroids, respectively, reconstructed without in-silico clearing. In contrast to the case with in-silico clearing (Fig. 4), the case without in-silico clearing failed to visualize fine structures as the depth increased, eventually losing its resolving power at depths of greater than 60 μm.

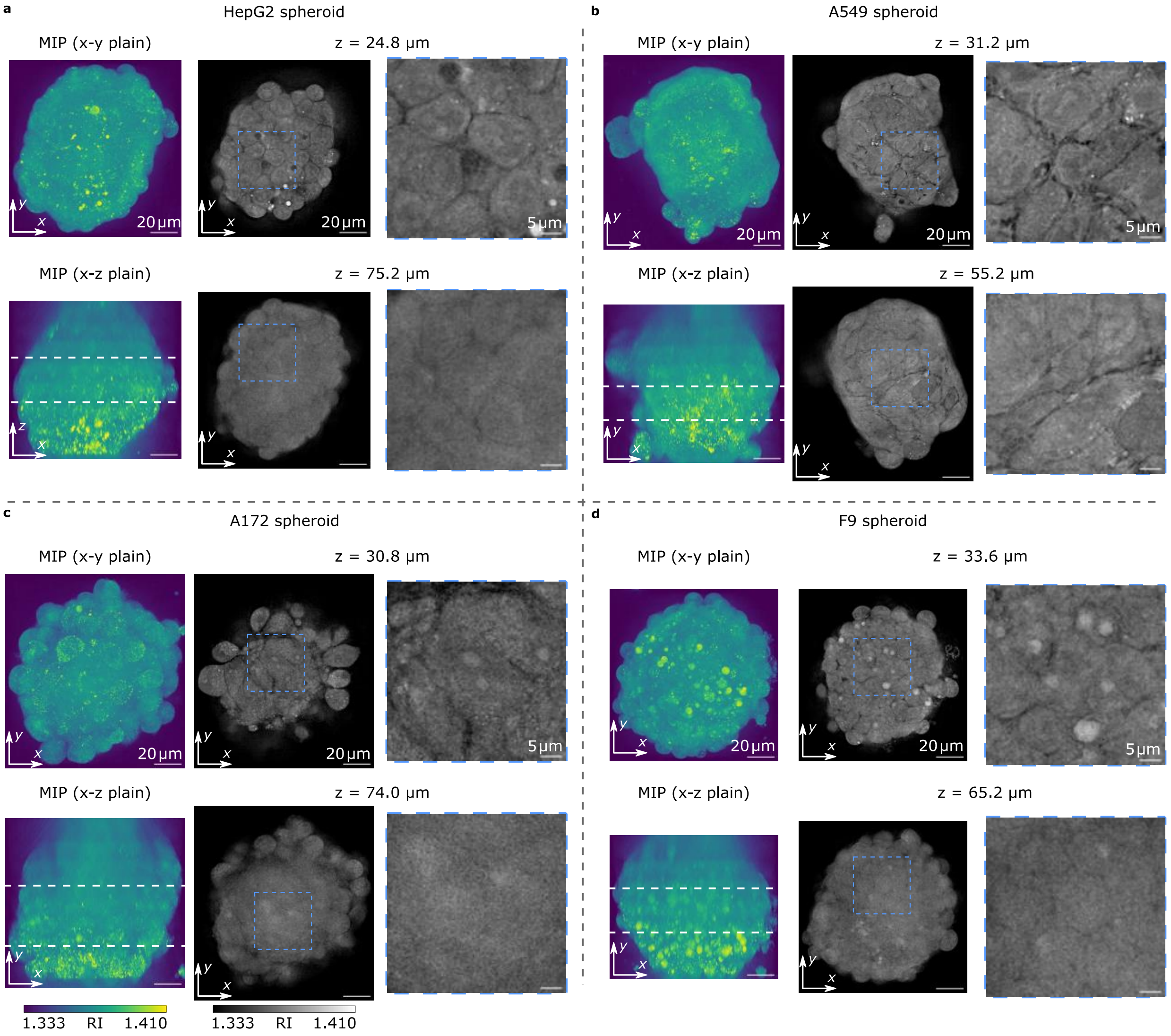

Fig. S17. RI tomography of various cell-type spheroids without in-silico clearing. a–d Maximum intensity projection (MIP) and cross-sections of RI maps for (**a**) HepG2, (**b**) A549, (**c**) A172, and (**d**) F9 spheroids. The depth of the cross-sections in the MIP images is identified using white dashed lines.

16. Confocal imaging of lipid droplet

To confirm that treatment with oleic acid (OA) induces lipid droplet formation, we observed the OA-treated HepG2 spheroids via confocal microscopy. We observed the spheroids stained with Hoechst 33342 (Dojindo Laboratories) and LipiDye II (Funakoshi) treated with 0, 75, 150, and 300 μM OA. We note that the procedures for the spheroid formation and the OA treatment in this observation were not exactly the same as the experiments in Fig. 5. In Fig. 5, the spheroids were formed for 2 days and then treated with OA for 2 days, but in this observation, the spheroids were formed for 3 days and then treated OA for 1 day. The images were acquired by using the Nikon A1 RMP+ using a ×60/1.15 NA water dipping objective (CFI Apo LWD Lambda S 40XC WI). Using the confocal images, we calculated the lipid-droplet volume ratio. As confocal microscopy could not visualize entire spheroids, we specified the analysis area with the same volumes and in the approximately same range of depth for each confocal image (the red dashed-line box in Figs. S18b and c). Next, we defined the lipid-droplet area as where the pixel value of the LipiDye II fluorescent image inside the analysis area was more than 450 (Fig. S18e). Finally, we calculated the lipid-droplet volume ratio as the ratio of the volume of the lipid-droplet area and the volume of the analysis area. We performed this analysis for 2 spheroids at each OA concentration and plotted them in Fig. S18f. We confirmed that OA treatment could induce lipid production in HepG2 spheroids in a dose-dependent manner.

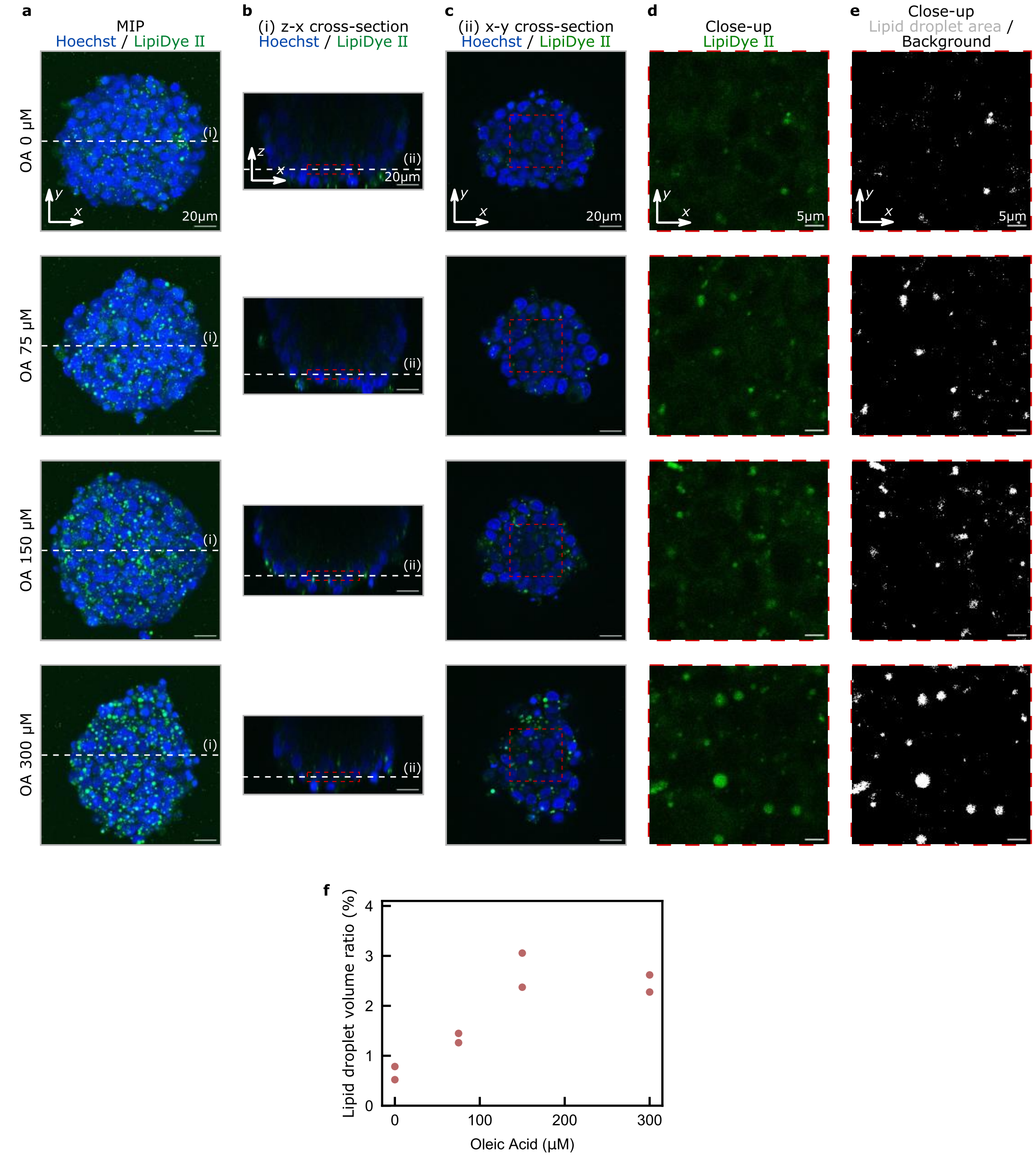

Fig. S18. Confocal images of OA-treated HepG2 spheroids. Confocal images of HepG2 spheroid stained with Hoechst 33342 (blue) and LipiDye II (green) at oleic acid (OA) concentrations of 0, 75, 150, and 300 μM. **a** The maximum intensity projection. **b** The z-x cross-section. The position of cross-sections is identified with white dashed lines in **a**. **c** The x-y cross-section. The depth of cross-sections is identified with white dashed lines in **b**. **d** The magnified images of LipiDye II. The magnified areas are identified with red dashed-line boxes in **c**. **e** The estimated lipid-droplet area. **f** The plot of lipid-droplet volume ratio as a function of OA concentration.

17. Discrimination of non-fragmented/fragmented regions in the spheroid

Herein, we describe a method for discriminating non-fragmented/fragmented regions in the RI distribution of spheroids. First, a thresholded region where $n(\boldsymbol{r}) > 1.34$ is set to the cell foreground (the second column of Fig. S19). Then, we performed a fourth-order Gaussian convolution with standard deviations $\sigma_x, \sigma_y, \sigma_z = 138\ \mathrm{nm}, 138\ \mathrm{nm}, \text{and}\ 400\ \mathrm{nm}$ to the RI distribution to emphasize the area of high spatial variation. We calculated the absolute values of the images (the third column of Fig. S19), and the regions whose values were larger than 2×10^{-4} were extracted and converted into a binary image. Subsequently, a binary closing operation was performed to fill the small holes in the extracted region. We defined the fragmented area as the

intersection of the extracted area and the cell foreground, and defined the non-fragmented area as the cell foreground minus the fragmented area (fourth column of Fig. S19). The pseudocode for discriminating non-fragmented/fragmented regions is provided in Pseudocode S3.

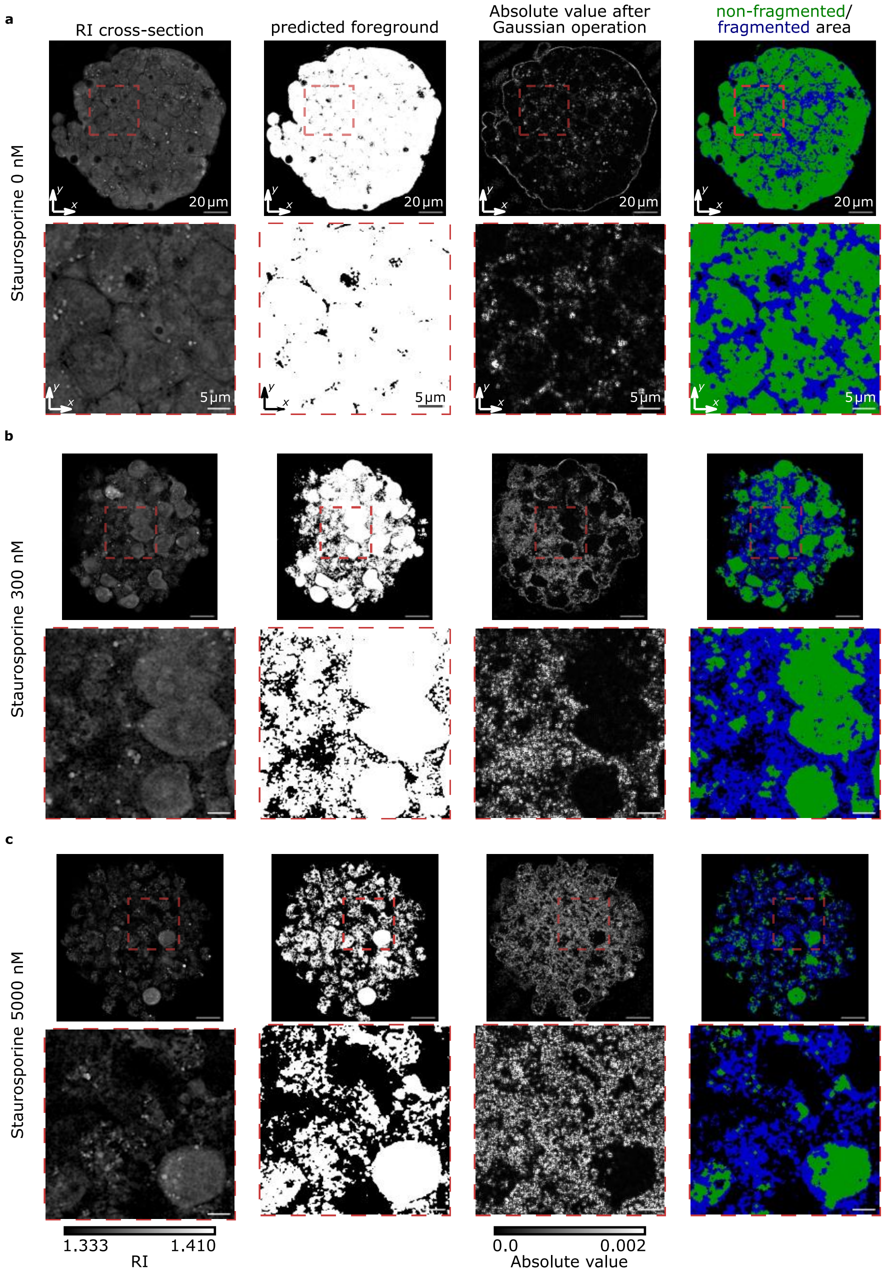

Fig. S19. Discrimination of non-fragmented/fragmented regions from an RI distribution given in Fig. 6. a–c The RI cross-section, predicted foreground (n>1.34, white area), absolute values after the Gaussian operation, and the non-fragmented/fragmented are at staurosporine concentrations of 0, 300, and 5000 nM.

Pseudocode S3. Non-fragmented/fragmented area discrimination

Input

$n(\boldsymbol{r})$: 3D RI distribution at position z=z_{ini}

n_{fg} : threshold value for cell foreground extraction (n_{fg}=1.34 in our case)

Δn_{thre} : threshold value for fragmented cell extraction (Δn_{thre}=2×10^{-4} in our case)

$\boldsymbol{\sigma}$: the standard deviation of a Gaussian kernel ($\boldsymbol{\sigma}$ =(138 nm, 138 nm, 400 nm) in our case)

1. $E_{\mathrm{fg}}(\boldsymbol{r}) \leftarrow n(\boldsymbol{r}) > n_{\mathrm{fg}}$
2. $E_{\mathrm{frag}}(\boldsymbol{r}) \leftarrow$ abs(gaussian_filter($n(\boldsymbol{r})$, $\boldsymbol{\sigma}$, order=4))> Δn_{thre}

   (Here, we used scipy.ndimage.gaussian_filter in python library SciPy and cupy)
3. $E_{\mathrm{frag}}(\boldsymbol{r}) \leftarrow \mathrm{binaly\_closing}(E_{\mathrm{frag}}(\boldsymbol{r}), \mathrm{iteration} = 3)$

   (Here, we used scipy.ndimage.binary_closing in python library SciPy and cupy)
4. $E_{\mathrm{frag}}(\boldsymbol{r}) \leftarrow E_{\mathrm{frag}}(\boldsymbol{r}) \cap E_{\mathrm{fg}}(\boldsymbol{r})$

   ($\cap$ stands for intersection)
5. $E_{\mathrm{nonfrag}}(\boldsymbol{r}) \leftarrow \overline{E_{\mathrm{frag}}(\boldsymbol{r})} \cap E_{\mathrm{fg}}(\boldsymbol{r})$

   ($\bar{A}$ stands for the complement of A)

Return

$E_{\mathrm{nonfrag}}(\boldsymbol{r}), E_{\mathrm{frag}}(\boldsymbol{r})$

18. Observation of staurosporine-induced morphological changes inside A549 spheroids

To demonstrate the generalization performance of in-silico clearing RI tomography for quantification of subcellular morphological change independent of the cell type, we conducted the same analyses as shown in Fig. 6 for A549 spheroids. Figures S20a and b show the RI distributions of A549 spheroids at staurosporine concentrations of 0, 300, and 5000 nM. As the concentration increased, more cells lost their original morphology and showed high spatial variation in the RI map owing to fragmentation due to staurosporine treatment. Figures S20d and e show the spheroid volumes and equivalent diameters, respectively. The graph indicates that the diameters of the spheroids were in the approximate range of 100 μm–150 μm, thereby indicating the size uniformity between the samples. We showed that the cross-sections of the RI maps merged with the non-fragmented/fragmented regions in Fig. S20c, where the image processing was conducted in the same manner as that in Fig. 6. We plotted the non-fragmented ratio in Fig. S20f. The plots were fitted using Hill's model. The estimated relative 50% effective concentration and Hill exponent were $EC_{50} = 158$ nM and $H = 1.36$, respectively.

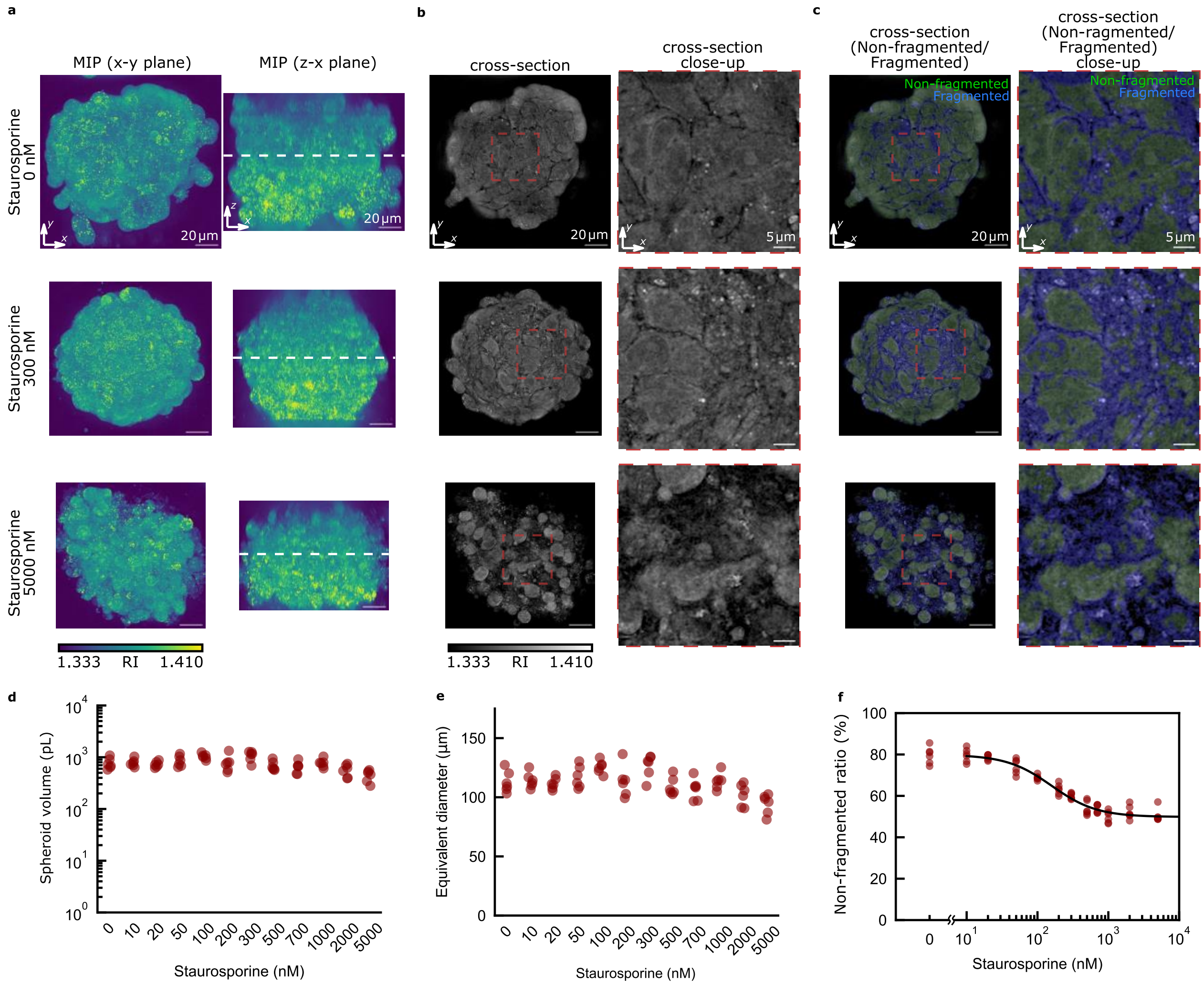

Fig. S20. Semi-quantitative assessment of staurosporine-induced morphological changes in A549 spheroids using in-silico clearing RI tomography. a–c Maximum intensity projection (MIP), cross-sections, and the cross-sections merged with non-fragmented/fragmented areas of RI maps of A549 spheroids at staurosporine concentrations of 0, 300, and 5000 nM. The cross-section is identified with white dashed lines in the MIP image. **d–f** Plots of spheroid volume, equivalent diameter, and non-fragmented ratio of each spheroid at each staurosporine concentration. Six spheroids were measured for each staurosporine

concentration (a total of 72 spheroids). The black solid line in **f** denotes the fitted based on the Hill model. The relative 50% effective concentration $EC_{50} = 158$ nM and the Hill exponent $H = 1.36$.

19. Confocal imaging of staurosporine-treated spheroids

To confirm that treatment with staurosporine causes apoptosis, we observed the staurosporine-treated spheroid via confocal microscopy. We observed HepG2 spheroids stained with Hoechst 33342 (Dojindo Laboratories), Annexin V-FITC and propidium iodide (PI) (Nacalai Tesque) treated with 0 nM and 5000 nM staurosporine (Fig. S21). The procedure to form the spheroids and staurosporine treatment was the same as that used in the experiments in Fig. 6. The images were acquired by using the Nikon A1 RMP+ using a ×60/1.15 NA water dipping objective (CFI Apo LWD Lambda S 40XC WI). In the 5000 nM staurosporine-treated spheroid (Figs. S21b and d), most of the cells in the visualized area were co-stained with Annexin V-FITC and PI, suggesting the presence of late apoptosis. On the other hand, in the control spheroid (0 nM staurosporine, Figs. S21a and c), most of the cells in the visualized region did not show apoptosis. Therefore, apoptotic cell death in HepG2 spheroids induced by staurosporine was confirmed.

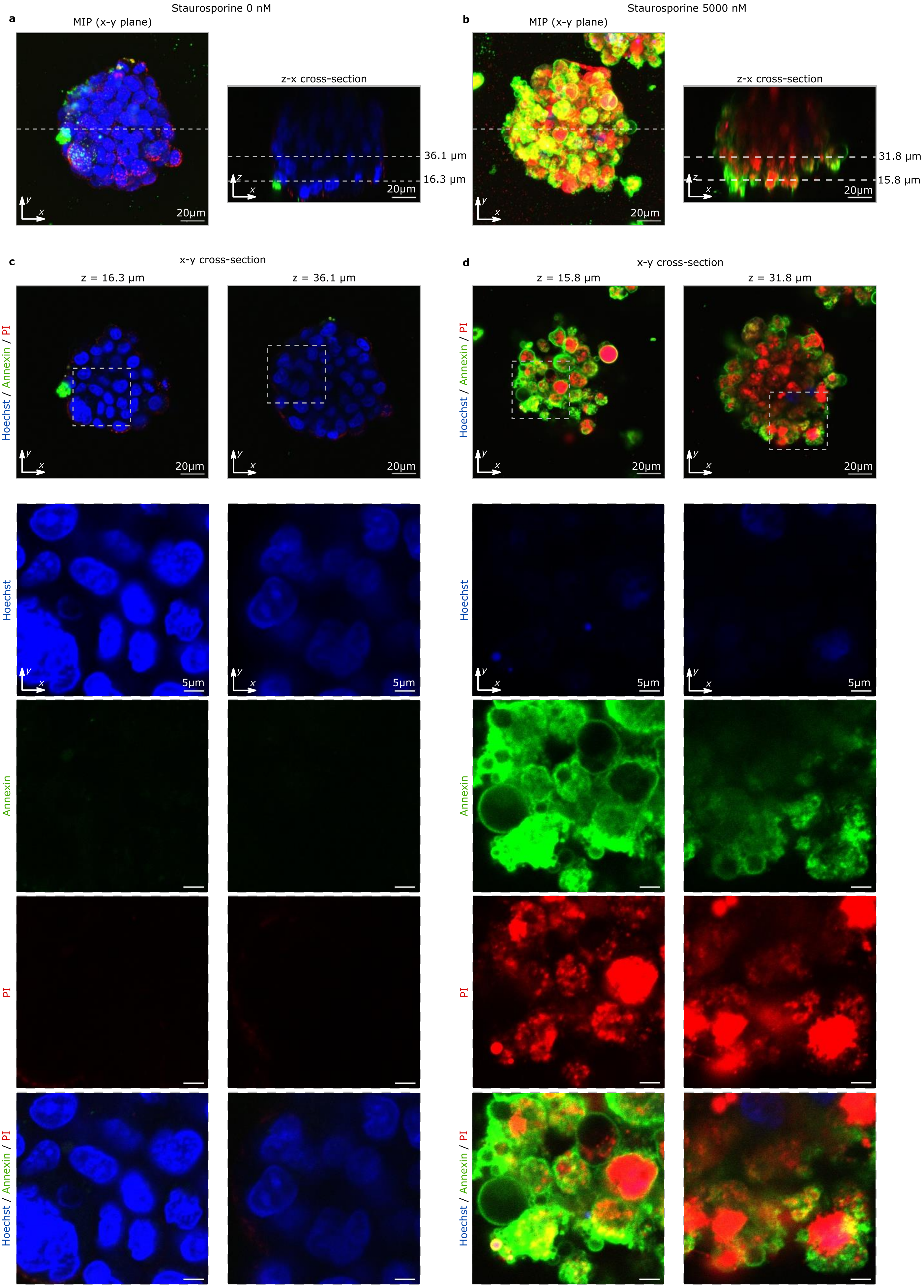
a
Staurosporine 0 nM
MIP (x-y plane)
z-x cross-section
36.1 μm
16.3 μm
20μm
b
Staurosporine 5000 nM
MIP (x-y plane)
z-x cross-section
31.8 μm
15.8 μm
20μm
c
x-y cross-section
z = 16.3 μm
z = 36.1 μm
d
x-y cross-section
z = 15.8 μm
z = 31.8 μm
Hoechst / Annexin / PI
Hoechst
Annexin
PI
5μm

Fig. S21. Confocal images of staurosporine-treated spheroids. a, b MIP and z-x cross-sections of the HepG2 spheroids stained with Hoechst, Annexin V, and propidium iodide (PI) after treatment with 0 nM and 5000 nM staurosporine respectively. **c, d** x-y cross-sections and their close-up.

20. Observation of necrosis-like morphology inside HepG2 and A549 spheroids

Necrosis is predominantly induced by accidental external factors, such as injury and infection, and is morphologically characterized by cytoplasmic swelling, plasma membrane rupture, organelle breakdown, and the absence of chromatin condensation[5]. It is known that high concentrations of ethanol (EtOH) induce necrosis in HepG2 cells[6]. To demonstrate that the proposed method can estimate necrosis inside spheroids, 1.7 M EtOH-treated HepG2 and A549 spheroids were observed. The RI distributions of EtOH-treated HepG2 and A549 spheroids are shown in Fig. S22. Compared to EtOH-untreated spheroids (Figs. 6 and S12, staurosporine 0 nM), there were vacant (low RI) spaces in the cytoplasm in both spheroids owing to the plasma membrane rupture in necrosis. Unlike the staurosporine-treated cells shown in Figs. 6 and S12, the EtOH-treated cells maintained their nuclear structure in both the HepG2 and A549 spheroids. This differs from the staurosporine-treated cells shown in Fig. 6, and S12, where nuclear segmentation occurred and the nuclear structure could not be visualized.

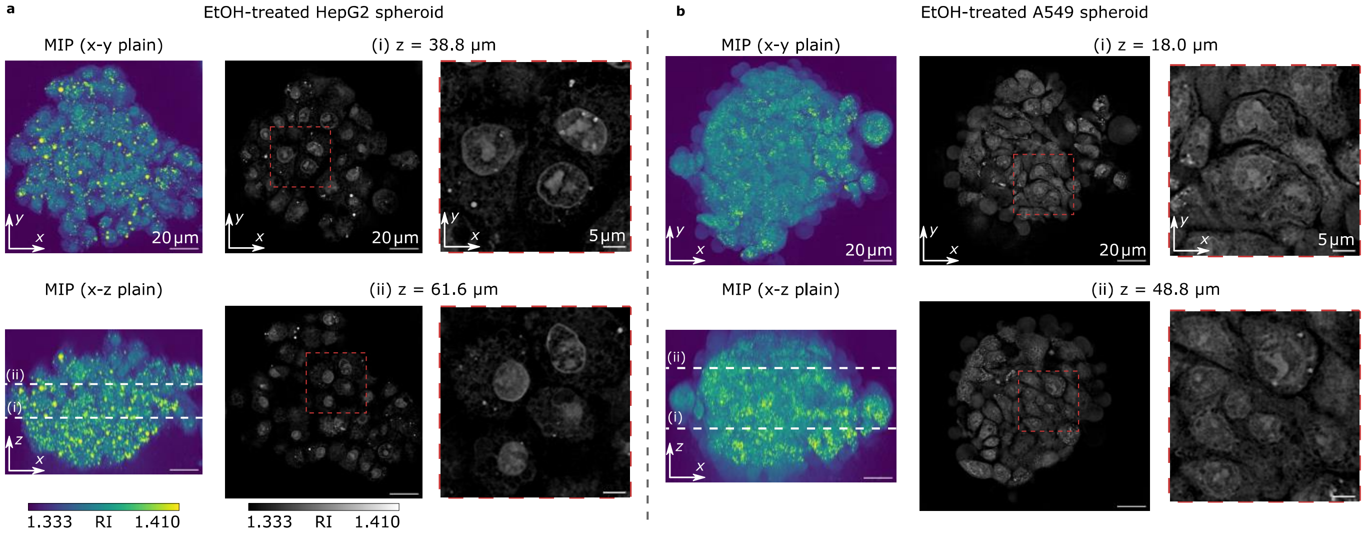

Fig. S22. Observation of necrosis-like morphology inside HepG2 and A549 spheroids. a, b Maximum intensity projection (MIP), and cross-sections of EtOH-treated HepG2 and A549 spheroids, respectively. The cross-section is denoted with white dashed lines in the MIP image.

21. Imaging-depth limit of the proposed method

In this section, we investigate the imaging-depth limit of the proposed method. First, we estimate the scattering mean free path of various samples based on the angle-scanned fields. Next, we observe several spheroids and characterize the imaging-depth limit of the proposed method based on the scattering mean free path.

I. Estimation of scattering mean free path

The scattering mean free path l_{S} is given by the average distance that a photon travels between two consecutive scattering events and is widely used to quantify the maximum imaging depth in the field of deep optical imaging[1,7–9]. When light passes through a turbid media of thickness L and scattering mean free path l_{S}, the intensity of the transmitted ballistic photons I_{B} decays exponentially as follows:

$$I_{\mathrm{B}} = I_0 \exp\left(-\frac{L}{l_{\mathrm{S}}}\right), \tag{S8}$$

where I_0 is the incident intensity. In addition, the scattering mean free path l_{S} is related to the scattering coefficient μ_{S} by the following equation:

$$\mu_{\mathrm{S}} = l_{\mathrm{S}}^{-1}. \tag{S9}$$

Here, we derive the scattering mean free path of the sample by quantifying the transmittance of the ballistic photons obtained by measuring the complex angle-scanned fields. In the experiment setup, complex fields with various illumination angles were measured. We can computationally reproduce focused illumination by superposing the measured field using the following equation: $p(\boldsymbol{r}) \coloneqq \sum_j u\left(\boldsymbol{r}; \boldsymbol{k}_{\mathrm{in}}^{j}\right)$. We calculated $p(\boldsymbol{r})$ for the case without a sample (denoted $p_{\mathrm{B}}(\boldsymbol{r})$) and with a sample (denoted $p_{\mathrm{Sample}}(\boldsymbol{r})$) from the measured complex fields acquired under the same illumination intensity, as shown in in Fig. S22a and b. Here, the interval of the point spread functions is proportional to the inverse of the sampling interval in Fourier space. Compared to the sharp-peak distribution of $p_{\mathrm{B}}(\boldsymbol{r})$, which contains only ballistic photons, $p_{\mathrm{Sample}}(\boldsymbol{r})$ was distorted and lost peaks near the center of the sample due to multiple scattering (Fig. S22a). To derive the transmittance, we need to extract ballistic photons from $p_{\mathrm{Sample}}(\boldsymbol{r})$. Let $p_{\mathrm{MS}}(\boldsymbol{r})$ be the multiple-scattering component in $p_{\mathrm{Sample}}(\boldsymbol{r})$, then $p_{\mathrm{Sample}}(\boldsymbol{r})$ can be written as $p_{\mathrm{Sample}}(\boldsymbol{r}) = t_{\mathrm{B}} p_{\mathrm{B}}(\boldsymbol{r}) + p_{\mathrm{MS}}(\boldsymbol{r})$, where t_{B} is the amplitude transmittance of the ballistic photons, and $p_{\mathrm{B}}(\boldsymbol{r})$ and $p_{\mathrm{MS}}(\boldsymbol{r})$ are orthogonal in the complex Hilbert space equipped with inner product $\langle f, g\rangle = \int f(\boldsymbol{r}) g^{*}(\boldsymbol{r}) \mathrm{d}\boldsymbol{r}$. Thus, we can calculate the power transmittance of ballistic photons T_{B} as follows:

$$T_{\mathrm{B}} = \frac{I_{\mathrm{B}}}{I_0} = |t_{\mathrm{B}}|^2 = \frac{\left|\int_{\boldsymbol{r}\in A} p_{\mathrm{Sample}}(\boldsymbol{r}) p_{\mathrm{B}}^{*}(\boldsymbol{r}) \mathrm{d}\boldsymbol{r}\right|^2}{\left|\int_{\boldsymbol{r}\in A} p_{\mathrm{B}}(\boldsymbol{r})\, p_{\mathrm{B}}^{*}(\boldsymbol{r}) \mathrm{d}\boldsymbol{r}\right|^2}, \tag{S10}$$

where we set the area of analysis A as the small square region shown in Fig. S23b, which has a side of length 7.3 µm at the center of the sample, considering its spherical shape. From Eqs. (S8) and (S10), we can obtain l_{S} by $l_{\mathrm{S}} = -L/\log T_B$.

We estimated the scattering mean free path l_{S} and scattering coefficient μ_{S} for various cell-type spheroids using the aforementioned equations, where thickness L was derived from the reconstructed tomogram. We calculated l_{S} and μ_{S} for three spheroids for each cell type, where spheroids with a diameter of approximately 100 µm were used. The estimated l_{S} and μ_{S} are plotted in Figs. S23c and d. The average values of the scattering mean free paths were 33.2, 21.1, 17.1, 27.9, and 45.0 µm for HepG2, A549, A172, F9, and HCT116, respectively. In addition, the average values of the scattering coefficients were 323.9, 474.4,

590.5, 364.1, and 222.3 cm^{-1} for HepG2, A549, A172, F9, and HCT116, respectively. Based on the results, it was determined that the A172 spheroids exhibited the strongest multiple scattering.

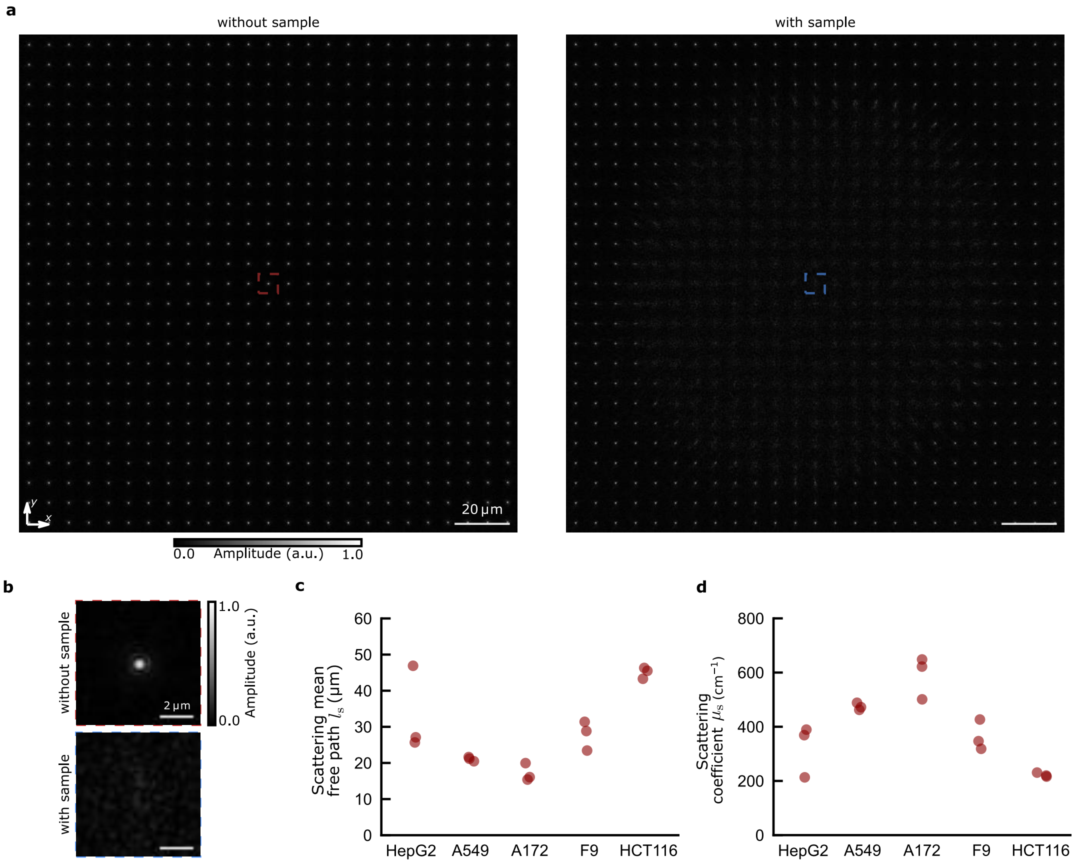

Fig. S23. Estimation of the scattering mean free path. a The diffracted fields under multiple-point illumination with and without a sample. The sample is a HepG2 spheroid used in Fig. 3. The multiple-point illumination was computed from the angle-scanned fields used in Fig. 3. In the case with a sample, the point illumination was heavily distorted. **b** The magnified images at the center of **a**. **c** Estimated scattering mean free paths l_S. The average values of the scattering mean free paths are 33.2, 21.1, 17.1, 27.9, and 45.0 μm for HepG2, A549, A172, F9, and HCT116, respectively. **d** Estimated scattering coefficients μ_S. The average of the scattering coefficients are 323.9, 474.4, 590.5, and 364.1 cm^{-1} for HepG2, A549, A172, and F9, respectively.

II. Characterization of the imaging-depth limit of the proposed method

Next, we investigate the imaging-depth limit of the proposed method. We investigated three types of spheroids (HCT116, HepG2, and A172). In this study, we evaluated the imaging depth based on whether the nucleolus could be imaged since this structure is a common intracellular fine structure in eukaryotic cells. In this experiment, the condenser lens in the experimental setup shown in Fig. S14 was replaced with an achromat aplanatic condenser lens (Nikon, C-AA) to observe a larger sample. Figures. S24, S25, and S26 are the reconstructed RI distributions without and with in-silico clearing of HCT116, HepG2, and A172, respectively. In the case without in silico clearing, entire spheroids could not be imaged for all cell types. Notably, the A172 spheroid, which had the smallest mean scattering free path ($l_S = 17.1$ μm), could be imaged up to 33.2 μm, which corresponded to $1.94 l_S$. In contrast, in-silico clearing RIT successfully imaged the nucleoli inside cells in HCT116 and HepG2 spheroids. However, this technique facilitated the imaging of nucleoli in the A172 spheroid up to 116.0 μm, which corresponded to $6.78 l_S$, although it was unable to image nucleoli beyond this depth. This is the imaging-depth limit of the proposed method.

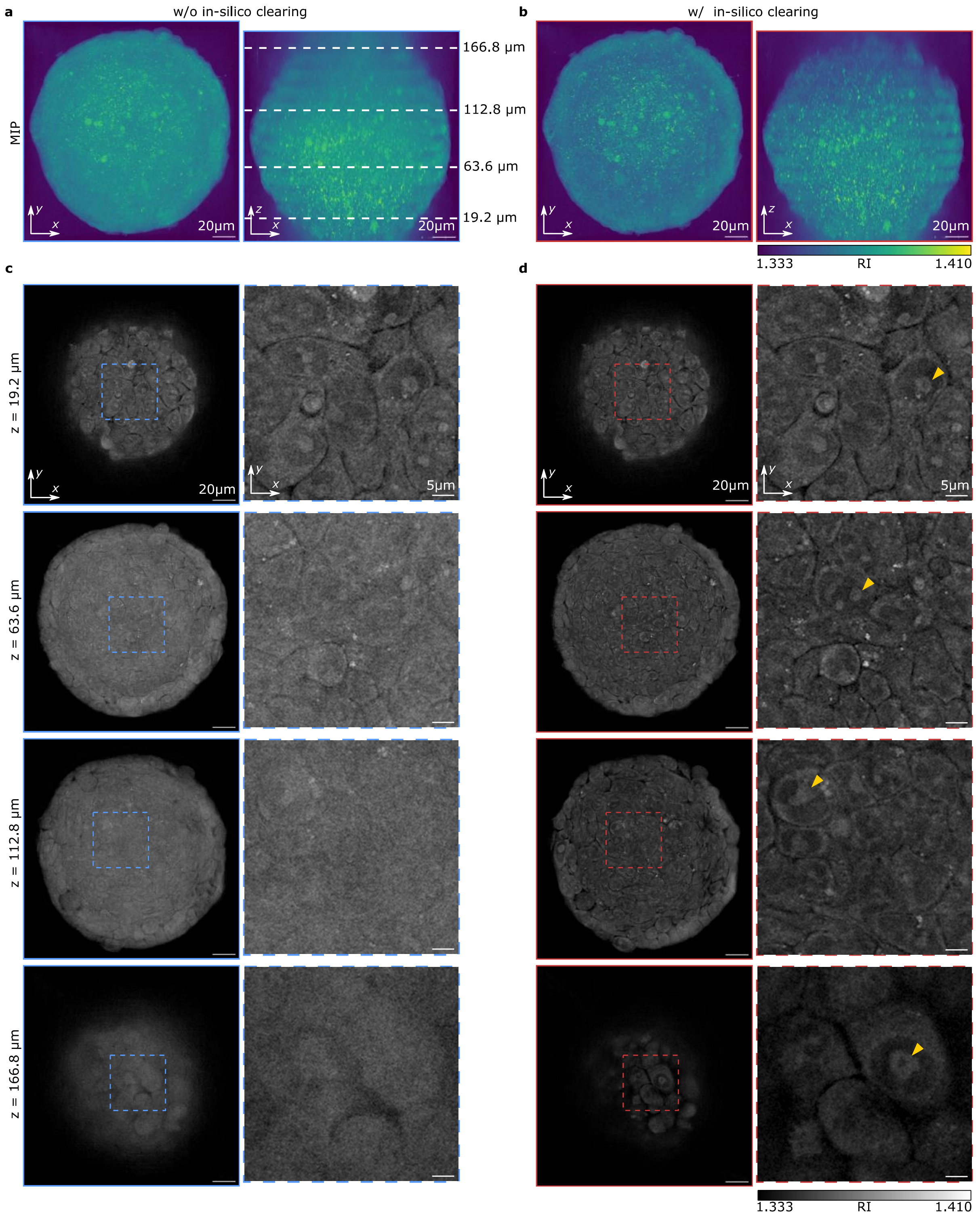

Fig. S24. Imaging an HCT116 spheroid. a, **b** Maximum intensity projection of the RI map reconstructed without in-silico clearing and with in-silico clearing, respectively. **c**, **d** Cross-sections of the RI maps at different z locations (identified by white dashed lines in a), where nucleoli are identified using yellow arrowheads. In the case of in-silico clearing, the structure of the nucleoli can be confirmed over the entire volume.

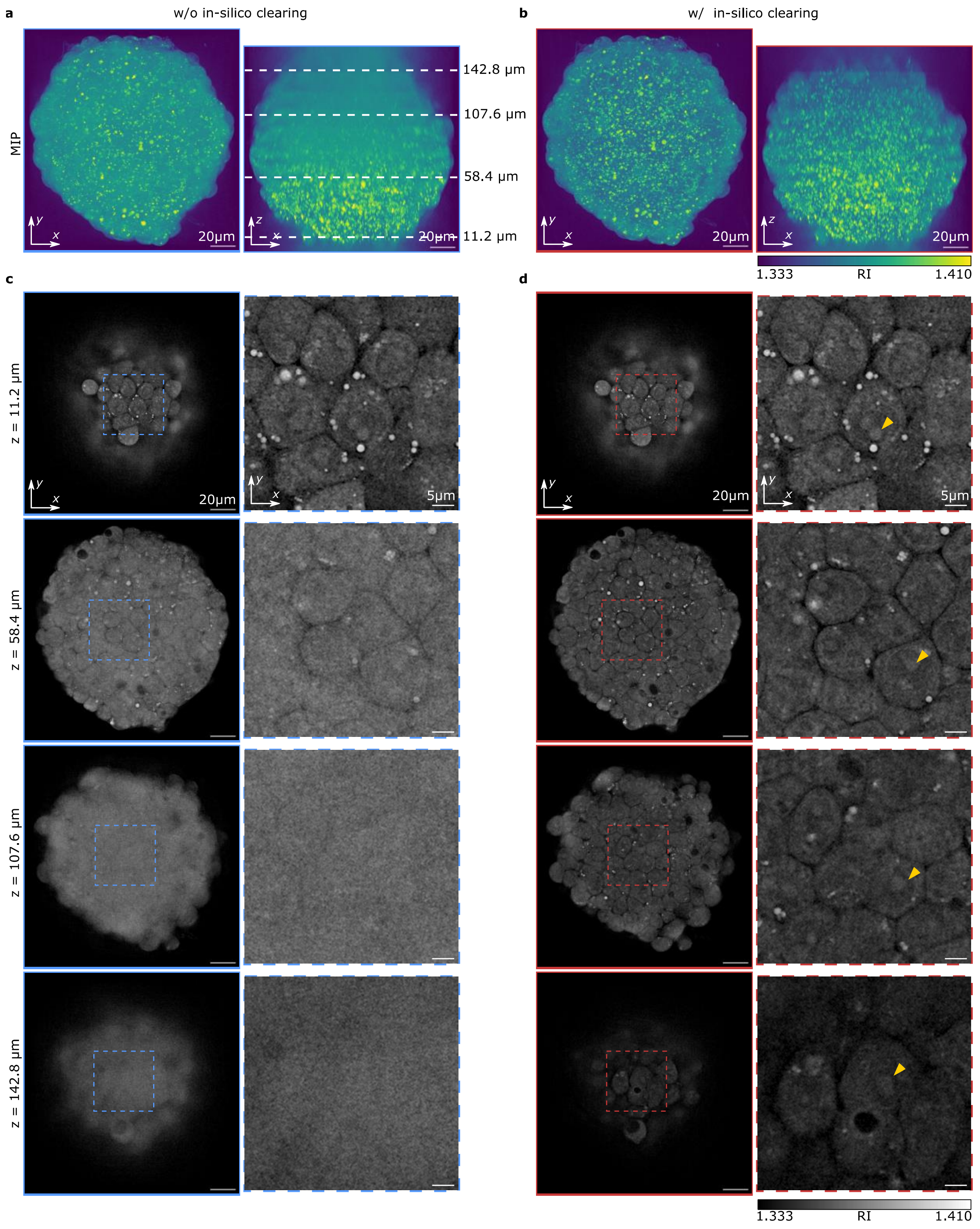

Fig. S25. Imaging a HepG2 spheroid. a, **b** Maximum intensity projection of the RI map reconstructed without and with in-silico clearing, respectively. **c**, **d** Cross-sections of the RI maps at different z locations (identified by white dashed lines in a), where nucleoli are identified by yellow arrowheads. In the case of in-silico clearing, the structure of the nucleoli can be confirmed over the entire volume.

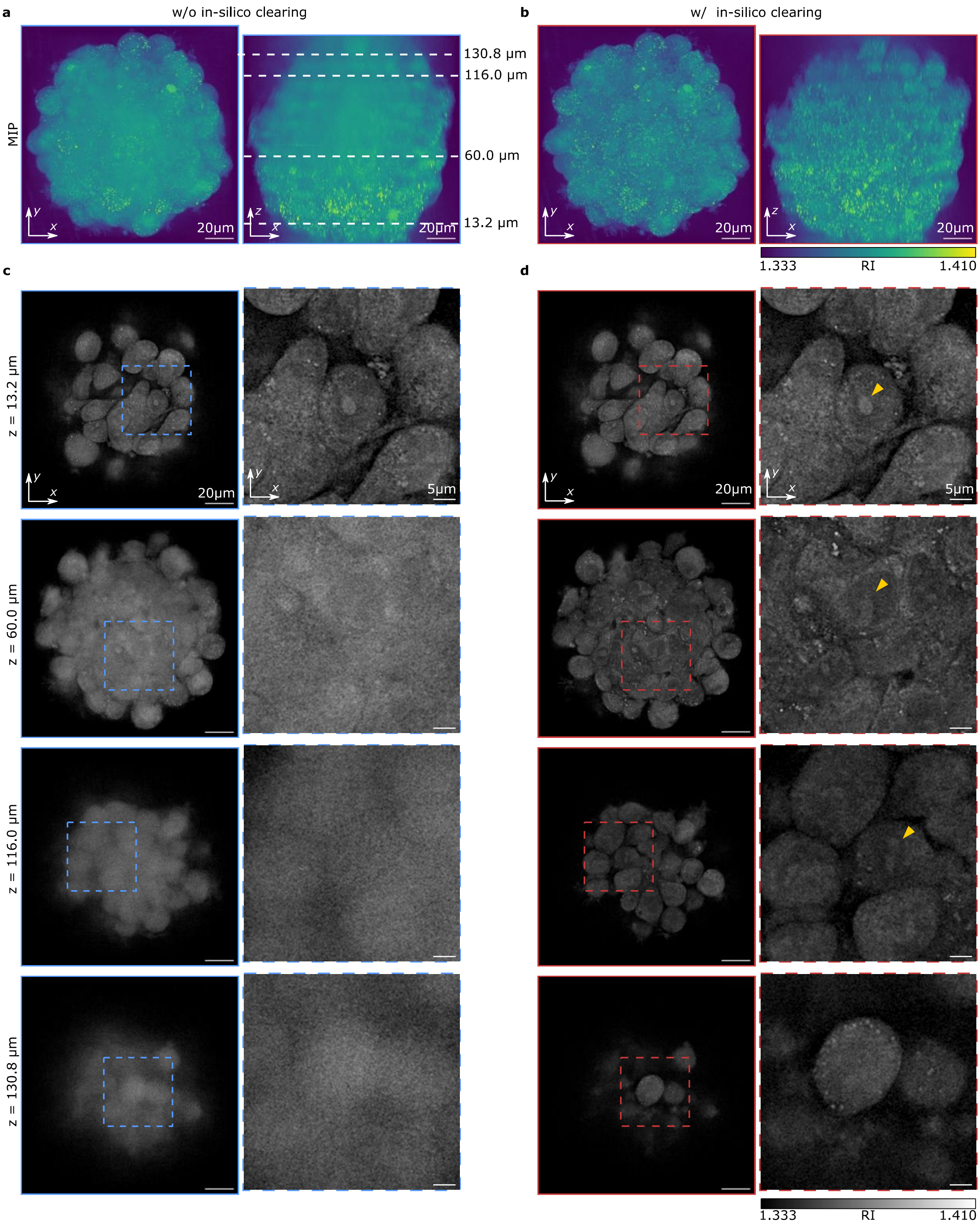

Fig. S26. Imaging an A172 spheroid. a, **b** Maximum intensity projection of the RI map reconstructed without and with in-silico clearing, respectively. **c**, **d** Cross-sections of the RI maps at different z locations (identified by white dashed lines in a), where nucleoli are identified by yellow arrowheads. In the case of in-silico clearing, the structure of the nucleoli can be confirmed up to 116.0 μm.

22. Cell culture

The human hepatoma HepG2 cell line (JCRB1054)[10], human glioblastoma A-172 cell line (JCRB0228)[11], and mouse embryocarcinoma F9 cell line (JCRB0721)[12] were cultured in Dulbecco's modified Eagle's medium (DMEM, Gibco), supplemented with 10% (v/v) FBS (Gibco), 100 U/mL of penicillin, 100 μg/mL of streptomycin, and 292 μg/mL of L-glutamine (1× penicillin-streptomycin-glutamine, Gibco). The human lung carcinoma A549 cell line (JCRB0076)[13] was cultured in a minimum essential medium (MEM, Gibco), supplemented with 10% (v/v) FBS (Gibco), 1× non-essential amino acids solution (Gibco), and 1× penicillin-streptomycin-glutamine (Gibco). The HepG2, A-172, F9, and A549 cell lines were obtained from the Japanese Collection of Research Bioresources (JCRB) cell bank. The human colonic carcinoma HCT116 cell line[14] was obtained from the European Collection of Authenticated Cell Cultures (ECACC) and cultured in McCoy's 5A medium (Sigma), supplemented with 10% (v/v) FBS (Gibco) and 1× penicillin-streptomycin-glutamine (Gibco). All cell lines were cultured at 37 ℃ in a humidified atmosphere of 5% CO_2.

References

1. Kang, S. *et al.* Imaging deep within a scattering medium using collective accumulation of single-scattered waves. *Nat. Photonics* **9**, 253–258 (2015).
2. Yasuhiko, O., Takeuchi, K., Yamada, H. & Ueda, Y. Multiple-scattering suppressive refractive index tomography for the label-free quantitative assessment of multicellular spheroids. *Biomed. Opt. Express* **13**, 962–979 (2022).
3. Liu, H. *et al.* SEAGLE: Sparsity-Driven Image Reconstruction Under Multiple Scattering. *IEEE Trans. Comput. Imaging* **4**, 73–86 (2018).
4. Chen, M., Ren, D., Liu, H.-Y., Chowdhury, S. & Waller, L. Multi-layer Born multiple-scattering model for 3D phase microscopy. *Optica* **7**, 394–403 (2020).
5. Festjens, N., Vanden Berghe, T. & Vandenabeele, P. Necrosis, a well-orchestrated form of cell demise: Signalling cascades, important mediators and concomitant immune response. *Biochim. Biophys. Acta - Bioenerg.* **1757**, 1371–1387 (2006).
6. Castaneda, F. & Kinne, R. K.-H. Ethanol Treatment of Hepatocellular Carcinoma: High Potentials of Low Concentrations. *Cancer Biol. Ther.* **3**, 430–433 (2004).
7. Yoon, S. *et al.* Deep optical imaging within complex scattering media. *Nat. Rev. Phys.* **2**, 141–158 (2020).
8. Badon, A. *et al.* Smart optical coherence tomography for ultra-deep imaging through highly scattering media. *Sci. Adv.* **2**, e1600370 (2016).
9. Badon, A., Boccara, A. C., Lerosey, G., Fink, M. & Aubry, A. Multiple scattering limit in optical microscopy. *Opt. Express* **25**, 28914–28934 (2017).
10. Aden, D. P., Fogel, A., Plotkin, S., Damjanov, I. & Knowles, B. B. Controlled synthesis of HBsAg in a differentiated human liver carcinoma-derived cell line. *Nature* **282**, 615–616 (1979).
11. Giard, D. J. *et al.* In Vitro Cultivation of Human Tumors: Establishment of Cell Lines Derived From a Series of Solid Tumors. *J. Natl. Cancer Inst.* **51**, 1417–1423 (1973).
12. Artzt, K. *et al.* Surface Antigens Common to Mouse Cleavage Embryos and Primitive Teratocarcinoma Cells in Culture. *Proc. Natl. Acad. Sci.* **70**, 2988–2992 (1973).
13. Lieber, M., Smith, B., Szakal, A., Nelson-Rees, W. & Todaro G. A continuous tumor-cell line from a human lung carcinoma with properties of type II alveolar epithelial cells. *Int. J. Cancer* **17**, 62–70 (1976).
14. Brattain, M. G., Fine, W. D., Khaled, F. M., Thompson, J. & Brattain, D. E. Heterogeneity of Malignant Cells from a Human Colonic Carcinoma. *Cancer Res.* **41**, 1751–1756 (1981).
